# Validating a novel measure of scientists’ disciplinary affiliation that captures its complexity and plasticity

**DOI:** 10.1371/journal.pone.0346268

**Published:** 2026-08-26

**Authors:** Gina Marie Pizzo, Aaron M. McCright, Chad Gonnerman, Troy E. Hall, Kathryn S. Plaisance, Brian Robinson

**Affiliations:** 1 Department of Statistics, Rice University, Houston, Texas, United States of America; 2 Department of Sociology and AgBioResearch, Michigan State University, East Lansing, Michigan, United States of America; 3 Department of Political Science, Public Administration, and Philosophy, University of Southern Indiana, Evansville, Indiana, United States of America; 4 Department of Forest Ecosystems and Society and Honors College, Oregon State University, Corvallis, Oregon, United States of America; 5 Department of Knowledge Integration and Department of Philosophy, University of Waterloo, Waterloo, Ontario, Canada; 6 Department of History, Political Science, and Philosophy, Texas A&M University-Kingsville, Kingsville, Texas, United States of America; Iowa State University, UNITED STATES OF AMERICA

## Abstract

Much research since the 1950s has investigated scientists’ views and behaviors. Employing data from standardized surveys, researchers often analyze how such views and behaviors vary across scientific disciplines. The results of these efforts are likely influenced by the quality of the survey instrument(s) used to measure scientists’ discipline or “disciplinary affiliation” as well as the size and quality of respondent samples. Even though disciplinary affiliation is a complex, multi-dimensional construct, most surveys over the years include only a single nominal indicator of one dimension (e.g., the discipline of respondents’ highest degree, current department, or self-identity). Only using a nominal indicator—with respondents placing themselves into distinct disciplines assumed to be internally homogeneous—limits our ability to measure variation in their disciplinary affiliation *within* nominal disciplines. We refer to this variation, or plasticity, in scientists’ disciplinary affiliation as *disciplinarity*, or the extent to which their affiliation may vary along a continuum between strong connection to their discipline’s core to broader association with their discipline’s frontier—possibly leading to boundary transgression and eventual affiliation with a different nominal discipline. We created a multi-dimensional continuous measure of disciplinarity for use in tandem with a categorical disciplinary affiliation indicator (i.e., discipline of PhD degree) to capture this variation. We included both measures in a standardized survey on scientists’ views about the nature of science and experiences in cross-disciplinary research that we administered to a large sample of 4,113 tenure-system science faculty at 83 US research universities. After offering narrative evidence of the content validity of our novel five-dimension disciplinarity instrument, we provide statistical evidence that our resulting disciplinarity scale has construct, convergent, and concurrent criterion validity for measuring the complexity and plasticity of scientists’ disciplinary affiliation. We close with suggestions for how other researchers may employ this novel instrument for their own purposes.

## 1. Introduction

The institution of science spans centuries [e.g., 1–4], has evolved much over time [e.g., 5–8], and varies considerably across space [e.g., 9–12]. Since the 1800s, *disciplines*—with purportedly unique histories and trajectories, substantive foci, methodological approaches, analytical techniques, and socio-intellectual communities [e.g., 13–15]—have been “the primary unit of internal differentiation in science” [16]. Yet, there is neither a singular definition of “disciplines” nor a universal taxonomy for classifying and organizing them [e.g., 17–21]. Indeed, many scholars caution that assumed boundaries between nominal disciplines are neither as clear nor as stable as often thought [e.g., 22–27].

Regardless of these challenges (which we address further in Sections 2.1 and 2.2), research that relies on particular measures of disciplinary affiliation proceeds. Most work investigating scientists’ views or behaviors analyzes data from standardized surveys. Even though disciplinary affiliation is a complex construct with multiple dimensions, most of these surveys include only a single nominal indicator of one dimension (e.g., the discipline of respondents’ highest degree, current department, or self-identity), which we discuss further in Section 2.3. Only using nominal indicators—with respondents placing themselves into distinct disciplines assumed to be internally homogeneous—also limits our ability to measure variation in scientists’ disciplinary affiliation *within* nominal disciplines (i.e., their disciplinary plasticity). Indeed, assuming that members of the same nominal discipline have a uniform disciplinary affiliation seems less than fully justified given results from qualitative research [e.g., 28,29] and arguments we present in Section 2.4.

We refer to this plasticity in scientists’ disciplinary affiliation as *disciplinarity*, or the extent to which their affiliation may vary along a continuum between strong connection to their discipline’s core to broader association with their discipline’s frontier—possibly leading to boundary transgression and eventual affiliation with a different nominal discipline. We created a multi-dimensional continuous measure of disciplinarity for use in tandem with a categorical disciplinary affiliation indicator (i.e., discipline of PhD degree) to capture this variation. We included both measures in a standardized survey on scientists’ views about the nature of science and experiences in cross-disciplinary research that we administered to a large sample of 4,113 tenure-system science faculty at 83 US research universities.

In this paper, we report evidence that our novel five-dimension *Disciplinarity Scale* is a valid measure of the complexity and plasticity of scientists’ disciplinary affiliation. We provide evidence of four types of measurement validity [30].

1) Our Disciplinarity Scale captures the full conceptual domain of “discipline” (**content validity**).
2) The individual indicators in this Disciplinarity Scale, which each represent a unique but related dimension of “discipline,” have an internally coherent structure (**construct validity**).
3) Disciplinarity Scale values are positively associated with the number of years since scientists’ PhD and the extent of their cross-disciplinary research experience (**convergent validity**).
4) Disciplinarity Scale values are not only positively associated with a change in nominal affiliation from PhD discipline (an instance of maximal plasticity), but they at least partially mediate the influence of years since PhD and cross-disciplinary research experience on disciplinary change (**concurrent criterion validity**).

The first three types of measurement validity assess how well this instrument captures the *complexity* of disciplinary affiliation, and the last two assess how well it captures the *plasticity* of such affiliation.

In Section 2.1, we briefly review how science studies scholars define “discipline” before characterizing the current landscape of taxonomies for classifying and organizing scientific disciplines in Section 2.2. We then review 115 survey-based studies of scientists and note the limitations with existing measures of disciplinary affiliation in Section 2.3. In Section 2.4, we argue why accurately capturing the complexity and plasticity of scientists’ disciplinary affiliations is important. After describing the sample, survey, and measures from our recent study in Section 3, we present and discuss key results on the performance of our new disciplinarity measure in Section 4. We end with some recommendations for analyzing complex and plastic disciplinary affiliations in academic research settings in Section 5.

## 2. Background and literature review

### 2.1. Science studies scholarship on scientific disciplines (or what is a discipline?)

There are historical, philosophical, and sociological literatures on the emergence, nature, and status of what we commonly refer to as “scientific disciplines.” Some of this scholarship has focused primarily on defining or discerning the main characteristics of disciplines [e.g., 17,19,21], while most work in this area accepts one or more such definitions and subsequently analyzes a range of important phenomena. Over the years, this literature has examined:

- the historical emergence of scientific disciplines [e.g., 31–35];
- the subsequent trajectories of disciplines, especially as they converge with or diverge from others [e.g., 25,36–39];
- the institutional characteristics of disciplines [e.g., 14,21,22,40,41];
- the relations between disciplines (e.g., the permeability of disciplinary boundaries, the competition for epistemic authority) [e.g., 15,23,42–45]; and
- the roles of disciplines in various cross-disciplinary (e.g., multidisciplinary, interdisciplinary, and transdisciplinary) endeavors [e.g., 46–50].

Rather than attempt a comprehensive review of this expansive and evolving literature, we instead focus on the five most common definitions of disciplines that emerged from our review. **First**, some scholars conceptualize disciplines at the most basic level in terms of *what their members study*. In this sense, disciplines align with “objects of study” or “problem constellations” [39:7]. **Second**, and more commonly, scholars define disciplines by *what their members know and how they know it*. That is, disciplines are relatively stable bodies of knowledge [e.g., 43,51] linked inextricably with the specialized skills employed to produce them [e.g., 52]. Most scholars using this definition focus more on the latter than the former, emphasizing the institutionalization of generally accepted methods and analytical techniques [e.g., 53,54]—or, as [44:751] puts it, “a set of practices by which that knowledge is acquired, confirmed, implemented, preserved, and reproduced.”

**Third**, scholars argue that disciplines are best characterized as macro-level *institutions* that demarcate intellectual boundaries and establish, regulate, and reproduce stable and recurring patterns of behavior [e.g., 13,40,52,55,56]. In this sense, disciplines form “the infrastructure of science” [14:46]. Key components of these institutions include university departments, peer-reviewed journals, and professional societies, which separately and together distribute resources and enforce behavioral expectations [15,16]. **Fourth**, scholars characterize disciplines as *social communities or networks of professionals* [e.g., 57]. In this sense, the defining feature of disciplines is their status as self-sustaining professions—what Becher [58] called “tribes”—that distribute status and rewards and instill in their members a shared intellectual and social identity [e.g., 36,59–61]. Such professions reproduce themselves through cycles of credentialing [e.g., 19,21]. That is, novice practitioners trained by senior professionals earn credentials and then gain employment at organizations that hire based on credentials. These new professionals evaluate and are evaluated by their credentialed peers, as they proceed to mentor and train the next generation of credentialed professionals. **Fifth**, scholars emphasize that disciplines are *systems of communication* of where and how they share information [e.g., 16,39,62]. In this sense, the defining features of disciplines are the specific publications (typically peer-reviewed journals, but also professional newsletters and conference proceedings) they control [e.g., 24,63–65] and the particular vocabularies and rhetorical styles accepted in such publications [e.g., 62,66].

While these five conceptualizations are analytically distinct, they often overlap in practice. Overall, their combination suggests that the conceptual domain of disciplinary affiliation is complex—an amalgam of scientists’ education and credential acquisition, knowledge creation and methodological skills, employment setting, professional networks, and publication outlets. Together they capture the full range of what it means to belong to a discipline. Yet, some of these definitions (e.g., what scholars know and how they know it; social communities or networks of professionals) may not be directly measurable with survey-based indicators.

We identify a set of indicators that together closely approximate the conceptual domain of disciplinary affiliation, as displayed in Table A in the S1 File. Each entry below states which definitions above each indicator partially addresses.

- The nature of a scientist’s PhD training (*earned PhD degree*, the anchor of their disciplinarity) aligns closely with their object of study, body of knowledge, and specialized skills (1st and 2nd definitions).
- A scientist’s self-identification (*self-identity*) also relates solidly with the first two definitions.
- The substantive focus of a scientist’s primary academic unit (*departmental affiliation*) captures association with a key institutional component and professional community (3rd and 4th).
- The professional association or society in which a scientist is most active (*professional association*) also relates strongly with the 3rd and 4th definitions.
- A scientist’s perception of how peer scientists view them (*peer perception*) captures important information about a scientist’s position in their professional community and their role in communication channels. Indeed, how peers view scientists’ knowledge domain, methodological skills, and scholarly contributions have deep implications for scientists’ status and opportunities within their discipline (4th and 5th).
- The substantive focus of the journals in which a scientist publishes most often (*journal audience*) aligns closely with a key institutional component and key communication channels (3rd and 5th).

Rather than rely upon one (or perhaps two) of these indicators separately, we argue that integrating all of them together more completely captures the full conceptual domain of scientists’ disciplinary affiliation and disciplinarity.

### 2.2. Existing taxonomies of scientific disciplines (or what are the disciplines?)

There is no singular, agreed-upon taxonomy for organizing and classifying scientific disciplines either within the USA or beyond. Rather, there are many taxonomies, each created and used for different purposes with varying strengths and weaknesses. While a complete accounting of these frameworks is beyond the scope of our study, we nevertheless describe some of these below (and in Table B in the S1 File) and then elucidate how the selection of a framework is a rather consequential decision.

Briefly, various types of organizations and agencies create frameworks for organizing and classifying scientific disciplines: e.g., national science, labor, or statistics agencies; funding agencies; professional societies; international organizations; and for-profit companies. We can classify their frameworks generally by their *intended scope* and *intended purpose*. Some organizations or agencies create schema that apply primarily, if not entirely, to scientific disciplines within their home country (e.g., Bureau of Labor Statistics [67]; Higher Education Statistics Agency [68]; National Center for Science and Engineering Statistics [69]; National Sciences and Engineering Research Council [70]; Social Sciences and Humanities Research Council [71]; Statistics Canada [72]; National Research Council [73]), while other organizations create frameworks with an inherently international, if not global scope (e.g., Digital Commons [74]; International Labor Office [75]; Organization for Economic Cooperation and Development [76]; United Nations Educational, Scientific, and Cultural Organization [77]; Web of Science [78]).

Further, these organizations and agencies design and use such typologies for different purposes. These include, for example, (a) characterizing educational/training programs [e.g., 73]; (b) enumerating credential recipients from educational/training programs [e.g., 68,69,77]; (c) organizing research funding opportunities [e.g., 70,71]; (d) tracking occupational employment [e.g., 67,72,75]; (e) measuring and reporting research and development data [e.g., 76]; (f) cataloging membership of learned or professional societies [e.g., 79,80]; and (g) classifying publications by subject matter [e.g., 74,78]. The result is a large array of frameworks with differing hierarchical levels (e.g., families of disciplines, disciplines, and sub-disciplines) and differing capacities for capturing the evolution and increasing differentiation of scientific disciplines over time and across space. Given this diversity, the decision to use one versus another framework is nontrivial.

We identify at least five challenges that researchers face when selecting a framework. **First**, some frameworks may entirely exclude disciplines that others include. For instance, OECD (2015) and ILO [75,76] exclude anthropology and geography from their respective frameworks. **Second**, some frameworks may relegate what is widely considered a unique discipline to be a sub-discipline of another discipline. For example, UNESCO [77] classifies anthropology as a sub-discipline of sociology. **Third**, frameworks differ in their characterization (and even inclusion) of ostensibly “applied” disciplines, such as agricultural sciences, medical/health sciences, or veterinary sciences. While some frameworks integrate these applied and other biological and life sciences into the same family [e.g., 69], other schemas separate these applied disciplines into different science families than families of purportedly “pure science” disciplines [e.g., 76], and still others bracket out one or more of these applied disciplines entirely [e.g., 79].

**Fourth**, a single hierarchical level in one framework may contain a mix of two or more levels of another framework. For example, the [80] sections align both with disciplines (e.g., anthropology, chemistry, and physics) and families of disciplines (e.g., biological sciences; and social, economic, and political sciences). **Fifth**, and perhaps most consequential for studies aiming to compare scientific disciplines most broadly, frameworks differ in whether they treat biology (or biological science) as a discipline with many sub-disciplines or as a family of disciplines (i.e., biological and life sciences) with many disciplines (and also many sub-disciplines). At issue here is whether a framework treats biology, chemistry, and physics as complementary disciplines at the same hierarchical level [e.g., 76] or whether specific fields *within* biology are the disciplinary equivalents of chemistry and physics [e.g., 79].

The selection of a specific framework (or an integration of compatible frameworks) is an important decision with substantial consequences for the investigation of scientific disciplines, as it shapes which disciplines are included, how they are grouped, and what comparisons are possible across areas of science. A major aim of our larger study is to analyze the views and experiences of scientists across a *large number* of different scientific disciplines (not necessarily *all* such disciplines). Further, we anchor our multi-dimensional measure of scientists’ disciplinary affiliation in their PhD training. This is a formative stage in scientists’ intellectual development—typically when core theoretical frameworks, concepts, research norms, and methodological/analytical techniques are first internalized.

Because our multi-dimensional measure of disciplinary affiliation is anchored in the academic discipline of scientists’ PhD, we required a framework that reliably classifies doctoral fields. We chose to use the NCSES [69] framework of broad fields and major fields (which we refer to as families and disciplines, respectively) as a foundation. The NCSES has employed its Classification of Instructional Programs (CIP) taxonomy to classify nearly every PhD earned from an accredited US institution since 1958. Since it is one of the longest-running and most robust frameworks for organizing and classifying the scientific disciplines of earned PhDs, it is optimal for our purposes. To build upon this foundation (as we note in Section 3.3), we cross-checked the NCSES families and disciplines with those from other major US-based frameworks [73,79,80]. Doing so helped us identify a list of 20 of the most commonly agreed-upon major scientific disciplines within three scientific families (biological and life sciences; physical and earth sciences; and social and behavioral sciences), as displayed in Table 1.

**Table 1:** Final List of 20 Scientific Disciplines Included in our Study.

Disciplinary Family	Scientific Discipline
Life Sciences	Biochemistry
	Botany and Plant Science
	Cell and Developmental Biology
	Ecology and Evolutionary Biology
	Environmental and Natural Resources Sciences
	Genetics and Genomics
	Microbiology
	Neuroscience and Neurobiology
	Zoology and Animal Sciences
Physical and Earth Sciences	Astronomy and Astrophysics
	Chemistry
	Geological and Earth Sciences
	Ocean/Marine Sciences and Atmospheric Sciences
	Physics
Social and Behavioral Sciences	Anthropology
	Economics
	Geography
	Political Science
	Psychology
	Sociology

### 2.3. Measures of disciplinary affiliation in survey-based studies

We aimed to characterize how researchers have measured scientists’ disciplinary affiliation on surveys where such measurement was at least modestly consequential (i.e., surveys aiming to differentiate respondents into one of multiple disciplines so scholars could perform comparative analyses of scientists across disciplines). We performed a nearly exhaustive search of English-language publications for all such surveys that met four selection criteria. We included surveys that (1) were administered to at least moderately sized samples (i.e., N > 250) (2) of PhD scientists (3) from at least two distinct disciplines, (4) not including clinical medicine or biomedical science. As of July 2025, this search identified 115 distinct surveys administered since 1955. For our purposes, we treat as a distinct survey any one-time survey, any common survey administered simultaneously in multiple nations, and each particular version (i.e., wave) of a survey from a multi-year project. We include the latter, because we observed that survey questions often changed across waves.

Table A in the S2 File lists all of these 115 surveys in chronological order. We offer an extended review of many characteristics of these surveys after Table A and in Figs A to L in the S2 File. We focus here on three aspects of how these surveys measured scientists’ disciplinary affiliation: (a) the dimension(s) included, (b) the type of direct or indirect indicators used, and (c) the level of scientific field disaggregation (e.g., discipline or family) represented in response options.

We found sufficient information to identify the actual disciplinary affiliation measures used in 97 of the 115 surveys since 1955. Of these 97 surveys, 57 included only one measure of disciplinary affiliation, 30 used two measures, and 10 used three measures. We coded these measures according to the six dimensions listed at the end of Section 2.1. Of these 97 surveys, the following percentages asked respondents to select or report:

- their primary academic discipline or area of expertise or, framed more concretely, their primary field of research and/or teaching (59.8%; 58 of 97),
- the discipline of their highest degree (43.3%; 42 of 97),
- the discipline of their current department (41.2%; 40 of 97),
- the discipline of their primary professional association or scholarly society (4.1%; 4 of 97), and
- the discipline of their commonly targeted journals (3.1%; 3 of 97).

We found sufficient information to determine the types of direct or indirect indicators that scholars used to measure disciplinary affiliation in 98 surveys. In 85.7% of surveys (84 of 98), researchers included one or more questions on their survey to *directly* measure respondents’ disciplinary affiliation. Briefly, 78.6% of surveys (77 of 98) included only a closed-ended (i.e., multiple choice) question(s); 3.1% of surveys (3 of 98) included only an open-ended question(s), which then required coding after data collection; and 4.1% of surveys (4 of 98) included both a closed-ended and an open-ended question. In the remaining 14.3% of cases (14 of 98), researchers captured respondents’ disciplinary affiliation *indirectly* during their sampling frame construction—without including any question measuring it on their survey. In such instances, scholars identified criteria during their sampling frame construction that they then used to place respondents into different disciplines. As such, these criteria are proxies for disciplinary affiliation: 8.2% (8 of 98) used respondents’ departmental or college affiliation; 4.1% (4 of 98) used respondents’ membership in a professional association or scholarly society; 1.0% (1 of 98) used the Journal Citation Reports journal classification category of respondents’ publications; and 1.0% (1 of 98) used respondents’ classification category in a governmental employment registry.

Finally, we located enough information to classify the level of scientific field disaggregation represented in the response categories from which respondents chose in 100 surveys. In 42.0% of surveys (42 of 100), respondents selected from a list that only included disciplines (or they were asked to report their discipline). In 31.0% of surveys (31 of 100), respondents selected from a list that only included families (i.e., aggregations of groups of similar disciplines) (or they were asked to report their family). In 27.0% of surveys, respondents selected from a list that included a mix of disciplines and families.

Our review of ~100 surveys of scientists since 1955 reveals six key limitations in how researchers have measured scientists’ disciplinary affiliation that likely introduce considerable measurement error and constrain their ability to perform disciplinary comparisons. First, a majority of surveys measured just one dimension of disciplinary affiliation. Second, there is considerable variation in how surveys have operationalized the most common dimension (self-identity). Third, most surveys use only nominal indicators that force researchers to categorize respondents into groups assumed to be homogenous. Fourth, some scholars try to capture respondents’ disciplinary affiliation indirectly during sampling frame construction without directly measuring it on their survey. Fifth, almost a third of surveys ask respondents only about their broad academic family, precluding any possibility of disciplinary comparison. Sixth, approximately one-fourth of surveys offered response categories that mixed together specific disciplines and broader families, making disciplinary comparisons problematic. As we discuss in the remainder of our paper, we designed our novel instrument to help overcome these limitations.

### 2.4. Importance of capturing complexity and plasticity in disciplinary affiliation

We presume that most scholars who have earned a PhD in US academic institutions since the 1950s have received theoretical and methodological training within a singular scientific discipline. Even as curricular requirements and advising experiences likely varied across disciplinary programs, scientists-in-training nevertheless completed doctoral apprenticeships that provided them with a mostly similar disciplinary foundation—which also included shared understanding of the scholarly norms, behavioral expectations, and ethical principles of their adopted discipline. As such, most scholars who investigate scientists and scientific disciplines deem it acceptable to characterize PhD recipients in the same nominal discipline as relatively homogeneous for the purpose of cross-disciplinary analyses.

Whether or not such an assumption about newly minted science PhDs is justified, we hold that treating scientists in the same nominal discipline as relatively homogeneous is much less defensible as time passes since their PhD year. This is because different scientists in the same nominal discipline will make crucial decisions and/or have important experiences that differentially influence the evolution of their respective disciplinary affiliations. We refer to this plasticity in scientists’ disciplinary affiliation as *disciplinarity*, which ranges from a strong connection to their discipline’s core to more fluid association with their discipline’s frontier and beyond. Major examples of these key decisions and experiences that likely shape scientists’ disciplinarity include: (a) the disciplinary or cross-disciplinary nature of scientists’ primary department(s) during their postdoctoral fellowship(s) and faculty position(s); (b) the extent to which scientists hold joint or cross appointments in other discipline-based departments or cross-disciplinary programs or centers; (c) scientists’ engagement with different discipline-based and/or cross-disciplinary scholarly societies or professional associations; (d) scientists’ relative involvement in disciplinary and/or cross-disciplinary research teams; and (e) their efforts to publish in peer-reviewed journals that range from core disciplinary outlets to broadly cross-disciplinary ones. The cumulative effect of these decisions and experiences likely is substantial variation in the disciplinary affiliations of scientists who nonetheless are categorized into the same nominal discipline.

**Fig 1:**
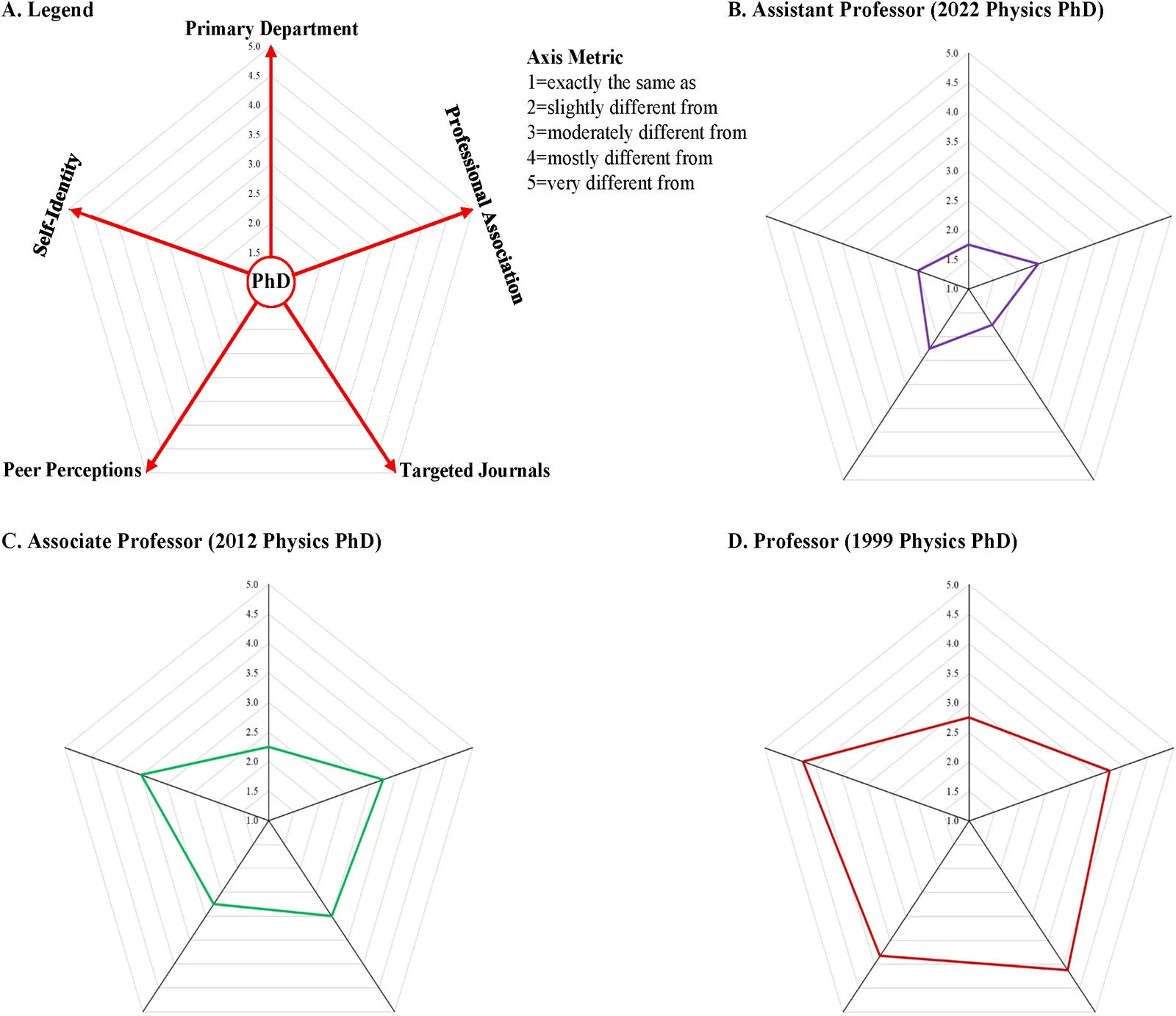
Five-Dimension Disciplinarity of Three Hypothetical Scientists.

To illustrate this point, we consider the disciplinarity of three hypothetical scientists with PhDs in physics. When answering a survey question asking them to select their disciplinary affiliation from a list of nominal categories, each likely would select “physics.” Fig 1 displays the disciplinarity of these three hypothetical physicists. At the center of each radar figure is the scientist’s PhD discipline. The five axes are the five dimensions of our disciplinarity measure, with distance from the center representing how different from their PhD discipline that each of the five facets of their current scientific work is (i.e., “exactly the same as” = 1 to “very different from” = 5). Thus, the overall area within each radar connotes the narrowness or breadth of a scientist’s disciplinarity.

Our hypothetical Assistant Professor (Panel B) is tied closely to the core of physics. That is, he works in a physics department, belongs only to the American Physical Society, and publishes only in well-established physics journals. Most peers view him as working near the core of the discipline, and he identifies as a physicist. Our hypothetical Associate Professor (Panel C) has a somewhat wider disciplinarity. While she also works in a physics department, she nevertheless is most active in the American Geophysical Union, and she primarily targets interdisciplinary climate change journals. Most peers view her work as spanning across the physical and earth sciences, and she identifies most strongly as a climate scientist. Finally, our hypothetical Professor (Panel D) has the broadest disciplinarity. They currently work in a materials science department, are most active in the Materials Research Society, and primarily publish in physical chemistry journals. Most peers know them by their work on photovoltaic cells, and they identify primarily as a renewable energy scholar.

Hypothetical examples such as these reflect the varied career trajectories that are relatively common in university science. They underscore the need for a more integrated approach to measuring disciplinary affiliation. Any single indicator—e.g., PhD degree, primary academic department, etc.—offers only a partial view, falling short of revealing the full richness of scientists’ disciplinary affiliation. To effectively capture the *complexity* and *plasticity* of scientists’ disciplinary affiliation in survey research, we argue for integrating a nominal discipline indicator (i.e., foundational PhD discipline) with a set of ordinal disciplinary plasticity indicators (our five-dimension disciplinarity measure). We maintain that this combination more fully reveals the full richness of scientists’ disciplinary affiliation. This approach is especially crucial for surveys administered to diverse samples of scientists at various stages of their life course and with different levels of cross-disciplinary research (CDR) experience and in research that aims to compare the views and behaviors of scientists across nominal disciplines.

What we discussed near the end of Section 2.1 and presented in Table A in the S1 File demonstrates how our five-dimension disciplinarity measure captures the full conceptual domain of a scientist’s discipline or disciplinary affiliation. As such, we maintain that our novel disciplinarity instrument has substantial **content validity**. This is necessary but insufficient evidence of this instrument’s measurement validity. To more fully assess the measurement validity of this instrument, we turn to more compelling analyses of its convergent, construct, and concurrent criterion validity.

## 3. Methods

### 3.1. Research design

We assessed statistical evidence of the measurement validity of our new five-dimension disciplinarity instrument with data gathered from a standardized survey administered online to a large nationally representative sample of tenure-system science faculty at major research universities in the USA. Our standardized survey is one component in a larger mixed-methods study aimed at investigating the philosophical (i.e., ontological, epistemological, and axiological) commitments of US scientists across scientific disciplines. A second component in this larger study is a content analysis of 100 university-level textbooks—five of the most widely adopted textbooks for introductory courses for each of the 20 scientific disciplines in Table 1 above. A third component is a qualitative analysis of in-depth interviews with 54 scientists—18 in each of the three major scientific families in Table 1 above.

We completed the survey component of our study in compliance with human subjects approval (STUDY00010374) provided by the Human Research Protection Program at Michigan State University. Our survey opened with a Research Participation Information and Consent Form. This explained our research aim and described what participation would entail, before informing potential respondents that their participation is voluntary, that they could skip questions they wish, and that they could end their participation at any time. After noting that respondents would face no anticipated risks while completing the survey, the form provided contact information for the team member leading the project’s survey component and Michigan State University’s Human Research Protection Program. This Research Participation Information and Consent Form ended with a question that required potential respondents to indicate whether or not they voluntarily agree to participate in the survey. Besides the basic information included in this Methods Section, we provide additional details about survey and measurement instrument design and revision, sampling frame construction, and survey administration in the S1 and S3 Files.

### 3.2. Survey design

Guided by Tailored Design Method principles [81], we followed best practices in survey research to create our novel measurement instruments [e.g., 82,83], design the structure and flow of our survey [e.g., 84,85], and manage all correspondence with potential respondents [e.g., 86,87]. Between January and August 2024, we accomplished this through a multi-stage process illustrated in Fig A in the S1 File. Briefly, this process incorporated: (1) reviews of existing measurement instruments and relevant empirical studies; (2) preliminary analyses of data from our other two study components (university-level disciplinary textbooks and interviews with university scientists); (3) feedback from our Expert Advisory Board members; (4) insights from two pilot tests of our novel measurement instruments; and (5) guidance from cognitive interviews with six scientists, using a mixed “think aloud” and probing approach [e.g., 81,88].

In addition to using conventional survey questions for measuring scientists’ PhD year and academic rank, as well as their social, demographic, and political characteristics, we created several new instruments to measure scientists’ disciplinary affiliation and cross-disciplinary research experiences, among others. Online administration via the Qualtrics platform allowed us to randomize the order of items within multi-item questions to eliminate question order effects [e.g., 89,90], incorporate skip logic to automatically guide respondents through the survey based upon their answers to earlier questions [e.g., 81,91], and pipe text from earlier responses into subsequent questions [e.g., 84,91].

### 3.3. Selection of scientific disciplines

We constructed a list of major scientific disciplines from within three selected STEM families: biological and life sciences; physical and earth sciences; and social and behavioral sciences. For the purpose of our larger study, we intentionally bracket out engineering, mathematics, computer and information science, and interdisciplinary fields. Within the biological and life sciences family, we excluded from further consideration those disciplines that are primarily clinical health- or medicine-focused and most likely located within colleges of human medicine, nursing, and veterinary medicine. With these guidelines in place, we generated an initial list of relevant disciplines by first enumerating all of the disciplines in four prominent US schemas, as noted at the end of Section 2.2: National Center for Science and Engineering Statistics [69] broad and major fields; National Research Council [73] taxonomy of fields; American Academy of Arts and Sciences [79] classes and sections; and American Association for the Advancement of Science [80] sections.

We identified the most common disciplines across the four schemas (see Table C in the S1 File). For those on only one list, we used our expertise to decide whether a discipline should be included, excluded, divided into more than one, or merged into another. Our aim was not to develop an exhaustive list of all scientific disciplines but rather create a list of ~20 major, distinct, and clearly recognized disciplines across the three disciplinary families that we could include in our study. We used three criteria when deciding which scientific disciplines to include in our final list. First, we aimed to include a set of varied disciplines to capture maximal variation in scientists’ philosophical commitments (the focus of our larger study). Second, we aimed to avoid disciplines for which we were unlikely to draw a sufficiently large sample, since there are either too few university departments or scientists (e.g., linguistics, demography). Third, we aimed to avoid disciplines that are not commonly considered scientific (e.g., public administration). Building upon the four prominent schema and employing the three criteria above, we produced the final list of 20 scientific disciplines displayed earlier in Table 1. To accurately classify sub-disciplines within these 20 disciplines, we used the updated Classification of Instructional Programs (CIP) taxonomy employed by NCSES [92]:Table A-5) in its *2021 Survey of Earned Doctorates*. Briefly, we systematically reduced the overall number of corresponding CIP codes through the four steps described in Table D in the S1 File. Completion of this process resulted in the final list of 138 sub-disciplines within our 20 scientific disciplines displayed in Table E in the S1 File.

### 3.4. Sampling design and sampling frame construction

**Fig 2:**
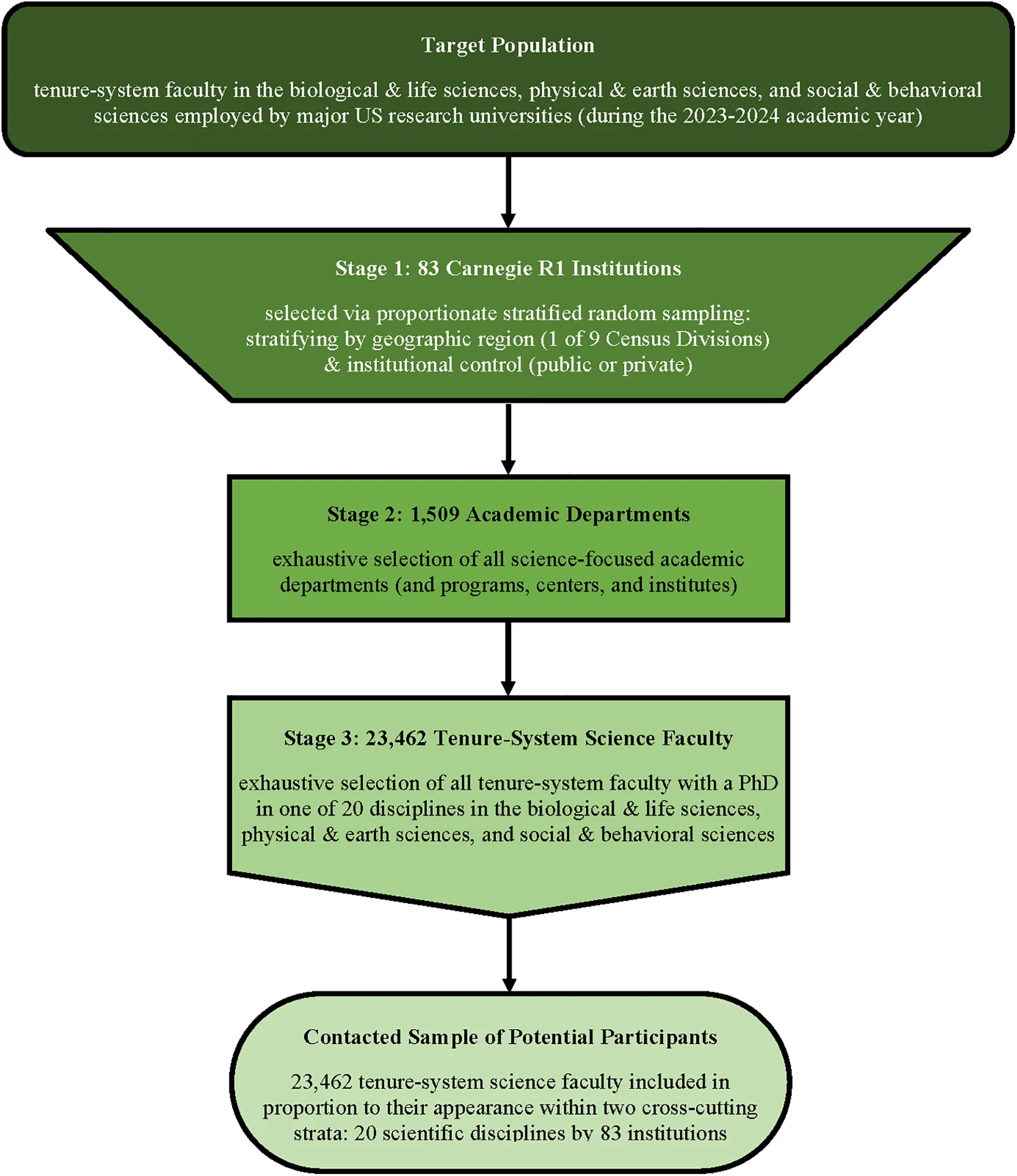
Three-Stage Construction of Sampling Frame.

We present key details of our national sampling design in Fig 2 and additional information in Table A in the S3 File. Briefly, we employed a three-stage sampling design with proportionate stratified random sampling in the first and third stages. Multi-stage sampling is optimal and cost-effective with a geographically dispersed target population for which a comprehensive list does not exist [e.g., 81]. Further, proportionate stratified random sampling (i.e., dividing groups into similar or known sub-groups and then taking a simple random sample from each sub-group in proportion to its size in the larger group) reduces the likelihood of under- or over-sampling on certain unit characteristics that might otherwise happen with simple random sampling [e.g., 87].

Our study’s target population includes all tenure-system faculty with a PhD in the biological and life sciences, physical and earth sciences, or social and behavioral sciences who are employed at a major research university in the USA. Earning a terminal degree is crucial, since doctoral training is when scientists learn the norms of their respective disciplines and develop their own philosophical commitments for performing scientific research. Further, tenure-system faculty (especially those at Carnegie R1 institutions) have consistently high expectations for research productivity.

Yet, there exists no publicly available, comprehensive list of such tenure-system faculty at any US institution—let alone for the entire country. We overcame this challenge, and created a novel sampling frame that closely approximated our target population, through the strategic deployment of tools and effort over seven months. Briefly, we created our sampling frame through a three-stage process of: selecting **institutions** (Stage 1); identifying all **departments** (or schools, programs, institutes, and centers) in which scientists may be housed at these institutions (Stage 2); and generating lists of tenure-system **faculty** with a PhD in one of our 20 scientific disciplines who were employed in any of these departments during the 2023–2024 academic year (Stage 3). Table B in the S3 File provides additional information about each sampling unit in each of these stages.

In **Stage 1**, we used stratified proportionate random sampling to select 83 of the 146 Carnegie R1 institutions in spring 2024. We stratified by two features—geographic region (9 Census Divisions) and institutional control (public or private)—to ensure our sampling frame did not over- or under-represent scientists from certain regions of the country or from either public or private institutions. Table C in the S3 File displays this stratification scheme for selecting Stage 1 institutions, and Table D in the S3 File lists all of the **83 Stage 1 institutions**. Further, Table E in the S3 File compares our 83 Stage 1 institutions with the 63 Carnegie R1 institutions not in our sampling frame on 13 institutional criteria (e.g., student enrollment, faculty employment, and NSF funding data). The lack of a statistically significant difference in any of these mean- or proportion-based criteria indicates no Stage 1 sampling frame bias.

In **Stage 2**, we created an exhaustive list of all science-focused academic departments (and schools, programs, centers, and institutes) in which a tenure-system faculty with a PhD in one of our 20 scientific disciplines might have an appointment. We followed the selection guidelines presented in Table F in the S3 File to cast a wide net (so as not to overlook any academic units that may house our tenure-system faculty of interest) and to accommodate the varying hierarchical structures across institutions. Following these selection guidelines resulted in a total of **1,509 academic units** (hereafter, departments). The fourth column in Table G in the S3 File presents the number of Stage 2 departments in each Stage 1 institution.

In **Stage 3**, we generated an exhaustive list of all tenure-system faculty with PhDs in our 20 selected scientific disciplines who were employed at our 83 selected institutions during the 2023–2024 academic year. Table H in the S3 File provides details about the ordered processes we employed in Stage 3: (a) automated web scraping of department directories and faculty biography pages to extract each tenure-system faculty’s name; rank; email address; PhD year, institution, and discipline; etc.; (b) manual data recording and cleaning to supply any missing information and confirm or revise any extracted information; and (c) manual quality control checks to eliminate duplicate entries (e.g., for faculty included in multiple units) and drop retired faculty or those not in the tenure system. Following these steps described in Table H in the S3 File produced an exhaustive total of **23,462 tenure-system science faculty** at the end of Stage 3. This is a *de facto* proportionate stratified sample of such faculty—stratified by university (83) and PhD discipline (20). The fifth column in Table G in the S3 File displays the number of Stage 3 faculty in each of our Stage 1 institutions.

### 3.5. Survey administration and respondents in sample

**Fig 3:**
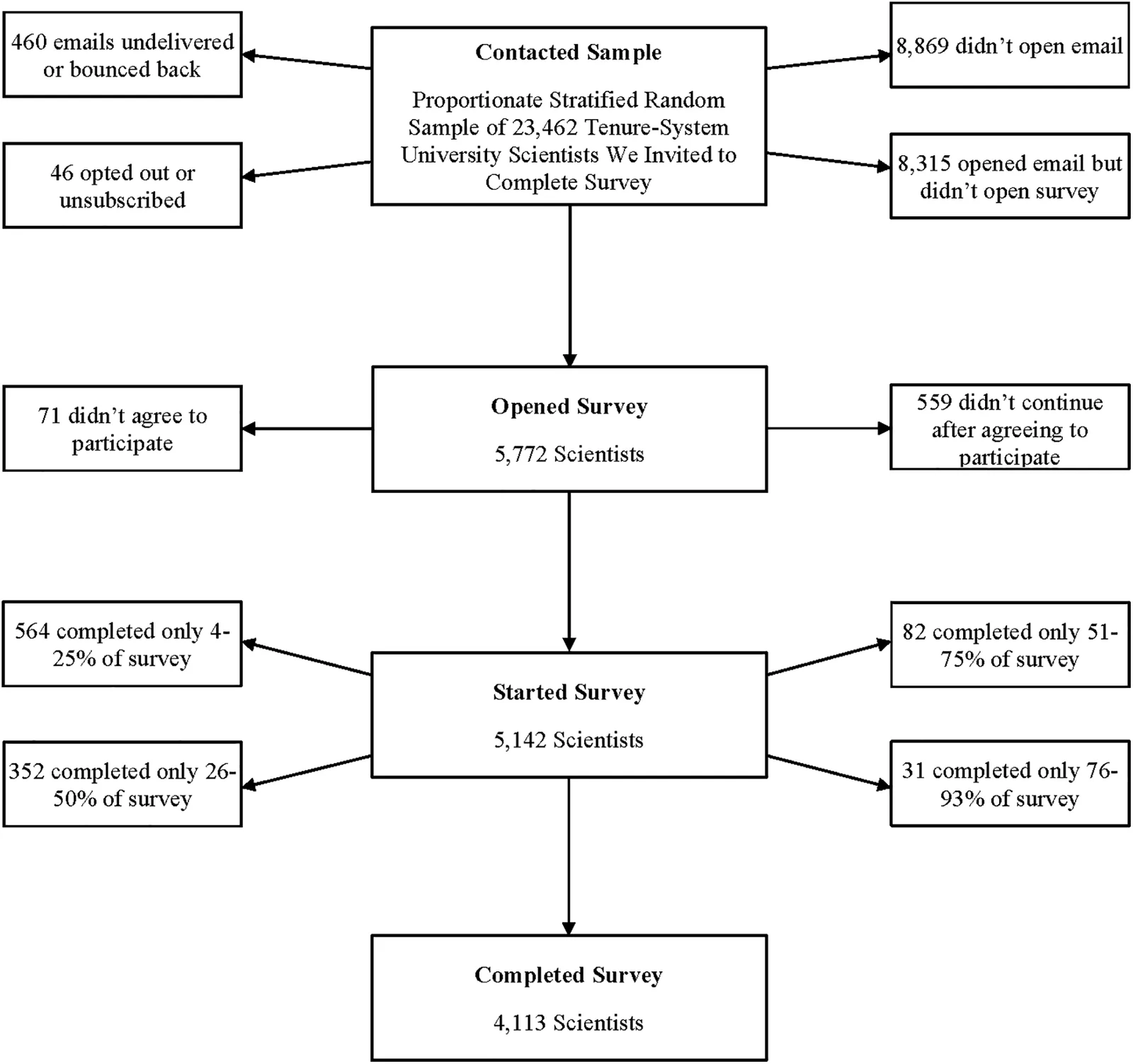
Survey Administration Process.

We administered our standardized survey online via the Qualtrics survey platform. Between September 9 and October 28, 2024, we contacted our sampling frame of 23,462 potential participants seven times—as described in Table I in the S3 File. Following Tailored Design Method principles [81], we used a combination of personalized postal mail and electronic mail correspondences to invite (and remind) scientists to participate in our study and complete our survey. Fig 3 below—and Fig A in the S3 File—displays a step-wise accounting of how our survey administration process resulted in our final sample of fully completed surveys.

Participating scientists first provided their informed consent before answering several questions about their PhD, current faculty rank, and current academic discipline. They then answered a series of questions on their philosophical commitments within three overarching domains:

- five dimensions of *ontological commitments*: theory, causation, laws, realism, and truth;
- six dimensions of *epistemological commitments*: explanation/prediction, hypothesis generation/testing, confirmation, replication, confidence, and generalizability/transferability; and
- six dimensions of *axiological commitments*: acceptability of values guiding the research process, acceptability of advocacy, objectivity, defense of the value-free ideal, the relationship between diversity and objectivity, and the social costs of error.

After responding to a few questions about their cross-disciplinary research experiences, scientists finished by answering questions about their social, demographic, and political characteristics.

The median time for survey completion was 23.13 minutes for the 4,113 scientists who completed the survey. The 95% confidence interval for the median completion time ranged from 22.63 to 23.48 minutes. Considering the 25th and 75th percentiles, respectively, half of all respondents completed the survey in between 17.45 minutes and 34.82 minutes. According to the American Association for Public Opinion Research’s (AAPOR)’s Survey Outcome Rate Calculator 5.1, this survey has a response rate (RR1) of 17.53% (4,113/23,462), which is comparable to other surveys administered to large national samples of scientists. Table 2 below provides key professional, demographic, social, and political characteristics of the national sample of tenure-system university scientists (N = 4,113) in our study.

**Table 2:** Description of National Sample of Tenure-System University Scientists (N = 4,113).

Indicator	Characteristic	% of Sample
Academic Rank	Assistant Professor	21.1
	Associate Professor	23.0
	Professor	55.9
Science Family of PhD	biological and life sciences	36.1
	physical and earth sciences	26.2
	social and behavioral sciences	37.7
Year of PhD	before 1980	6.7
	1980-1989	15.7
	1990-1999	21.6
	2000-2009	24.1
	2010-2019	26.4
	after 2019	5.5
Age	younger than 40	17.0
	40-49	26.9
	50-59	23.6
	60-69	21.1
	70 and older	11.4
Gender Identity	man	64.0
	woman	34.4
	non-binary, gender fluid, or gender non-conforming	0.7
	prefer not to say	0.9
Ethnicity	Hispanic, Latino/a/x, or Chicano/a/x	7.8
Race	White	80.4
	Black or African American	2.1
	Asian or Asian American	10.5
	Middle Eastern or North African	0.7
	American Indian or Alaska Native	0.2
	Native Hawaiian or Other Pacific Islander	0.05
	biracial or multiracial	4.9
	prefer not to say	1.2
Political Ideology	liberal	78.0
	middle-of-the-road	14.2
	conservative	7.8
Party Identification	Democrat (with “Independent, near Democrat”)	74.0
	Independent	20.5
	Republican (with “Independent, near Republican”)	5.5

### 3.6. Variables

As stated earlier, Dillman et al.’s [81] Tailored Design Method guided the creation of our novel measurement instruments and the visual design of our survey. This approach—which centers respondents by seriously considering their motivation, willingness, and ability to answer questions—features a cognitive model of the survey response process. This cognitive model of the survey response process [81:108–109], which builds upon the earlier “psychology of survey response” [e.g., 93], contains five steps through which most respondents proceed when answering survey questions on self-administered questionnaires. They clarify that respondents may work through these steps in or out of order: perception (grasping the overall layout and navigational path of a survey page); comprehension (determining what an individual question is asking); retrieval (recalling information from memory); judgment (deciding what retrieved information is most relevant/important); and response (reporting their answer within the required format). We briefly note below how this cognitive model informed the creation and presentation of our novel disciplinarity instrument.

Table F in the S1 File displays the verbatim wording of all survey questions and response categories we used to create the variables in this study. Immediately after affirming their consent to participate, respondents reported the year of their PhD (question 3 or q3). *Years since PhD* measures the amount of time since respondents earned their PhD: after 2019 = 1, 2010–2019 = 2, 2000–2009 = 3, 1990–1999 = 4, 1980–1989 = 5, and before 1980 = 6. We expected that first reporting their PhD year would aid their recall for answering subsequent questions about their PhD.

The next two questions (q4 and q5) asked respondents to select from drop-down menus the scientific family and discipline of their PhD, respectively. This question format enforces a shared understanding of “PhD family” and “PhD discipline” and of the hierarchical embeddedness of the latter within the former as codified in the Survey of Earned Doctorates and its associated Classification of Instructional Programs taxonomy. *PhD science family* is a three-category nominal variable. In some analyses, we use two dichotomous dummy variables (*PhD Biological and Life Science Family* and *PhD Social and Behavioral Science Family*), with the PhD physical and earth science family as the reference category. *PhD Science Discipline* is a nominal variable with the 20 disciplinary categories in Table 1. Several questions later we used the same two drop-down menus in asking respondents to select the scientific family and discipline they most closely identify with now (q9 and q10). *Current Science Family* and *Current Science Discipline* contain the same categories as their partnered PhD variables, respectively. *Changed Nominal Science Discipline* (no = 0, yes = 1) is a dummy variable representing whether or not respondents’ current discipline is the same as their PhD discipline.

As we discuss in Section 3.7, we modeled the two remaining constructs as latent variables with confirmatory factor analysis. After the first set of drop-down menus (for PhD scientific family and discipline) but before the second set of drop-down menus (for current scientific family and discipline), we asked each respondent about their current professional identity (q8):

“A scientist’s work often evolves throughout their career. We want to learn about the alignment between your PhD discipline and your current scientific work. Please tell us how **similar** or **different** each of the following aspects of your **current professional work** is compared to your PhD discipline of [their q5 selection].” [emphasis in original]

Our five-dimension *Disciplinarity Scale* measures the extent to which five facets of respondents’ current scientific work compares to their PhD science discipline:

- the substantive focus of their primary academic department (mean = 2.06; standard deviation = 1.20),
- the professional association in which they are most active (mean = 1.74; SD = 1.05),
- the substantive focus of the journals in which they publish most often (mean = 1.94; SD = 1.06),
- the discipline most peer scientists perceive them as representing (mean = 1.88; SD = 1.07), and
- the discipline they mostly identify with now (mean = 1.84; SD = 1.03).

Responses for each facet included: “exactly the same as” = 1, “slightly different from” = 2, “moderately different from” = 3, “mostly different from” = 4, and “very different from” = 5.

We designed the visual layout and content of this disciplinarity instrument to facilitate how respondents cognitively process survey questions and responses. Locating the question on a new page/section titled “My Professional Identity” helps respondents perceive this question place within the survey’s overall navigational path. The question stem prose elicits a direct comparison between respondents’ current professional work/identity and their PhD discipline, facilitating respondent comprehension. Including the name of respondents’ PhD discipline (from their q5 response) as piped text within the question stem also aids in question comprehension as well as the retrieval of relevant information from memory. Finally, using clear descriptors for all five response categories (i.e., “exactly the same as” to “very different from”) helps respondents judge which retrieved information is most relevant and facilitates accurate reporting of their answer.

Much later in our survey, we asked respondents about the extent to which they have had collaborative research experiences with a range of scholars. Our three-dimension *CDR Experience Scale* measures the number of research projects on which respondents have collaborated with each of the following groups of scholars: (a) other scientists within their current science discipline, (b) scientists outside their current science discipline but within their current science family, (c) scientists outside their current science family, and (d) researchers outside of science. Responses for each group of scholars included: “0 projects” = 1, “1 project” = 2, “2-3 projects” = 3, “4-5 projects” = 4, “6-7 projects” = 5, “8-9 projects” = 6, and “10 or more projects” = 7. We modeled our CDR Experience Scale using the last three indicators above (b, c, and d).

### 3.7. Analytical strategy/techniques

We managed the survey data and conducted all analyses of the convergent, construct, and concurrent criterion validity of our novel five-dimension *Disciplinarity Scale* using IBM SPSS 30.0 and R 4.5. With SPSS, we first inspected the univariate distributions of key variables and then examined the bivariate correlations among them. To assess evidence for **convergent validity** of our *Disciplinarity Scale*, we performed several statistical tests (e.g., a few tests of association and one-way ANOVAs) to investigate the relationships between this novel measure and *Years since PhD* and our *CDR Experience Scale*.

With the Latent Variable Analysis (lavaan) package in R, we employed structural equation modeling (SEM) with both observed and latent variables to assess evidence for the construct validity and concurrent criterion validity of our *Disciplinarity Scale*. We chose SEM for two key reasons. First, SEM is able to incorporate measurement modules for complex constructs (confirmatory factor analysis or CFA models) in an overall structural model [e.g., 94,95]. We took advantage of this feature by including two CFA models to measure our three-dimension *CDR Experience Scale* and five-dimension *Disciplinarity Scale* as latent variables. Supporting Text A in the S4 File provides detailed information about the CFA model for our *CDR Experience Scale*, which signifies a good fit between the latent variable and our three observed indicators we are using to measure it. The CFA results for the *Disciplinarity Scale* allow us to assess evidence for the **construct validity** of our *Disciplinarity Scale*.

**Fig 4:**
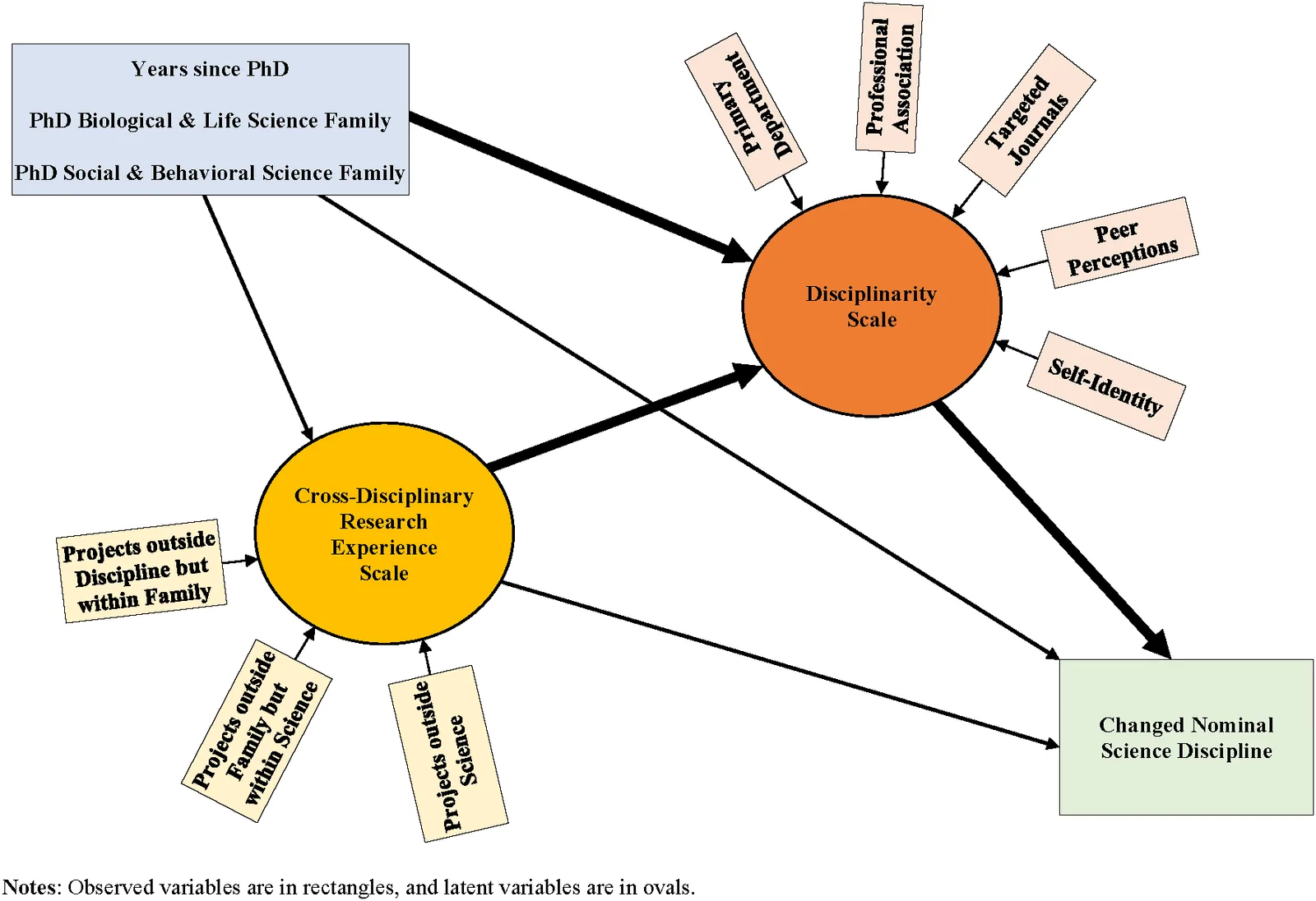
Structural Model Explaining Change in Nominal Scientific Discipline.

Second, SEM simultaneously estimates both direct and indirect effects while controlling for correlations between mediating variables—ideal for modeling mediating effects [e.g., 96,97]. This feature allows us to assess evidence for the **concurrent criterion validity** of our *Disciplinarity Scale*, by examining not only the extent to which this measure is positively associated with a change in nominal disciplinary affiliation but also the extent to which the measure at least partially mediates the influence of *Years since PhD* and *CDR Experience Scale* on *Changed Nominal Science Discipline*. Fig 4 displays our SEM structural model with two CFA measurement modules.

We assessed multivariate normality in our structural model with Mardia’s test [94], which indicated moderate skewness and kurtosis. Maximum-likelihood (ML) estimation with large samples such as ours nevertheless produces parameter estimates with asymptotic stability under moderate departures from normality [94]. As we discuss further in Section 4.3, we employed multiple indices to evaluate the fit of our structural model: Root Mean Squared Error of Approximation (RMSEA), Goodness-of-Fit Index (GFI), Comparative Fit Index (CFI), Incremental Fit Index (IFI), Tucker-Lewis Index (TLI), and Adjusted Goodness-of-Fit Index (AGFI).

## 4. Results and discussion

In Section 2.4, we argued that our novel five-dimension disciplinarity measure has substantial content validity, by demonstrating how it captures the full conceptual domain of a scientist’s discipline or disciplinary affiliation. While important, content validity nevertheless is insufficient information for establishing the measurement validity of this instrument. We now turn to three more compelling statistical analyses of measurement validity.

### 4.1. Evidence of convergent validity of the disciplinarity scale

We argued in Section 2.4 that the plasticity of scientists’ disciplinary affiliation likely is associated positively with how much time has passed since they earned their PhD and—even more so—the extent of their CDR experience. In other words, as scientists’ proceed through their academic careers and as they engage in cross-disciplinary research, we expect that the distance their current scientific work and identity is from their PhD discipline will be greater than will be the same distance among their earlier-career and less-CDR-experienced counterparts. Further, we expect the positive association between plasticity and CDR experience will be stronger than the positive association between plasticity and time since PhD.

To examine such associations, we compared scientists’ values on our Disciplinarity Scale, Years since PhD, and CDR Experience Scale. Later in Sections 4.2 and 4.3, we use CFA models to measure scientists’ disciplinarity and CDR experience in our structural SEM models. Here we use alternate measures of these two constructs for simplicity. Our Disciplinarity Scale here is a mean scale created by dividing the summed values for all dimensions by five. Our CDR Experience Scale here is an ordinal scale created by integrating scientists’ responses to the three CDR experience items in the following way: 0 total CDR projects = 1, 1–3 total CDR projects = 2, 4–6 total CDR projects = 3, 7–10 total CDR projects = 5, and 11 + total CDR projects = 5. Since this Disciplinarity Scale is a positively skewed continuous measure and Years since PhD and this CDR Experience Scale are ordinal variables, we initially employed a non-parametric test of a potential monotonic association: Spearman’s Rank Correlation (rho or ρ). Briefly, our Disciplinarity Scale is positively associated both with Years since PhD (ρ = 0.17, p < 0.001) and our CDR Experience Scale (ρ = 0.26, p < 0.001). The similar results of additional non-parametric and parametric tests of association (see Supporting Text B in the S4 File) confirm that these positive associations with Spearman’s Rank Correlation are robust.

To further demonstrate the monotonic nature of these two relationships, we ran two sets of five one-way ANOVAs to examine the associations between Years since PhD and the CDR Experience Scale and each individual dimension captured in our Disciplinarity Scale. Tables A and B in the S4 File present the results for Years since PhD and the CDR Experience Scale, respectively. Briefly, the overall pattern we found with the full Disciplinarity Scale exists for each of its five dimensions. There is a statistically significant monotonic relationship between Years since PhD and the CDR Experience Scale and each of the five dimensions of disciplinarity.

**Fig 5:**
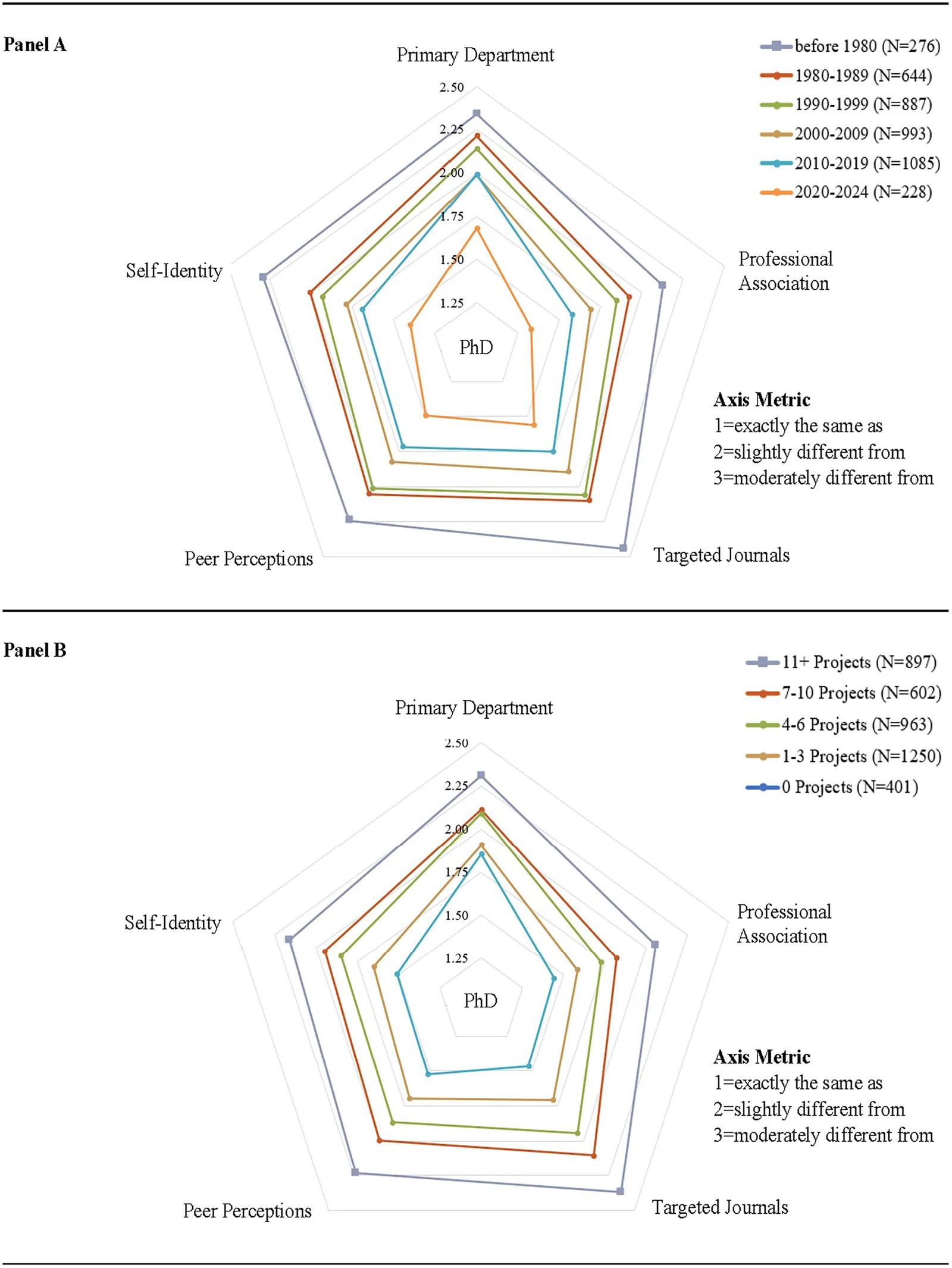
Radar Figures for Scientists’ Five-Dimension Disciplinarity Scale by Years since PhD Earned (Panel A) and Cross-Disciplinary Research Projects (Panel B).

Panels A and B in Fig 5 plot the group mean values from these two sets of five one-way ANOVAs from Tables A and B in the S4 File. Scientists’ PhD discipline sits at the center of each radar figure. The five axes represent the five dimensions of our full Disciplinarity Scale. Distance from the center along each axis represents the extent to which scientists’ current work/identity differs from their PhD discipline. Panels A and B illustrate the monotonic relationship between scientists’ disciplinarity and the years since their PhD and the extent of their CDR experience, respectively. Briefly, scientists’ disciplinary affiliation broadens over their life course and with greater CDR experience.

The results above are evidence of the convergent validity of our Disciplinarity Scale as a measure of the complexity and plasticity of scientists’ disciplinary affiliation. The extent of scientists’ disciplinary affiliation breadth (from strongly aligned with their PhD discipline’s core to broadly associated with its frontier and beyond) is indeed correlated positively with progress through their life course and—even more so—the extent of their CDR experience. Over time and with more CDR experience, scientists’ affiliation with their PhD discipline broadens, which our Disciplinarity Scale can capture.

Given our focus on scientific disciplines and the broader scientific families in which they are embedded, we also examined the extent to which the plasticity of scientists’ disciplinary affiliation may be associated with the science family of their PhD discipline and, more specifically, with their PhD discipline. Table C in the S4 File presents the results from a one-way ANOVA to examine the association between PhD Science Family and each individual dimension captured in our Disciplinarity Scale. Paralleling how we structured Fig 5, Fig A in the S4 File plots the radar figures displaying the group mean values on our five disciplinarity dimensions for each of the 20 scientific disciplines in our study.

### 4.2. Evidence of construct validity of the disciplinarity scale

We evaluated evidence of the construct validity of our Disciplinarity Scale by examining our CFA results. As noted in Section 3.7, we incorporated two measurement modules into the structural SEM model that we will discuss in Section 4.3. These are CFA models that measure our three-dimension CDR Experience Scale and five-dimension Disciplinarity Scale as latent variables. Thus, the CFA results for the latter allow us to determine the extent to which disciplinarity has an internally coherent structure following an expected theoretical pattern.

A CFA factor loading represents the strength of the relationship between the latent variable and one of its observed indicators. Essentially, it indicates how strongly that observed indicator loads onto the latent construct. Across social science generally, a factor loading (λ) greater than or equal to 0.5 is considered acceptable, and one greater than or equal to 0.7 is considered strong. Further, the squared loading (λ^2^), which acts like R^2^ in a regression model, indicates how much variation in the observed indicator is explained by the underlying factor.

Our CFA results show that our five observed indicators of disciplinarity load at least moderately strongly onto the corresponding latent construct. Further, substantial percentages of the variation in these individual indicators are explained by the underlying factor of disciplinarity. The following are the standardized loadings (λ) and squared loadings (λ^2^) for each of the five observed indicators:

- substantive focus of respondent’s primary department: λ = 0.53; λ^2^ = 0.28;
- professional association in which respondent is most active: λ = 0.80; λ^2^ = 0.64;
- substantive focus of journals in which respondent publishes most often: λ = 0.81; λ^2^ = 0.66;
- discipline that peer scientists perceive respondent as representing: λ = 0.89; λ^2^ = 0.79; and
- discipline respondent identifies with most now: λ = 0.90; λ^2^ = 0.81.

Beyond the results from Section 4.1, these CFA results provide two lines of emerging evidence suggesting that our five-dimension Disciplinarity Scale has construct validity. First, all five observed indicators moderately to strongly load onto our latent construct, forming an internally coherent structure. Indeed, four of the five indicators have especially strong factor loadings, and the remaining indicator has a moderately sized, but still acceptable, factor loading (which we contextualize in Supporting Text C in the S4 File).

Second, the fact that the first indicator above has the weakest factor loading seems to follow an expected theoretical pattern. Of the five disciplinarity indicators, scientists’ agency seems to be the most constrained on the substantive focus of their primary department. They may exercise considerable autonomy in deciding which and how many professional associations to join and peer-reviewed journals to target. Further, the myriad of choices they make throughout their career likely influence how peers perceive them and how they view themselves, and such peer perceptions and self-identity may be rather fluid. Yet, scientists have far less control over the substantive focus of their primary academic department. This is due to three factors. First, the hierarchical structures of major research universities are fairly similar due to organizational isomorphism. That is, most major research universities have largely the same array of discipline-based departments embedded in similarly structured colleges or schools. Second, there simply are not that many tenure-system science faculty positions in US research universities, so changing primary departments within universities is quite uncommon. Third, the US academic sector largely structures its labor market pools along disciplinary boundaries. These factors help explain why the indicator for the substantive focus of respondents’ primary department has the weakest factor loading of the five.

### 4.3. Evidence of concurrent criterion validity of the disciplinarity scale

Most scientists in academia remain within the discipline of their PhD throughout their career, as have approximately 83.4% of the scientists in our national sample. A survey-based categorical indicator of PhD discipline ultimately would assign all respondents in the same nominal category with an equivalent disciplinary affiliation. However, given what we have argued earlier, this approach seems less than fully justified. As such, we designed a five-dimension Disciplinarity Scale that measures the plasticity of scientists’ affiliation with their PhD discipline. In Section 4.1, we demonstrated how our novel instrument captures variation in disciplinary affiliation that ranges from a close connection to a discipline’s core to a broader association with its frontier.

At the same time, a nontrivial percentage of scientists (16.6% of our sample) eventually do leave their PhD discipline for a different nominal discipline. Supporting Text D and Fig B in the S4 File provide additional information on these scientists who have changed their discipline. We expect an instrument measuring the plasticity of scientists’ disciplinary affiliation would help discern whether or not scientists make such a disciplinary change. This would be substantive evidence of the concurrent criterion validity of our Disciplinarity Scale. Further, to the extent that procession through the life course and CDR experience increase the likelihood of such a change, we expect that our Disciplinarity Scale—by effectively capturing the complexity and plasticity of scientists’ disciplinary affiliation—would mediate the influence of these two variables on the likelihood of a nominal disciplinary change.

Our SEM structural model, displayed in Fig 4, includes Years since PhD and two science family dummy variables as observed variables and the CDR Experience Scale and Disciplinarity Scale as latent variables. The structural model has good fit as shown by a reasonably small Root Mean Squared Error of Approximation (RMSEA = 0.06) and greater-than-0.95 Goodness-of-Fit Index (GFI = 0.96). Values on alternative goodness-of-fit indices confirm this conclusion: greater-than-0.95 Comparative Fit Index (CFI = 0.96) and Incremental Fit Index (IFI = 0.96), and greater-than-0.90 Tucker-Lewis Index (TLI = 0.95), and adjusted goodness-of-fit index (AGFI = 0.94). Further, the structural model explains 29% of the variation in Changed Nominal Science Discipline. Table 3 reports the standardized coefficients from this SEM structural model explaining the likelihood of changing scientific discipline.

**Table 3:** Standardized Coefficients from Structural Equation Model Explaining the Likelihood of Changing Nominal Science Discipline (N = 4,113).

	CDR Experience			Five-Dimension Disciplinarity Scale			Changed Nominal Science Discipline		
**Independent Variables**	*Direct*	*Indirect*	*Total*	*Direct*	*Indirect*	*Total*	*Direct*	*Indirect*	*Total*
									
Life Course									
Years since PhD	0.25*		0.25*	0.12*	0.06*	0.18*	−0.04*	0.09*	0.05*
									
PhD Science Family Membership^									
PhD Biological and Life Science Family	0.11*		0.11*	0.09*	0.03*	0.11*	0.14*	0.05*	0.19*
PhD Social and Behavioral Science Family	0.04		0.04	−0.14*	0.01	−0.14*	−0.05*	−0.07	−0.11*
									
Cross-Disciplinary Research Experience									
CDR Experience Scale				0.23*		0.23*	0.00	0.11*	0.11*
									
Disciplinary Affiliation Plasticity Instrument									
Disciplinarity Scale							0.48*		0.48*
									
**Adjusted R** ^**2**^			**0.29**						
									
Goodness-of-Fit Statistics									
Root Mean Squared Error of Approximation (RMSEA)			0.06						
Goodness-of-Fit Index (GFI)			0.96						
Comparative Fit Index (CFI)			0.96						
Incremental Fit Index (IFI)			0.96						
Tucker-Lewis Index (TLI)			0.95						
Adjusted Goodness-of-Fit Index (AGFI)			0.94						

^ reference group is “PhD Physical and Earth Science Family”.

* p < .05.

As expected, Years since PhD has a positive influence on the CDR Experience Scale. As they proceed through their life course, scientists accrue more opportunities to participate in collaborative research with scholars outside of their own scientific discipline. We also find that respondents in the biological and life sciences report greater CDR experience than do those in the physical and earth sciences. Yet, social and behavioral scientists and physical and earth scientists report statistically similar CDR experience levels.

Years since PhD and the CDR Experience Scale have a positive influence on the Disciplinarity Scale, signaling an association with greater disciplinary plasticity. These results, while using CFA models to measure the two scales as latent variables, confirm the convergence validity evidence we presented in Section 4.1 when using mean-score scales to measure them. Greater progress through the life course and more CDR experience translate into greater disciplinary affiliation plasticity. Further, corroborating the additional one-way ANOVA results we discussed in endnote 13 (and presented in Table C in the S4 File), we find that physical and earth scientists report lesser disciplinary affiliation plasticity than do biological and life scientists and greater plasticity than do social and behavioral scientists.

Respondents’ progress through their life course and their CDR experience each increase the odds that they have changed their scientific discipline since earning their PhD. The total effect of the latter is approximately twice the total effect of the former. Also, respondents in the biological and life sciences are more likely, and those in the social and behavioral sciences are less likely, than their counterparts in the physical and earth sciences to have changed their scientific discipline.

More importantly, the Disciplinarity Scale has the strongest total effect in the model by a considerable margin. Broader disciplinarity substantially increase the odds of a nominal discipline change (an instance of maximal plasticity), providing clear evidence of concurrent criterion validity. (Table D in the S4 File displays the bivariate correlations between each of the five dimensions of disciplinarity and the dichotomous Changed Nominal Science Discipline. This allows us to review which factors were more or less correlated with a change in disciplinary affiliation.) Further, the Disciplinarity Scale fully mediates the influence of Years since PhD and the CDR Experience Scale on the odds that respondents have changed their scientific discipline since earning their PhD. This combination of direct and mediating effects indicates that our Disciplinarity Scale effectively captures the complexity and plasticity of scientists’ disciplinary affiliation.

## 5. Conclusion

Amid efforts to provide more interdisciplinary training opportunities within doctoral programs, more federal funding for cross-disciplinary research teams, and more university centers and institutes for promoting multidisciplinary scholarship, disciplines remain the primary unit of differentiation within science. Indeed, disciplines may be more relevant than ever [e.g., 13], even as the boundaries between them are increasingly unclear and opaque [e.g., 22–27]. Because of such major changes in science, how we measure disciplinary affiliation arguably becomes *more* consequential, and capturing within-discipline variation becomes just as critical as capturing between-discipline variation.

To this end, we created a novel survey instrument that captures the complexity of disciplinary affiliation while measuring variation in scientists’ disciplinary affiliation *within* nominal disciplines (i.e., their disciplinary plasticity or disciplinarity). In our analyses above, we offered evidence of four types of measurement validity that this five-dimension disciplinarity instrument—and our resulting Disciplinarity Scale—is a valid measure of the complexity and plasticity of scientists’ disciplinary affiliation. Briefly, our disciplinarity instrument captures the full conceptual domain of “discipline” (content validity). The resulting Disciplinarity Scale, which is comprised of five indicators each representing a distinct but related dimension of “discipline,” has an internally coherent structure (construct validity). The Disciplinarity Scale is positively associated with two constructs (years since PhD and CDR experience) in a theoretically expected manner (convergent validity). This also substantiates the patterns documented in earlier qualitative research [e.g., 28,29], providing quantitative evidence of their generalizability. Finally, not only is the Disciplinarity Scale positively associated with a change in nominal disciplinary affiliation (an instance of maximal plasticity), it also fully mediates the influence of years since PhD and CDR experience on disciplinary change (concurrent criterion validity).

Ultimately, we argue that future surveys about scientists’ views and behaviors would benefit from including both a nominal measure (or measures) of disciplinary affiliation and our ordinal instrument for measuring plasticity in disciplinary affiliation. Including both types of measures would allow researchers to more effectively capture variation in disciplinary affiliation both across and within categorical scientific disciplines. This likely would result in a more accurate accounting of scientists’ disciplinary affiliation, which will be especially helpful when examining the nuanced relationships between scientists’ disciplinary affiliation and their views about scientific and other issues and/or their behaviors. Further, including the disciplinarity instrument (a valid measure of the complexity and plasticity of scientists’ disciplinary affiliation constructed following a cognitive model of survey response) would add minimal length to a survey and produce only a negligible cognitive burden on respondents.

Among other potential uses, scholars may use this disciplinarity instrument to account for within-discipline variation in disciplinary affiliation—essentially distinguishing those scientists working closer to the frontier or boundary of their discipline from those working much closer to the core of their discipline. Scholars may accomplish this by assigning a disciplinarity score to each scientist, calculated either from their mean-score Disciplinarity Scale value or from the pentagonal area covered in their disciplinarity radar figure. Scholars may use the resulting scalar disciplinarity measure to capture disciplinary affiliation plasticity by modifying or transforming scientists’ categorical disciplinary affiliation.

## Supporting information

S1 File Supporting information on disciplinary frameworks and measurement. (DOCX)

S2 File Extended review of 115 surveys of scientists, 1955–2023. (DOCX)

S3 File National sampling design and correspondence with potential participants. (DOCX)

S4 File Additional results of data analyses. (DOCX)
