# Supplementary material for: Validating a novel measure of scientists’ disciplinary affiliation that captures its complexity and plasticity
Source: PLoS One. 2026 Aug 26;21(8):e0346268. doi: 10.1371/journal.pone.0346268 (PMC13514013; doi:10.1371/journal.pone.0346268)
Supplement: Supplementary file 1 [file pone.0346268.s001.docx]

**S1 File. Supporting Information on Disciplinary Frameworks and Measurement**

for

*Validating a novel measure of scientists’ disciplinary affiliation that captures its complexity and plasticity*

Table of Contents	Page

**Table A**	Major Pathways in Operationalizing Each Conceptualization of “Discipline” into

Survey-Based Indicators of “Disciplinary Affiliation” and “Disciplinarity”	2

**Table B**	Comparison of Selected Frameworks for Organizing and Classifying Scientific Disciplines	3

**Figure A**	Survey Design and Revision Process	4

**Table C**	Disciplines/Fields in Four Prominent Lists Remaining after Removing Those in Engineering,

Mathematics, Computer and Information Sciences, and Interdisciplinary Fields	5

**Table D**	Process for Reducing 1,610 SED-CIP Codes to the 183 SED-CIP Codes Used in Our Study	6

**Table E**	183 Academic Sub-Disciplines—and Corresponding SED-CIP Codes—in 20 Scientific

Disciplines in Three Scientific Families	8

**Table F**	Verbatim Wording of Survey Questions Used in This Study	12

**Table A**: Major Pathways in Operationalizing Each Conceptualization of “Discipline” into Survey-Based Indicators of “Disciplinary Affiliation” and “Disciplinarity”

**Conceptualization of “Discipline” in Science Studies Literature**

**what its members study**

(i.e., object of study or problem constellation

**what its members know & how they know it**

(i.e., relatively stable body of knowledge & specialized skills employed to produce it)

**macro-level institution**

(i.e., maintains an intellectual boundary, distributes resources, & enforces behavioral expectations)

**social community or network of professionals**

(self-sustaining profession that distributes status & rewards via peer evaluation & instills in members a shared identity)

**system of communication**

(where & how information is shared; control of journals with specific jargon & rhetorical styles)

**Survey-Based Indicator of “Disciplinary Affiliation” & “Disciplinarity”**

**earned PhD degree**

nature of scientist’s Ph.D. training

**self-identity**

scientist’s self-identification

**departmental affiliation**

substantive focus of scientist’s primary academic unit

**professional association**

professional association in which scientist is most active

**peer perception**

scientist’s perception of how peer scientists view them

**journal audience**

substantive focus of journal(s) in which scientist publishes most often

**Note**: We do not attempt to represent every way in which any specific survey-based indicator at least partially captures a scholarly conceptualization of “discipline.” Rather, each arrow above represents a major pathway between a particular conceptualization and a specific survey-based indicator.

**Table B**: Comparison of Selected Frameworks for Organizing and Classifying Scientific Disciplines

|  |  |  |  | **Hierarchical Levels of Framework** | | |
| --- | --- | --- | --- | --- | --- | --- |
| **Intended Scope** | **Type of Organization or Agency** | **Name of Organization or Agency** | **Name of Framework for Organizing and Classifying Scientific Disciplines** | **Family** | **Discipline or Sub-Discipline** | **Sub-Discipline or Sub-Sub-Discipline** |
| USA | national science agency | National Center for Science and Engineering Statistics | Classification of Institutional Programs (CIP) Codes | Broad Field | Major Field | Detailed Field or Fine Field |
| USA | national science agency | National Academies of Sciences | Taxonomy of Fields | Field Group | Field |  |
| USA | professional society | American Association for the Advancement of Science | List of Sections | Section | Section |  |
| USA | professional society | American Academy of Arts and Sciences | List of Classes and Sections | Class | Section |  |
| USA | national labor agency | Bureau of Labor Statistics | Standard Occupational Classification (SOC) System | Minor Group | Broad Group |  |
| United Kingdom | national education statistics agency | Higher Education Statistics Agency | Common Aggregation Hierarchy (CAH) | Level 1 and Level 2 | Level 1, Level 2, and Level 3 | Level 3 |
| Canada | national science agency | Natural Sciences and Engineering Research Council; Social Sciences and Humanities Research Council | Research Subject Codes |  | Secondary Research Subject | Primary Research Subject |
| Canada | national statistics agency | Canadian Research and Development Classification | Field of Research (FOR) Codes | Division and Group | Class | Sub-Class (Field) |
| international or global | international organization | United Nations Educational, Scientific and Cultural Organization | International Standard Classification of Education (ISCED) Fields of Education and Training | Broad Field | Narrow Field | Detailed Field |
| international or global | international organization | Organization for Economic Cooperation and Development | Revised Field of Science and Technology (FOS) Classification | Field | Sub-Field | Sub-Sub-Field |
| international or global | international organization | International Labor Office | International Standard Classification of Occupations (ISCO-08) | Minor Group | Unit Group |  |
| international or global | for-profit company | Clarivate’s Web of Science | Categories and Classification of Research Areas | Broad Category | Subject Category |  |
| international or global | for-profit company | Elsevier’s Digital Commons | Three-Tiered List of Academic Disciplines | Tier 1 | Tier 2 | Tier 3 |

**Figure A**: Survey Design and Revision Process

Review of 25 Survey-Based Studies of Scientists’ Philosophical Commitments

Review of 34 Instruments for Measuring Students’ & Teachers’ Views of the Nature of Science

Content Analysis of Transcripts from Interviews of University Scientists

Content Analysis of University Textbooks for Scientific Disciplines

Deliberations during 25 Team Meetings Produce Polished Draft of Entire Survey

Insights from two Pilot Tests of Novel Measurement Instruments

Feedback from Expert Advisory Board

Revised Drafts of Entire Survey

Insights from six “Talk-Aloud” Cognitive Interviews

Final Version of Standardized Survey Administered to Stratified Random Sample of Tenure-System Scientists at Major US Universities between September 16 and November 8, 2024

**Table C**: Families and Disciplines/Fields in Four Prominent Lists Remaining after Removing Those in Engineering, Mathematics, Computer and Information Sciences, and Interdisciplinary Fields

**American Association**

**Survey of Earned Doctorates	National Academy of Sciences	American Academy of Arts and Sciences	for the Advancement of Science**

(NCSES 2024)	(US National Research Council 2011)	(2025)	(2025)

| **Broad Field** | **Major Field** |  | **Broad Field** | **Discipline** |  | **Class** | **Specialty** |  | **AAAS Section** |
| --- | --- | --- | --- | --- | --- | --- | --- | --- | --- |
| **Life Sciences** | Agricultural sciences and natural resources |  | **Agricultural Sciences** | Animal Sciences |  | **Biological Sciences** | Biochemistry, Biophysics, and Molecular Biology |  | Biological Sciences |
|  | Biological and biomedical sciences |  |  | Entomology |  |  | Cellular and Developmental Biology |  | Neuroscience |
|  | Health sciences |  |  | Food Science |  |  | Neurosciences |  | Agriculture, Food, and Renewable Resources |
| **Physical Sciences and Earth Sciences** | Chemistry |  |  | Forestry and Forest Sciences |  |  | Evolution and Ecology |  | Medical Science |
|  | Geosciences, atmospheric sciences, and ocean sciences |  |  | Nutrition |  |  | Medical Sciences |  | Dentistry and Oral Health Sciences |
|  | Physics and astronomy |  |  | Plant Sciences |  |  | Microbiology and Immunology |  | Pharmaceutical Sciences |
| **Psychology and Social Sciences** | Psychology |  | **Biological and Health Sciences** | Biochemistry, Biophysics, and Structural Biology |  | **Mathematical and Physical Sciences** | Astronomy, Astrophysics, and Earth Sciences |  | Astronomy |
|  | Anthropology |  |  | Cell and Developmental Biology |  |  | Chemistry |  | Chemistry |
|  | Economics |  |  | Ecology and Evolutionary Biology |  |  | Physics |  | Geology and Geography |
|  | Political science and government |  |  | Public Health |  | **Social and Behavioral Sciences** | Anthropology and Archaeology |  | Physics |
|  | Sociology |  |  | Genetics and Genomics |  |  | Economics |  | Atmospheric and Hydrospheric Sciences |
|  | Other social sciences |  |  | Immunology and Infectious Diseases |  |  | Education |  | Anthropology |
|  |  |  |  | Biology/Integrated Biomedical Sciences |  |  | Law |  | Psychology |
|  |  |  |  | Kinesiology |  |  | Political Science |  | Social, Economic, and Political Sciences |
|  |  |  |  | Microbiology |  |  | Psychological Sciences |  |  |
|  |  |  |  | Neuroscience and Neurobiology |  |  | Sociology, Demography, and Geography |  |  |
|  |  |  |  | Nursing |  |  |  |  |  |
|  |  |  |  | Pharmacology, Toxicology, and Environmental Health |  |  |  |  |  |
|  |  |  |  | Physiology |  |  |  |  |  |
|  |  |  | **Physical and Mathematical Sciences** | Astrophysics and Astronomy |  |  |  |  |  |
|  |  |  |  | Chemistry |  |  |  |  |  |
|  |  |  |  | Earth Sciences |  |  |  |  |  |
|  |  |  |  | Oceanography, Atmospheric Sciences, and Meteorology |  |  |  |  |  |
|  |  |  |  | Physics |  |  |  |  |  |
|  |  |  | **Behavioral Sciences and Social Sciences** | Agricultural and Resource Economics |  |  |  |  |  |
|  |  |  |  | Anthropology |  |  |  |  |  |
|  |  |  |  | Communication |  |  |  |  |  |
|  |  |  |  | Economics |  |  |  |  |  |
|  |  |  |  | Geography |  |  |  |  |  |
|  |  |  |  | Linguistics |  |  |  |  |  |
|  |  |  |  | Political Science |  |  |  |  |  |
|  |  |  |  | Public Affairs, Public Policy, and Public Administration |  |  |  |  |  |
|  |  |  |  | Psychology |  |  |  |  |  |
|  |  |  |  | Sociology |  |  |  |  |  |

**Table D**: Process for Reducing 1,610 SED-CIP Codes to the 183 SED-CIP Codes Used in Our Study

The National Center for Science and Engineering Statistics administers the Survey of Earned Doctorates (SED) each year. Its 2021 report (*Doctorate Recipients from U.S. Universities: 2021*) includes a technical table (Table A-5: *SED-CIP Codes in New Broad, Major, and Detailed Fields: 2021*) that contains 1,610 Classification of Instructional Program (CIP) codes to classify the substantive domain of all doctorates earned within US institutions that year. We employed the following steps to delete all CIP codes except those directly relevant to one of our 20 scientific disciplines.

**Step 1**: We deleted all entries in the following “New Broad Field” categories.

| **New Broad Field** | **# of Entries Deleted** |
| --- | --- |
| computer and information sciences | 34 |
| engineering | 142 |
| health sciences | 262 |
| mathematics and statistics | 23 |
| multidisciplinary/interdisciplinary sciences | 46 |
| business | 106 |
| education | 113 |
| humanities | 147 |
| visual and performing arts | 69 |
| other non-science and engineering | 248 |
| **Subtotal Removed** | **1190** |

**Step 2**: We deleted all entries in the following “New Major Field” categories.

| **New Major Field** | **# of Entries Deleted** |
| --- | --- |
| area, ethnic, cultural, gender, and group studies | 50 |
| bioinformatics, biostatistics, and computational biology | 4 |
| microbiology and immunology | 7 |
| pharmacology and toxicology (e.g., immunology, virology, parasitology, etc.) | 8 |
| physiology, oncology, and cancer biology | 13 |
| public policy analysis | 5 |
| materials science | 2 |
| **Subtotal Removed** | **89** |

**Step 3**: We deleted all entries in the following “New Detailed Field” categories.

| **New Detailed Field** | **# of Entries Deleted** |
| --- | --- |
| veterinary biomedical and clinical sciences | 17 |
| food sciences and technology (food science; food technology and processing; brewing science; viticulture and enology; zymology/fermentation science) | 5 |
| epidemiology; epidemiology and biostatistics | 2 |
| criminal justice and corrections | 20 |
| criminology | 1 |
| applied linguistics | 2 |
| linguistics | 1 |
| archaeology (HUMANITIES) | 1 |
| international relations and national security studies | 3 |
| social sciences nec (not elsewhere classified) | 12 |
| **Subtotal Removed** | **64** |

**Step 4**: We deleted the following SED-CIP Title entries.

| **SED-CIP Title** | **# of Entries Deleted** |
| --- | --- |
| “other” categories across various disciplines | 38 |
| animal health and animal nutrition from “new detailed field” of animal sciences (plant protection and integrated pest management; range science and management) | 2 |
| 2 entries from “new detailed field” of plant sciences | 2 |
| 3 entries from “new detailed field” of environmental/natural resources management and policy (environmental/natural resources law enforcement and protective services; energy and environmental policy; environmental/natural resource recreation and tourism) | 3 |
| plant pathology/phytopathology (AGRICULTURAL SCIENCES) from “new detailed field” of plant pathology/phytopathology | 1 |
| 2 entries from “new detailed field” of forestry (wood science and wood products/pulp and paper technology; forest technology) | 2 |
| 3 entries from “new detailed field” of agricultural, animal, plant, and veterinary sciences nec (agriculture, general; aquaculture; crop production) | 3 |
| biotechnology and molecular medicine within “new detailed field” of biological and biomedical sciences | 2 |
| biomedical sciences, general | 1 |
| biology/biological sciences, general | 1 |
| biophysics (physics) within “new broad field” of biological and biomedical sciences | 1 |
| environmental studies from “new detailed field” of natural resources conservation and research | 1 |
| physical sciences, general; physics and astronomy | 2 |
| educational psychology (education) and school psychology (education) | 2 |
| environmental/natural resource economics (natural resources/conservation sciences) | 1 |
| management- or business-related categories within “new broad field” of agriculture and natural resources (agricultural business and management, general; agribusiness/agricultural business operations; farm/farm and ranch management; agricultural/farm supplies retailing and wholesaling; agricultural business technology; agricultural business and management, other; agricultural mechanization, general; agricultural power machinery operation; agricultural mechanics and equipment/machine technology; irrigation management technology; agricultural mechanization, other; agricultural production operations, general; agricultural and food products processing; applied horticulture/horticulture operations, general; greenhouse operations and management; landscaping and groundskeeping; plant nursery operations and management; turf and turfgrass management; floriculture/floristry operations and management; international agriculture; agricultural and extension education services; agricultural communication/journalism) | 22 |
| **Subtotal Removed** | **84** |

| **Remaining SED-CIP Entries** | **183** |
| --- | --- |

**Table E**: 183 Academic Sub-Disciplines—and Corresponding SED-CIP Codes—in 20 Scientific Disciplines in Three Scientific Families

| **Family** | **Discipline** | **Sub-Discipline (SED-CIP Title)** | **SED-CIP Code** |
| --- | --- | --- | --- |
| **Biological and Life Sciences** | Biochemistry | Biochemistry | 26.0202 |
|  |  | Biophysics (BIOLOGY) | 26.0203a |
|  |  | Molecular Biology | 26.0204 |
|  |  | Molecular Biochemistry | 26.0205 |
|  |  | Molecular Biophysics | 26.0206 |
|  |  | Structural Biology | 26.0207 |
|  |  | Photobiology | 26.0208 |
|  |  | Radiation Biology/ Radiobiology | 26.0209 |
|  |  | Biochemistry and Molecular Biology | 26.0210 |
|  | Botany and Plant Science | Agroecology and Sustainable Agriculture | 01.0308 |
|  |  | Apiculture | 01.0310 |
|  |  | Ornamental Horticulture | 01.0603 |
|  |  | Public Horticulture | 01.0609 |
|  |  | Urban and Community Horticulture | 01.0610 |
|  |  | Plant Sciences, General | 01.1101 |
|  |  | Agronomy and Crop Science | 01.1102 |
|  |  | Horticultural Science | 01.1103 |
|  |  | Agricultural and Horticultural Plant Breeding | 01.1104 |
|  |  | Soil Science and Agronomy, General | 01.1201 |
|  |  | Soil Chemistry and Physics | 01.1202 |
|  |  | Soil Microbiology | 01.1203a |
|  |  | Soil Chemistry and Microbiology | 01.1203b |
|  |  | Botany/ Plant Biology | 26.0301 |
|  |  | Plant Pathology/ Phytopathology (BIOLOGY) | 26.0305a |
|  |  | Plant Physiology | 26.0307 |
|  |  | Plant Molecular Biology | 26.0308 |
|  | Cell and Developmental Biology | Cell/ Cellular Biology and Histology | 26.0401 |
|  |  | Anatomy | 26.0403 |
|  |  | Developmental Biology and Embryology | 26.0404 |
|  |  | Cell/ Cellular and Molecular Biology | 26.0406 |
|  |  | Cell Biology and Anatomy | 26.0407 |
|  | Ecology and Evolutionary Biology | Ecology | 26.1301 |
|  |  | Evolutionary Biology | 26.1303 |
|  |  | Aquatic Biology/ Limnology | 26.1304 |
|  |  | Environmental Biology | 26.1305 |
|  |  | Population Biology | 26.1306 |
|  |  | Conservation Biology | 26.1307 |
|  |  | Systematic Biology/ Biological Systematics | 26.1308 |
|  |  | Ecology and Evolutionary Biology | 26.1310 |
|  | Environmental and Natural Resources Sciences | Natural Resources/ Conservation, General | 03.0101 |
|  |  | Environmental Science | 03.0104 |
|  |  | Environmental/ Natural Resources Management and Policy, General | 03.0201 |
|  |  | Water, Wetlands, and Marine Resources Management | 03.0205 |
|  |  | Land Use Planning and Management/ Development | 03.0206 |
|  |  | Bioenergy | 03.0210 |
|  |  | Fishing and Fisheries Sciences and Management | 03.0301 |
|  |  | Forestry, General | 03.0501 |
|  |  | Forest Sciences and Biology | 03.0502 |
|  |  | Forest Management/ Forest Resources Management | 03.0506 |
|  |  | Urban Forestry | 03.0508 |
|  |  | Forest Resources Production and Management | 03.0510 |
|  |  | Wildlife, Fish and Wildlands Science and Management | 03.0601 |
|  | Genetics and Genomics | Genetics, General | 26.0801 |
|  |  | Molecular Genetics | 26.0802 |
|  |  | Microbial and Eukaryotic Genetics | 26.0803 |
|  |  | Animal Genetics | 26.0804 |
|  |  | Plant Genetics | 26.0805 |
|  |  | Human/ Medical Genetics | 26.0806 |
|  |  | Genome Sciences/ Genomics | 26.0807 |
|  | Microbiology | Microbiology, General | 26.0502 |
|  | Neuroscience and Neurobiology | Neuroscience | 26.1501 |
|  |  | Neuroanatomy | 26.1502 |
|  |  | Neurobiology and Anatomy | 26.1503 |
|  |  | Neurobiology and Behavior | 26.1504 |
|  |  | Cognitive Neuroscience | 26.1599b |
|  | Zoology and Animal Sciences | Animal/ Livestock Husbandry and Production | 01.0302 |
|  |  | Dairy Husbandry and Production | 01.0306 |
|  |  | Horse Husbandry/ Equine Science and Management | 01.0307 |
|  |  | Animal Sciences, General | 01.0901 |
|  |  | Agricultural Animal Breeding | 01.0902 |
|  |  | Dairy Science | 01.0905 |
|  |  | Livestock Management | 01.0906 |
|  |  | Poultry Science | 01.0907 |
|  |  | Zoology/ Animal Biology | 26.0701 |
|  |  | Entomology | 26.0702a |
|  |  | Nematology | 26.0702b |
|  |  | Animal Physiology | 26.0707 |
|  |  | Animal Behavior and Ethology | 26.0708 |
|  |  | Wildlife Biology | 26.0709 |
| **Physical and Earth Sciences** | Astronomy and Astrophysics | Astronomy | 40.0201 |
|  |  | Astrophysics | 40.0202 |
|  |  | Planetary Astronomy and Science | 40.0203 |
|  | Chemistry | Chemistry, General | 40.0501 |
|  |  | Analytical Chemistry | 40.0502 |
|  |  | Inorganic Chemistry | 40.0503 |
|  |  | Organic Chemistry | 40.0504 |
|  |  | Physical Chemistry | 40.0506 |
|  |  | Polymer Chemistry | 40.0507 |
|  |  | Chemical Physics | 40.0508 |
|  |  | Environmental Chemistry | 40.0509 |
|  |  | Forensic Chemistry | 40.0510 |
|  |  | Theoretical Chemistry | 40.0511 |
|  |  | Cheminformatics/ Chemistry Informatics | 40.0512 |
|  |  | Chemistry, Other | 40.0599a |
|  |  | Chemical Biology | 40.0599b |
|  | Geological and Earth Sciences | Surveying Technology/ Surveying | 15.1102 |
|  |  | Earth Systems Science | 30.3801 |
|  |  | Environmental Geosciences | 30.4101 |
|  |  | Geology/ Earth Science, General | 40.0601a |
|  |  | Earth Science | 40.0601b |
|  |  | Geology | 40.0601c |
|  |  | Geochemistry | 40.0602 |
|  |  | Geophysics and Seismology | 40.0603 |
|  |  | Paleontology | 40.0604 |
|  |  | Hydrology and Water Resources Science | 40.0605 |
|  |  | Geochemistry and Petrology | 40.0606 |
|  |  | Geomorphology and Glacial Geology | 40.0699b |
|  |  | Stratigraphy and Sedimentation | 40.0699c |
|  | Ocean/Marine Sciences and Atmospheric Sciences | Marine Biology and Biological Oceanography | 26.1302 |
|  |  | Marine Sciences | 30.3201a |
|  |  | Climate Science | 30.3501 |
|  |  | Atmospheric Sciences and Meteorology, General | 40.0401 |
|  |  | Atmospheric Chemistry and Climatology | 40.0402 |
|  |  | Atmospheric Physics and Dynamics | 40.0403 |
|  |  | Meteorology | 40.0404 |
|  |  | Oceanography, Chemical and Physical | 40.0607 |
|  | Physics | Physics, General | 40.0801 |
|  |  | Atomic/ Molecular Physics | 40.0802 |
|  |  | Elementary Particle Physics | 40.0804 |
|  |  | Plasma and High-Temperature Physics | 40.0805 |
|  |  | Nuclear Physics | 40.0806 |
|  |  | Optics/ Optical Sciences | 40.0807 |
|  |  | Condensed Matter and Materials Physics | 40.0808a |
|  |  | Polymer Physics | 40.0808b |
|  |  | Acoustics | 40.0809 |
|  |  | Theoretical and Mathematical Physics | 40.0810 |
|  |  | Applied Physics | 40.0899b |
| **Social and Behavioral Sciences** | Anthropology | Anthropology, General | 45.0201 |
|  |  | Physical and Biological Anthropology | 45.0202 |
|  |  | Medical Anthropology | 45.0203 |
|  |  | Cultural Anthropology | 45.0204 |
|  |  | Forensic Anthropology | 45.0205 |
|  |  | Archaeology/ Archeology (SOCIAL SCIENCES) | 45.0301a |
|  | Economics | Agricultural Economics | 01.0103 |
|  |  | Economics, General | 45.0601 |
|  |  | Applied Economics | 45.0602a |
|  |  | Environmental/ Natural Resource Economics (SOCIAL SCIENCES) | 45.0602b |
|  |  | Econometrics and Quantitative Economics | 45.0603 |
|  |  | Development Economics and International Development | 45.0604 |
|  |  | International Economics | 45.0605 |
|  | Geography | Geography | 45.0701 |
|  |  | Geographic Information Science and Cartography | 45.0702 |
|  | Political Science | Political Science and Government, General | 45.1001 |
|  |  | American Government and Politics (United States) | 45.1002 |
|  |  | Canadian Government and Politics | 45.1003 |
|  |  | Political Economy | 45.1004 |
|  | Psychology | Human Development and Family Studies, General | 19.0701 |
|  |  | Adult Development and Aging | 19.0702 |
|  |  | Child Development | 19.0706 |
|  |  | Psychology, General | 42.0101 |
|  |  | Cognitive Psychology and Psycholinguistics | 42.2701 |
|  |  | Comparative Psychology | 42.2702 |
|  |  | Developmental and Child Psychology | 42.2703 |
|  |  | Experimental Psychology | 42.2704 |
|  |  | Personality Psychology | 42.2705 |
|  |  | Behavioral Neuroscience | 42.2706 |
|  |  | Social Psychology | 42.2707 |
|  |  | Psychometrics and Quantitative Psychology | 42.2708 |
|  |  | Psychopharmacology | 42.2709 |
|  |  | Developmental and Adolescent Psychology | 42.2710 |
|  |  | Physiological/ Psychobiology Psychology | 42.2799b |
|  |  | Clinical Psychology | 42.2801 |
|  |  | Community Psychology | 42.2802 |
|  |  | Counseling Psychology | 42.2803 |
|  |  | Industrial and Organizational Psychology | 42.2804 |
|  |  | School Psychology (PSYCHOLOGY) | 42.2805a |
|  |  | Educational Psychology (PSYCHOLOGY) | 42.2806a |
|  |  | Clinical Child Psychology | 42.2807 |
|  |  | Environmental Psychology | 42.2808 |
|  |  | Geropsychology | 42.2809 |
|  |  | Health/ Medical Psychology | 42.2810 |
|  |  | Family Psychology | 42.2811 |
|  |  | Forensic Psychology | 42.2812 |
|  |  | Applied Psychology | 42.2813 |
|  |  | Applied Behavior Analysis | 42.2814 |
|  |  | Performance and Sport Psychology | 42.2815 |
|  |  | Somatic Psychology | 42.2816 |
|  |  | Transpersonal/ Spiritual Psychology | 42.2817 |
|  | Sociology | Sociology, General | 45.1101 |
|  |  | Applied/ Public Sociology | 45.1102 |
|  |  | Rural Sociology | 45.1103 |
|  |  | Demography and Population Studies | 45.0501 |
|  |  | Applied Demography | 45.0502 |

**Source**: Table A-5: *SED-CIP Codes in New Broad, Major, and Detailed Fields: 2021* in NCSES (2022).

National Center for Science and Engineering Statistics (NCSES). 2022. *Doctorate Recipients from U.S. Universities: 2021*. NSF 23-300. Alexandria, VA: US National Science Foundation. Available at <https://ncses.nsf.gov/pubs/nsf23300>.

**Table F**: Verbatim Wording of Survey Questions Used in This Study

Years Since PhD

q3. In what year did you earn your PhD? *(If you have two, when did you earn your most recent PhD?)*

 before 1970

 1970-1974

 1975-1979

 1980-1984

 1985-1989

 1990-1994

 1995-1999

 2000-2004

 2005-2009

 2010-2014

 2015-2019

 2020-2024

 I have not earned a PhD	[drop from survey if selected]

PhD Science Family and PhD Science Discipline

It is important to us that we accurately capture the **academic discipline of your PhD** (or your most recent one, if you have two). We are using the NSF’s classification system that organizes science sub-disciplines within disciplines and those disciplines within larger families.

First select the **family** in which your discipline belongs.

Within this family of disciplines, next select your **discipline**.

Within this discipline, then select your **sub-discipline**.

q4. In which **family of academic disciplines** is your PhD?

 biological and life sciences

 physical and earth sciences

 social and behavioral sciences

q5. Within this general family, what is the specific **academic discipline** of your PhD? Select the closest match.

[drop-down menu contains the alphabetized set of disciplines for their selected family from the prior question]

[question response is piped forward as [PhD discipline]]

q6. Within this specific academic discipline, what is the particular **academic sub-discipline** of your PhD? Select the closest match.

[drop down menu contains the alphabetized set of sub-disciplines (listed in Table SI4) for their selected discipline from the prior question]

Disciplinarity or Plasticity in Disciplinary Affiliation

A scientist’s work often evolves throughout their career. We want to learn more about the alignment between your PhD discipline and your current scientific work.

q8. Please tell us how **similar** or **different** each of the following aspects of your **current professional work** is compared to your PhD discipline of [PhD discipline]. [order of first four items is randomized]

exactly	slightly	moderately	mostly	very

the	different	different	different	different

same as	from	from	from	from

The substantive focus of my primary academic department is __ my PhD discipline.					

The professional association in which I am most active is __ my PhD discipline.					

The substantive focus of the journals in which I publish most often is __ my PhD discipline.					

The discipline most peer scientists perceive me as representing is __ my PhD discipline.					

The discipline I identify with most now is __ my PhD discipline.					

Current Science Family and Current Science Discipline

It is important to us that we accurately capture the **academic discipline you most closely identify with most now**, since we will be asking you some questions about it later.

q9. What **family of academic disciplines** do you most closely identify with **now**?

 biological and life sciences

 physical and earth sciences

 social and behavioral sciences

[question response is piped forward as [current family]]

q10. Within this general family, what specific **academic discipline** do you most closely identify with **now**? Select the closest match.

[drop-down menu contains the alphabetized set of disciplines for their selected family from the prior question]

[question response is piped forward as [current discipline]]

Cross-Disciplinary Research Experience

In this section, we want to learn about any research experience you have had collaborating with scholars outside of your academic discipline of [current discipline] since you have been a tenure-system faculty.

By collaborative research experience, we mean working together on scholarly activities, such as securing grant funding, collecting and analyzing data, presenting at conferences, and publishing manuscripts.

q17. While you have been a tenure-system faculty, on **how many research projects** have you collaborated with each of the following groups of scholars?

0	1	2-3	4-5	6-7	8-9	10 or more

projects	project	projects	projects	projects	projects	projects

other scientists within [current discipline]							

scientists outside [current discipline] but

within the [current family]							

scientists outside the [current family]							

researchers outside of science							
