# Supplementary material for: Validating a novel measure of scientists’ disciplinary affiliation that captures its complexity and plasticity
Source: PLoS One. 2026 Aug 26;21(8):e0346268. doi: 10.1371/journal.pone.0346268 (PMC13514013; doi:10.1371/journal.pone.0346268)
Supplement: Supplementary file 2 [file pone.0346268.s002.docx]

**S2 File. Extended Review of 115 Surveys of Scientists, 1955-2023**

for

*Validating a novel measure of scientists’ disciplinary affiliation that captures its complexity and plasticity*

Table of Contents	Page

**Table A**	115 Surveys of Scientists, 1955-2023	2

**Extended Review of 115 Surveys of Scientists, 1955-2023**	14

**Figure A**	Annual and Cumulative Number of Unique Surveys Administered to at Least Moderately

Sized Samples of Scientists, 1955-2023	23

**Figure B**	Number of Unique Surveys Administered in 56 Countries	20

**Figure C**	Number of Unique Surveys Administered in Seven Geographic or Cultural Regions	21

**Figure D**	Main Foci of 115 Unique Surveys	22

**Figure E**	General Type of Sampling Design for 115 Unique Surveys	23

**Figure F**	Cumulative Number of 113 Unique Surveys by Administration Mode, 1955-2023	24

**Figure G**	Response Rate for 63 Unique Surveys Administered in the USA, 1955-2023	25

**Figure H**	Sample Size for 69 Unique Surveys Administered in the USA, 1955-2023	26

**Figure I**	Frequency of Disciplinary Dimensions Measured in 97 Unique Surveys	27

**Figure J**	Frequency of All Pairs of Disciplinary Dimensions Measured in 30 Unique Surveys	28

**Figure K**	Types of Indicators Used to Measure Scientific Field in 98 Unique Surveys	29

**Figure L**	Percentages of Scientific Field Disaggregation Level Measured in 100 Unique Surveys	30

**Table A**: 115 Surveys of Scientists, 1955-2023

| **Survey Year(s)** | **Survey Name**  (informal in italics) | **Survey Topic(s)** | **Key Reference(s)** |
| --- | --- | --- | --- |
| 1955 | *survey of social science teaching faculty* | recent threats to academic freedom | Lazarsfeld, Paul F., & Wagner Thielens, Jr. 1957. “Social Scientists & Recent Threats to Academic Freedom.” *Social Problems* 5(3):244-256.  Lazarsfeld, Paul F., & Wagner Thielens, Jr. 1958. *The Academic Mind: Social Scientists in a Time of Crisis*. Glencoe, IL: The Free Press. |
| 1959-1964 | *surveys of professional societies* | political attitudes & behaviors | Spaulding, Charles B., Henry A. Turner, & Charles G. McClintock. 1963. “Political Orientations of Academically Affiliated Sociologists.” *Sociology & Social Research* 47:273-289.  Turner, Henry A., Charles G. McClintock, & Charles B. Spaulding. 1963. “The Political Party Affiliation of American Political Scientists.” *The Western Political Quarterly* 16:650-665.  McClintock, Charles G., Charles B. Spaulding, & Henry A. Turner. 1965. “Political Orientations of Academically Affiliated Psychologists.” *American Psychologist* 20:211-221.  Spaulding, Charles B., & Henry A. Turner. 1968. “Political Orientation & Field of Specialization among College Professors.” *Sociology of Education* 41:247-262. |
| 1962 | *faculty survey for 1963 Robbins Report* | general purpose survey | Committee on Higher Education. 1963. *Higher Education: Report of the Committee Appointed by the Prime Minister under the Chairmanship of Lord Robbins, 1961-1963*. Cmnd. 2154. London: Her Majesty’s Stationary Office.  Anonymous. 1963. “The Robbins Report.” *Education & Training* 5(12):563-565.  Morris, Charles. 1964. “The Robbins Report.” *British Journal of Educational Studies* 13(1):5-15. |
| 1964 | *survey of British university faculty* | general purpose survey | Trow, Martin, & A. H. Halsey. 1969. “British Academics & the Professorship.” *Sociology* 3(3):321-339.  Halsey, Albert Henry, & Martin A. Trow. 1971. *The British Academics*. London: Faber & Faber. |
| 1969 | Carnegie Survey of Faculty at US Colleges & Universities^1^ | general purpose survey (with emphasis on political topics) | Trow, Martin A. 1969. *Carnegie Commission National Survey of Higher Education: Faculty Study, 1969*. ICPSR 7501. Archived at Inter-university Consortium for Political & Social Research. January 25, 2008. doi:10.3886/ICPSR07501.v1.  Ladd, Everett, Seymour Martin Lipset, & Martin Trow. 1969. *Carnegie Commission National Survey of Higher Education: Faculty Study Subsample, 1969*. ICPSR 7078. Archived at Inter-university Consortium for Political & Social Research. February 16, 1992. doi:10.3886/ICPSR07078.v1.  Bayer, Alan E. 1970. *College & University Faculty: A Statistical Description*. ACE Research Reports, Volume 5, Number 5. Washington, DC: American Council on Education.  Trow, Martin. 1972. *The Distribution of Academic Tenure in American Higher Education*. Berkeley, CA: Carnegie Commission on Higher Education.  Ladd, Jr., Everett Carll, & Seymour Martin Lipset. 1972. “Politics of Academic Natural Scientists & Engineers.” *Science* 176:1091-1100.  Trow, Martin. 1975. *Teachers & Students: Aspects of American Higher Education*. New York: McGraw-Hill.  Ladd, Jr., Everett Carll, & Seymour Martin Lipset. 1975. *The Divided Academy: Professors & Politics*. New York: McGraw-Hill. |
| 1972 | Teaching Faculty in America | general purpose survey | Bayer, Alan E. 1973. *Teaching Faculty in America, 1972-1973*. American Council on Education (ACE) Research Report: Volume 8, Number 2. Washington, DC: American Council on Education. |
| 1975 | Carnegie Survey of Faculty at US Colleges & Universities^1^ | general purpose survey (with emphasis on political topics) | Ladd, Jr., Everett Carll, & Seymour Martin Lipset. 1976. *Survey of the Social, Political, & Educational Perspectives of American College & University Faculty*. Final Report. Sponsored by the National Institute of Education within the Department of Health, Education, & Welfare. Storrs, CT: University of Connecticut. |

| **Year(s)** | **Name**  (informal in italics) | **Topic(s)** | **Key Reference(s)** |
| --- | --- | --- | --- |
| 1977 | Survey of the American Professoriate | general purpose survey (with emphasis on political topics) | Ladd, Jr., Everett Carll, & Seymour Martin Lipset. 1978. *Technical Report, 1977 Survey of the American Professoriate.* Storrs, CT: University of Connecticut Social Data Center.  Braxton, John M. 1990. “Deviancy from the Norms of Science: A Test of Control Theory.” *Research in Higher Education* 31:461-476.  Braxton, John M. 1993. “Deviancy from the Norms of Science: The Effects of Anomie & Alienation in the Academic Profession.” *Research in Higher Education* 34:213-228. |
| 1984 | Carnegie Survey of Faculty at US Colleges & Universities^1^ | general purpose survey (with emphasis on political topics) | Opinion Research Corporation. 1984. *Technical Report: 1984 Carnegie Foundation National Surveys of Higher Education*. Princeton, NJ: Opinion Research Corporation.  Hamilton, & Hargens. 1993. “The Politics of the Professors: Self-Identifications, 1969-1984.” *Social Forces* 71:603-627.  Ransom, Michael R., & Sharon Bernstein Megdal. 1993. “Sex Differences in the Academic Labor Market in the Affirmative Action Era.” *Economics of Education Review* 12:21-43. |
| 1985 | *entrepreneurship among life scientists survey* | entrepreneurship among life scientists | Louis, Karen Seashore, David Blumenthal, Michael Gluck, & Michael A. Stoto. 1989. “Entrepreneurs in Academe: An Exploration of Behaviors Among Life Scientists.” *Administrative Science Quarterly* 34:110-131. |
| 1987 | Survey of the Academic Profession in Canada | general purpose survey | Lennards, Jos. 1990. *The Academic Profession in Canada*. Working Paper. Department of Sociology, Glendon College, York University.  Nakhaie, M. Reza, & Robert Brym. 1999. “The Political Attitudes of Canadian Professors.” *Canadian Journal of Sociology* 24:329-353.  Nakhaie, M. Reza. 2002. “Gender Differences in Publication among University Professors in Canada.” *Canadian Review of Sociology & Anthropology* 39(2):151-179.  Nakhaie, M. Reza. 2007. “Universalism, Ascription, & Academic Rank: Canadian Professors, 1987-2000.” *Canadian Review of Sociology & Anthropology* 44(3):361-386. |
| 1987-1988 | National Survey of Postsecondary Faculty^2^ | general purpose survey | Russell, Susan H., James S. Fairweather, & Robert M. Hendrickson. 1991. *Profiles of Faculty in Higher Education Institutions, 1988*. Statistical Analysis Report. Data Series DR-NSOPF-87/88-I.22. Washington, DC: US Department of Education, Office of Educational Research & Improvement.  US Department of Education, National Center for Educational Statistics. 1991. *National Survey of Postsecondary Faculty (NSOPF:88): Institutional Survey Data File, 1988*. Washington, DC: US Department of Education, Office of Educational Research & Improvement. |
| 1989 | Carnegie Survey of Faculty at US Colleges & Universities^1^ | general purpose survey (with emphasis on political topics) | Carnegie Foundation for the Advancement of Teaching. 1989. *The Condition of the Professoriate: Attitudes & Trends, 1989*. Princeton, NJ: Princeton University Press.  Boyer, Ernest L. 1990. *Scholarship Reconsidered: Priorities of the Professoriate*. Princeton, NJ: Carnegie Foundation for the Advancement of Teaching. |
| 1989-1990 | Higher Education Research Institute (HERI) Faculty Survey^3^ | general purpose survey | Astin, Alexander W., William S. Korn, & Eric L. Dey. 1991. *The American College Teacher: National Norms for the 1989-1990 HERI Faculty Survey*. Higher Education Research Institute. Los Angeles, CA: University of California, Los Angeles. |
| 1991 | *survey of US doctoral students & faculty* | academic misconduct & dishonesty & ethical problems | Swazey, Judith P., Melissa S. Anderson, & Karen Seashore Lewis. 1993. “Ethical Problems in Academic Research.” *American Scientist* 81:542-553.  Anderson, Melissa S., Karen Seashore Lewis, & Jason Earle. 1994. “Disciplinary & Departmental Effects on Observations of Faculty & Graduate Student Misconduct.” *The Journal of Higher Education* 65:331-350.  Louis, Karen Seashore, Melissa S. Anderson, & Lenn Rosenberg. 1995. “Academic Misconduct & Values: The Department's Influence.” *The Review of Higher Education* 18:393-422.  Anderson, Melissa S. 2000. “Normative Orientations of University Faculty & Doctoral Students.” *Science & Engineering Ethics* 6:443-461. |

| **Year(s)** | **Name**  (informal in italics) | **Topic(s)** | **Key Reference(s)** |
| --- | --- | --- | --- |
| 1991 | *survey of risk analysts* | beliefs about prevalence of misconduct, its causes, & solutions | Greenberg, Michael, & J. Martell. 1992. “Ethical Dilemmas & Solutions for Risk Assessment Scientists.” *Journal of Exposure Analysis & Environmental Epidemiolog*y 2:381-389.  Goldberg, Laura, & Michael Greenberg. 1993. “Ethical Issues for Industrial Hygienists: Survey Results & Suggestions.” *American Industrial Hygiene Association Journal* 54:127-34.  Greenberg, Michael, & Laura Goldberg. 1994. “Ethical Challenges to Risk Scientists: An Exploratory Analysis of Survey Data.” *Science, Technology, & Human Values* 19:223-241. |
| 1992 | Carnegie Survey of Faculty at US Colleges & Universities^1^ | general purpose survey (with emphasis on political topics) | Carnegie Foundation for the Advancement of Teaching. 1992. *National Survey of Faculty, 1992*. Princeton, NJ: Princeton University Press. |
| 1992-1993 | National Survey of Postsecondary Faculty^2^ | general purpose survey | Selfa, Lance A., Natalie Suter, Sharon Myers, Shaun Koch, Robert A. Johnson, Daniel A. Zahs, Brian D. Kuhr, & Sameer Y. Abraham. 1997. *1993 National Study of Postsecondary Faculty (NSOPF:93): Methodology Report* (NCES 97-467). US Department of Education, National Center for Education Statistics. Washington, DC: US Government Printing Office. |
| 1992-1993 | Higher Education Research Institute (HERI) Faculty Survey^3^ | general purpose survey | Dey, Eric L., Claudia E. Ramirez, William S. Korn, & Alexander W. Astin. 1993. *The American College Teacher: National Norms for the 1992-1993 HERI Faculty Survey*. Higher Education Research Institute. Los Angeles, CA: University of California, Los Angeles. |
| 1992-1993 | (Carnegie) International Survey of the Academic Profession)^4^ | general purpose survey | Boyer, Ernest L., Philip G. Altbach, & Mary Jean Whitelaw. 1994. *The Academic Profession: An International Perspective, A Special Report*. Princeton, NJ: Carnegie Foundation for the Advancement of Teaching.  Altbach, Philip G., editor. 1996. *The International Academic Profession: Portraits of Fourteen Countries*. Princeton, NJ: Carnegie Foundation for the Advancement of Teaching.  Poole, Millicent, Laurel Bornholt, & Fiona Summers. 1997. “An International Study of the Gendered Nature of Academic Work: Some Cross-Cultural Explorations.” *Higher Education* 34:373-396.  Poole, Millicent, & Laurel Bornholt. 1998. “Career Development of Academics: Cross-Cultural & Lifespan Factors.” *International Journal of Behavioral Development* 22:103-126.  Welch, Anthony, editor. 2005. *The Professoriate: Profile of a Profession*. Dordrecht: Springer. |
| 1995-1996 | Higher Education Research Institute (HERI) Faculty Survey^3^ | general purpose survey | Sax, Linda J. Alexander W. Astin, Marisol Arredondo, & William S. Korn. 1996. *The American College Teacher: National Norms for the 1995-1996 HERI Faculty Survey*. Higher Education Research Institute. Los Angeles, CA: University of California, Los Angeles. |
| 1997 | Carnegie Survey of Faculty at US Colleges & Universities^1^ | general purpose survey (with emphasis on political topics) | Carnegie Foundation for the Advancement of Teaching. 1997. *National Survey of Faculty, 1997*. Princeton, NJ: Princeton University Press.  Zipp, John F., & Rudy Fenwick. 2006. “Is the Academy a Liberal Hegemony?: The Political Orientations & Educational Values of Professors.” *International Journal of Public Opinion Quarterly* 70:304-326. |
| 1998-1999 | National Survey of Postsecondary Faculty^2^ | general purpose survey | Abraham, Sameer Y., Darby Miller Steiger, Margrethe Montgomery, Brian D. Kuhr, Roger Tourangeau, Bob Montgomery, & Manas Chattopadhyay. 2002. *1999 National Study of Postsecondary Faculty (NSOPF:99) Methodology Report* (NCES 2002-154). U.S. Department of Education, National Center for Education Statistics. Washington, DC: US Government Printing Office. |
| 1998-1999 | Higher Education Research Institute (HERI) Faculty Survey^3^ | general purpose survey | Sax, Linda, J., Alexander W. Astin, William S. Korn, & Shannon K. Gilmartin. 1999. *The American College Teacher: National Norms for the 1998-1999 HERI Faculty Survey*. Higher Education Research Institute. Los Angeles, CA: University of California, Los Angeles. |

| **Year(s)** | **Name**  (informal in italics) | **Topic(s)** | **Key Reference(s)** |
| --- | --- | --- | --- |
| 1999 | North American Academic Study Survey | general purpose survey (with emphasis on political topics) | Rothman, Stanley S., Robert Lichter, & Neil Nevitte. 2005. “Politics & Professional Advancement among College Faculty.” *The Forum* 3(1):article 2. |
| 2000 | National Survey on Government Endeavors | political attitudes & behaviors | Light, Paul C. 2000. “Government's Greatest Achievements of the Past Half Century.” *Reform Watch* November 2:1-16.  Brookings Institution. 2001. *National Survey on Government Endeavors*. Prepared by Princeton Survey Research Associates. November. Washington, DC: Brookings Institution. |
| 2000 | Survey of the Academic Profession in Canada | political attitudes & behaviors | Nakhaie, M. Reza. 2001. *The Academic Profession in Canada*. Department of Sociology. University of Windsor.  Nakhaie, M. Reza. 2007. “Universalism, Ascription, & Academic Rank: Canadian Professors, 1987-2000.” *Canadian Review of Sociology & Anthropology* 44(3):361-386.  Nakhaie, M. Reza, & B. Adam. 2008. “Political Affiliations of Canadian University Professors.” *Canadian Journal of Sociology* 33:873-897.  Nakhaie, M. Reza, & Robert J. Brym. 2011. “The Ideological Orientations of Canadian University Professors.” *Canadian Journal of Higher Education* 41:18-33. |
| 2001-2002 | Higher Education Research Institute (HERI) Faculty Survey^3^ | general purpose survey | Lindholm, Jennifer A., Alexander W. Astin, Linda J. Sax, & William S. Korn. 2002. *The American College Teacher: National Norms for the 2001-2002 HERI Faculty Survey*. Higher Education Research Institute. Los Angeles, CA: University of California, Los Angeles. |
| 2002 | *survey of US scientists with NIH funding* | scientific norms, questionable research practices, & scientific integrity | Martinson, Brian C., Melissa S. Anderson, & Raymond De Vries. 2005. “Scientists Behaving Badly.” *Nature* 435:737-738.  Martinson, Brian C., Melissa S. Anderson, A. Lauren Crain, & Raymond De Vries. 2006. “Scientists’ Perceptions of Organizational Justice & Self-Reported Misbehaviors.” *Journal of Empirical Research on Human Research Ethics* 1(1):51-66.  Anderson, Melissa S., Brian C. Martinson, & Raymond De Vries. 2007. “Normative Dissonance in Science: Results from a National Survey of U.S. Scientists.” *Journal of Empirical Research on Human Research Ethics* 2(4):3-14.  Anderson, Melissa S., Emily A. Ronning, Raymond De Vries, & Brian C. Martinson. 2010. “Extending the Mertonian Norms: Scientists’ Subscription to Norms of Research.” *Journal of Higher Education* 81:366-393. |
| 2003 | *survey of members of six scholarly associations* | political attitudes & behaviors | Klein, Daniel B., & Charlotta Stern. 2005. “Professors & Their Politics: The Policy Views of Social Scientists.” *Critical Review* 17(3-4):257-303. |
| 2003-2004 | *survey of biotechnology scientists* | proprietary nature of industry-funded science | Glenna, Leland L., Rick Welsh, David Ervin, William B. Lacy, & Dina Biscotti. 2011. “Commercial Science, Scientists’ Values, & University Biotechnology Research Agendas.” *Research Policy* 40:957-968. |
| 2003-2004 | National Survey of Postsecondary Faculty^2^ | general purpose survey | Cataldi, Emily Forrest, Mansour Fahimi, & Ellen M. Bradburn. 2005. *2004 National Study of Postsecondary Faculty (NSOPF:04) Report on Faculty & Instructional Staff in Fall 2003* (NCES 2005-172). US Department of Education. Washington, DC: National Center for Education Statistics.  Huer, Ruth, Brian Kuhr, Mansour Fahimi, T.R. Curtin, Marjorie Hinsdale, Lisa Carley-Baxter, & Pat Green. 2006. *2004 National Study of Postsecondary Faculty (NSOPF:04): Methodology Report* (NCES 2006-179). US Department of Education, National Center for Education Statistics. Washington, DC: U.S. Government Printing Office. |
| 2004 | *survey of Croatian scientists* | perceptions of science | Prpić, Katarina. 2011. “Science, The Public, & Social Elites: How the General Public, Scientists, Top Politicians, & Managers Perceive Science.” *Public Understanding of Science* 20:733-750. |
| 2004-2005 | *Higher Education Research Institute (HERI) Faculty Survey* | general purpose survey | Lindholm, Jennifer A., Katalin Szelényi, Silvia Hurtado, & William S. Korn. 2005. *The American College Teacher: National Norms for the 2004-2005 HERI Faculty Survey*. Higher Education Research Institute. Los Angeles, CA: University of California, Los Angeles. |
| 2005 | *profile of American college faculty* | political attitudes & behaviors | Tobin, Gary A., & Aryeh K. Weinberg. 2006. *A Profile of American College Faculty: Volume I: Political Beliefs & Behavior*. San Francisco: The Institute for Jewish & Community Research. |
| **Year(s)** | **Name**  (informal in italics) | **Topic(s)** | **Key Reference(s)** |
| 2005 | Factors Affecting Science Communication Survey | science communication | The Royal Society. 2006. *Science Communication: Survey of Factors Affecting Science Communication by Scientists & Engineers*. London: The Royal Society.  Besley, John C., Sang Hwa Oh, & Matthew Nisbet. 2013. “Predicting Scientists’ Participation in Public Life.” *Public Understanding of Science* 22:971-987. |
| 2005 | Survey of US FWS Scientists | political interference & scientific integrity | Union of Concerned Scientists & Public Employees for Environmental Responsibility. 2005. *US Fish & Wildlife Service Scientists Survey Summary*. Cambridge, MA: Union of Concerned Scientists. |
| 2005-2006 | *survey of Swedish social scientists* | political attitudes & behaviors | Berggren, Niclas, Henrik Jordahl, & Charlotta Stern. 2007. “The Political Opinions of Swedish Social Scientists.” IFN Working Paper #711. Stockholm: Research Institute of Industrial Economics (IFN).  Berggren, Niclas, Henrik Jordahl, & Charlotta Stern. 2009. “The Political Opinions of Swedish Social Scientists.” *Finnish Economic Papers* 22(2): 75-88. |
| 2006 | Politics of the American Professoriate Survey | general purpose survey | Gross, Neil, & Solon Simmons. 2007. “The Social & Political Views of American Professors.” Working Paper presented at a Harvard University Symposium on Professors & Their Politics. Cambridge, MA: Harvard University.  Simmons, Solon. 2008. “Ascriptive Justice: The Prevalence, Distribution, & Consequences of Political Correctness in the Academy.” *The Forum* 6(2):0000102202154088841248.  Gross, Neil, & Solon Simmons. 2014. “The Social & Political Views of American College & University Professors.” Pp. 19-51 in *Professors & Their Politics*, edited by Neil Gross & Solon Simmons. Baltimore: Johns Hopkins University Press. |
| 2006 | Survey of US FDA Scientists | political interference & scientific integrity | Union of Concerned Scientists & Public Employees for Environmental Responsibility. 2006. *Voices of Scientists at the FDA*. Cambridge, MA: Union of Concerned Scientists & Public Employees for Environmental Responsibility. |
| 2006 | Survey of Federal Climate Scientists | political interference & scientific integrity | Donaghy, Timothy, Jennifer Freeman, Francesco Grifo, Karly Kaufman, Tarek Maassarani, & Lexi Shultz. 2007. *Atmosphere of Pressure: Political Interference in Federal Climate Science*. Cambridge, MA: Union of Concerned Scientists & Government Accountability Project. |
| 2006 | Ithaka S+R Faculty Survey^5^ | general purpose survey | Housewright, Ross, & Roger Schonfeld. 2008. *Ithaka’s 2006 Studies of Key Stakeholders in the Digital Transformation in Higher Education*. New York: Ithaka S+R. |
| 2007 | Survey of EPA Scientists | political interference & scientific integrity | Donaghy, Timothy, Francesca Grifo, & Meredith McCarthy. 2008. *Interference at the EPA: Science & Politics at the US Environmental Protection Agency*. Cambridge, MA: Union of Concerned Scientists. |
| 2007-2008 | Higher Education Research Institute (HERI) Faculty Survey^3^ | general purpose survey | DeAngelo, Linda, Silvia Hurtado, John H. Pryor, Kimberly R. Kelly, José Luis Santos, & William S. Korn. 2009. *The American College Teacher: National Norms for the 2007-2008 HERI Faculty Survey*. Higher Education Research Institute. Los Angeles, CA: University of California, Los Angeles. |
| 2007-2008 | Changing Academic Profession Survey^4^ | general purpose survey | Research Institute for Higher Education. 2009. *The Changing Academic Profession over 1992-2007: International, Comparative, & Quantitative Perspectives*. RIHE Internation Seminar Report #13. Hiroshima: Hiroshima University.  Cummings, William K., & Martin J. Finkelstein. 2012. *Scholars in the Changing American Academy: New Contexts, New Rules, & New Roles*. Dordecht: Springer.  Bentley, Peter James, Hamish Coates, Ian R. Dobson, Leo Goedegebuure, & V. Lynn Meek, editors. 2013. *Job Satisfaction around the Academic World*. Dordecht: Springer.  Kehm, Barbara M., & Ulrich Teichler, editors. 2013. *The Academic Profession in Europe: New Tasks & New Challenges*. Dordrecht: Springer.  Teichler, Ulrich, Akira Arimoto, & William K. Cummings. 2013. *The Changing Academic Profession: Major Findings of a Comparative Survey*. Dordrecht: Springer.  Huang, Futao, Martin Finkelstein, & Michele Rostan, editors. 2014. *The Internationalization of the Academy: Changes, Realities, & Prospects*. Dordecht: Springer.  Cummings, William K., & Ulrich Teichler, editors. 2015. *The Relevance of Academic Work in Comparative Perspective*. Dordecht: Springer. |
| **Year(s)** | **Name**  (informal in italics) | **Topic(s)** | **Key Reference(s)** |
| 2009 | Ithaka S+R Faculty Survey^5^ | general purpose survey | Schonfeld, Roger C., & Ross Housewright. 2010. *Faculty Survey 2009: Key Strategic Insights for Libraries, Publishers, & Societies*. New York: Ithaka S+R. |
| 2009 | Survey of Organizational Research Climate | research integrity | Wells, James A., Carol R. Thrush, Brian C. Martinson, Terry A. May, Michelle Stickler, Eileen C. Callahan, & Karen L. Klomparens. 2014. “Survey of Organizational Research Climates in Three Research Intensive, Doctoral Granting Universities.” *Journal of Empirical Research on Human Research Ethics* 9(5):72-88. |
| 2009 | Pew Survey of AAAS Members^6^ | general purpose survey | Pew Research Center. 2009. *Scientific Achievements Less Prominent Than a Decade Ago: Public Praises Science; Scientists Fault Public, Media*. July 2009 Report. Washington, DC: Pew Research Center.  Besley, John C., Sang Hwa Oh, & Matthew Nisbet. 2013. “Predicting Scientists’ Participation in Public Life.” *Public Understanding of Science* 22:971-987. |
| 2009-2010 | Japan Knowledge Creation Survey^7^ | knowledge creation process | Nagaoka, Sadao, Masatsura Igami, Manabu Eto, & Tomohiro Ijichi. 2010. *Knowledge Creation Process in Science: Basic Findings from a Large-Scale Survey of Researchers in Japan*. December 2010 Report. Tokyo: Hitotsubashi University Institute of Innovation Research.  Lee, You-Na, John P. Walsh, & Jian Wang. 2015. “Creativity in Scientific Teams: Unpacking Novelty & Impact.” *Research Policy* 44:684-697.  Murayama, Kota, Makoto Nirei, & Hiroshi Shimizu. 2015. “Management of Science, Serendipity, & Research Performance: Evidence from a Survey of Scientists in Japan & the U.S.” *Research Policy* 44:862-873.  Matsumoto, Kuniko, Sotaro Shibayama, Byeongwoo Kang, & Masatsura Igami. 2021. “Introducing a Novelty Indicator for Scientific Research: Validating the Knowledge‑Based Combinatorial Approach.” *Scientometrics* 126:6891-6915. |
| 2010 | EuroAC Survey^4^ | general purpose survey | Kehm, Barbara M., & Ulrich Teichler, editors. 2013. *The Academic Profession in Europe: New Tasks & New Challenges*. Dordrecht: Springer.  Teichler, Ulrich, Akira Arimoto, & William K. Cummings. 2013. *The Changing Academic Profession: Major Findings of a Comparative Survey*. Dordrecht: Springer.  Teichler, Ulrich, & Ester Ava Höhle, editors. 2013. *The Work Situation of the Academic Profession in Europe: Findings of a Survey in Twelve Countries*. Dordrecht: Springer.  Fumasoli, Tatiana, Gaële Goastellec, & Barbara M. Kehm, editors. 2015. *Academic Work & Careers in Europe: Trends, Challenges, Perspectives*. Dordecht: Springer.  Machado-Taylor, Maria de Lourdes, Virgilio Meira Soares, & Ulrich Teichler, editors. 2017. *Challenges & Options: The Academic Profession in Europe*. Dordrecht: Springer. |
| 2010 | Survey of Federal Food Safety Scientists | political interference & scientific integrity | Donaghy, Timothy, Francesca Grifo, Michael Halpern, & Heidi Moline. 2010. *Driving the Fox from the Henhouse: Improving Oversight of Food Safety at the FDA & USDA*. Cambridge, MA: Union of Concerned Scientists. |
| 2010-2011 | Higher Education Research Institute (HERI) Faculty Survey^3^ | general purpose survey | Hurtado, Silvia Kevin Eagan, John H. Pryor, Hannah Whang, & Serge Tran. 2012. *Undergraduate Teaching Faculty: The 2010-2011 HERI Faculty Survey*. Higher Education Research Institute. Los Angeles, CA: University of California, Los Angeles. |
| 2010-2011 | US Knowledge Creation Survey^7^ | knowledge creation process | Nagaoka, Sadao, Masatsura Igami, John P. Walsh, & Tomohiro Ijichi. 2011. *Knowledge Creation Process in Science: Key Comparative Findings from the Hitotsubashi-NISTEP-Georgia Tech Scientists’ Survey in Japan & the US.* October 2011 Report. Tokyo: Hitotsubashi University Institute of Innovation Research.  Murayama, Kota, Makoto Nirei, & Hiroshi Shimizu. 2015. “Management of Science, Serendipity, & Research Performance: Evidence from a Survey of Scientists in Japan & the U.S.” *Research Policy* 44:862-873.  Lee, You-Na, John P. Walsh, & Jian Wang. 2015. “Creativity in Scientific Teams: Unpacking Novelty & Impact.” *Research Policy* 44:684-697.  Matsumoto, Kuniko, Sotaro Shibayama, Byeongwoo Kang, & Masatsura Igami. 2021. “Introducing a Novelty Indicator for Scientific Research: Validating the Knowledge‑Based Combinatorial Approach.” *Scientometrics* 126:6891-6915. |

| **Year(s)** | **Name**  (informal in italics) | **Topic(s)** | **Key Reference(s)** |
| --- | --- | --- | --- |
| 2010-2014 | *survey of active US researchers* | authorship & citation manipulation | Fong, Eric A., & Allen W. Wilhite. 2017. “Authorship & Citation Manipulation in Academic Research.” *PLoS ONE* 12(2):e0187394. |
| 2011 | *survey of Midwestern faculty* | support for Mertonian norms of science | Motycka, Eric D. 2011. *Development of an Instrument to Measure Faculty Adherence to the Norms of Science*. Doctoral Dissertation. Department of Educational Leadership. Terre Haute, IN: Indiana State University. |
| 2011 | Survey of Food & Drug Administration Scientists | political interference & scientific integrity | Union of Concerned Scientists. 2012. *Voices of Scientists at the FDA: Measuring Progress on Scientific Integrity*. Cambridge, MA: Union of Concerned Scientists. |
| 2012 | Ithaka S+R Faculty Survey^5^ | general purpose survey | Housewright, Ross, Roger C. Schonfeld, & Kate Wulfson. 2013. *Ithaka S+R US Faculty Survey 2012*. New York: Ithaka S+R. |
| 2012 | *survey of AAAS members* | science communication training | Besley, John C., Anthony Dudo, & Martin Storksdieck. 2015. “Scientists Views’ about Communication Training.” *Journal of Research in Science Teaching* 52:199-220. |
| 2012 | *survey of scientists’ views about Protestant Christians* | views about different religious groups | Yancey, George, Sam Reiner, & Jake O’Connell. 2015. “How Academics View Conservative Protestants.” *Sociology of Religion* 76:315-336. |
| 2012 | Ithaka S+R, Jisc, & RLUK Survey of UK Academics^8^ | general purpose survey | Housewright, Ross, Roger C. Schonfeld, & Kate Wulfson. 2013. *Ithaka S+R, Jisc, RLUK UK Survey of Academics 2012*. New York: ITHAKA. |
| 2012-2013 | Academic Profession in Asia Survey^4^ | general purpose survey | Research Institute for Higher Education. 2013. *The Changing Academic Profession in Asia: Teaching, Research, Government, & Management*. RIHE Internation Seminar Report #20. Hiroshima: Hiroshima University.  Research Institute for Higher Education. 2015. *The Changing Academic Profession in Asia: The Formation, Work, Academic Productivity, & Internationalization of the Academy*. RIHE Internation Seminar Report #22. Hiroshima: Hiroshima University.  Huang, Futao. 2021. “Challenges to the Asian Academic Profession: Major Findings from the International Surveys.” *Higher Education Quarterly* 75:438-452.  Huang, Futao, & Anthony R. Welch, editors. 2021. *International Faculty in Asia*. Dordrecht: Springer. |
| 2013 | *survey of AAAS members* | scientific communication & public engagement | Dudo, Anthony, & John C. Besley. 2016. “Scientists’ Prioritization of Communication Objectives for Public Engagement.” *PLoS ONE* 11(2):e0148867. |
| 2013 | *EUROAC Survey of Slovenia* | general purpose survey | Manja Klemenčič, Manja, Alenka Flander, & Mateja Žagar Pečjak. 2015. *Academic Profession & the Conditions of Academic Work in Slovenia: Findings of the 2013 EUROAC Survey*. Ljubljana, Slovenia: Centre of the Republic of Slovenia for Mobility & European Educational & Training Programmes (CMEPIUS). |
| 2013 | *survey of academics at a large research university* | data-sharing practices | Defazio, Daniela, Christos Kolympiris, Markus Perkmann, & Ammon Salter. 2022. “Busy Academics Share Less: The Impact of Professional & Family Roles on Academic Withholding Behavior.” *Studies in Higher Education* 47:731-750. |
| 2013-2014 | Higher Education Research Institute (HERI) Faculty Survey^3^ | general purpose survey | Eagan, Kevin, Ellen Bara Stolzenberg, Jennifer Berdan Lozano, Melissa C. Aragon, Maria Ramirez Suchard, & Sylvia Hurtado. 2014. *Undergraduate Teaching Faculty: The 2013-2014 HERI Faculty Survey*. Higher Education Research Institute. Los Angeles, CA: University of California, Los Angeles. |
| 2014 | Pew Survey of AAAS Members^6^ | general purpose survey | Pew Research Center. 2015a. *An Elaboration of AAAS Scientists’ Views*. July 2015 Report. Washington, DC: Pew Research Center.  Pew Research Center. 2015b. *How Scientists Engage the Public*. February 2015 Report. Washington, DC: Pew Research Center. |
| **Year(s)** | **Name**  (informal in italics) | **Topic(s)** | **Key Reference(s)** |
| 2015 | Ithaka S+R Faculty Survey^5^ | general purpose survey | Wolff-Eisenberg, Christine, Alisa B. Rod, & Roger C. Schonfeld. 2016. *Ithaka S+R US Faculty Survey 2015*. New York: Ithaka S+R. |
| 2015 | Ithaka S+R, Jisc, & RLUK Survey of UK Academics^8^ | general purpose survey | Housewright, Ross, Roger C. Schonfeld, & Kate Wulfson. 2016. *Ithaka S+R, Jisc, RLUK UK Survey of Academics 2015*. New York: ITHAKA. |
| 2015 | *survey of social science journal article authors* | strategic research agendas | Santos, João M., Hugo Horta, & Huan Li. 2022. “Are the Strategic Research Agendas of Researchers in the Social Sciences Determinants of Research Productivity?” *Scientometrics* 127:3719-3747. |
| 2015 | Survey of Government Scientists | political interference & scientific integrity | Goldman, Gretchen, Michael Halpern, Deborah Bailin, Arnold Olali, Charise Johnson, & Tim Donaghy. 2015. *Progress & Problems: Government Scientists Report on Scientific Integrity at Four Agencies*. Cambridge, MA: Union of Concerned Scientists. |
| 2015-2016 | *surveys of professional societies' memberships* | public engagement activities | Besley, John C., Anthony Dudo, & Shupei Yuan. 2018. “Scientists' Views about Communication Objectives.” *Public Understanding of Science* 27:708-730.  Besley, John C., Anthony Dudo, Shupei Yuan, & Frank Lawrence. 2018. “Understanding Scientists’ Willingness to Engage.” *Science Communication* 40:559-590.  Davis, Cathlyn, Caitlin Weber, & Nalini M. Nadkarni. 2022. “Prevalence of Discourse on Public Engagement with Science in Ecology Literature.” *Frontiers in Ecology & the Environment* 20:524-530. |
| 2016 | *survey of Web of Science authors* | views about authorship | Smith, Elise, Bryn Williams-Jones, Zubin Master, Vincent Larivière, Cassidy R. Sugimoto, Adèle Paul-Hus, Min Shi, & David B. Resnik. 2020. “Misconduct & Misbehavior Related to Authorship Disagreements in Collaborative Science.” *Science & Engineering Ethics* 26:1967-1993. |
| 2016-2017 | Higher Education Research Institute (HERI) Faculty Survey^3^ | general purpose survey | Stolzenberg, Ellen Bara, Kevin Eagan, Hilary B. Zimmerman, Jennifer Berdan Lozano, Natacha M. Cesar-Davis, Melissa C. Aragon, & Cecilia Rios-Aguilar. 2019. *Undergraduate Teaching Faculty: The 2016-2017 HERI Faculty Survey*. Higher Education Research Institute. Los Angeles, CA: University of California, Los Angeles. |
| 2016-2017 | *survey of tenure-system faculty in US research universities* | perception of research fraud | Pratt, Travis C., Michael D. Reisig, Kristy Holtfreter, & Golladay. 2019. “Scholars’ Preferred Solutions for Research Misconduct: Results from a Survey of Faculty Members at America’s Top 100 Research Universities.” *Ethics & Behavior* 29(7):510-530.  Holtfreter, Kristy, Michael D. Reisig, Travis C. Pratt, & Ryan D. Mays. 2020. “The Perceived Causes of Research Misconduct among Faculty Members in the Natural, Social, & Applied Sciences.” *Studies in Higher Education* 45:2162-2174.  Reisig, Michael D., Kristy Holtfreter, & Marcus E. Berzofsky. 2020. “Assessing the Perceived Prevalence of Research Fraud among Faculty at Research-Intensive Universities in the USA.” *Accountability in Research* 27:457-475. |
| 2016-2017 | *survey on scientific realism* | views about scientific realism | Beebe, James R. & Finnur Dellsén. 2020. “Scientific Realism in the Wild: An Empirical Study of Seven Sciences & History & Philosophy of Science.” *Philosophy of Science* 87:1-29.  Beebe, James R., & Finnur Dellsén. 2025. “The Centrality of Progressive Realism to the Scientific Realism Debate.” *Philosophy of Science* 92(3):1-31. doi:10.1017/psa.2025.10092 |
| 2017 | *survey of ecology & evolution biology journal corresponding authors* | questionable research practices | Fraser, Hannah, Tim Parker, Shinichi Nakagawa, Ashley Barnett, & Fiona Fidler. 2018. “Questionable Research Practices in Ecology & Evolution.” *PLoS ONE* 13:e0200303. |

| **Year(s)** | **Name**  (informal in italics) | **Topic(s)** | **Key Reference(s)** |
| --- | --- | --- | --- |
| 2017 | *survey on research integrity in Amsterdam* | perceptions of research integrity | Haven, Tamarinde L., Joeri K. Tijdink, Brian C. Martinson, & Lex M. Bouter. 2019. “Perceptions of Research Integrity Climate Differ Between Academic Ranks & Disciplinary Fields: Results from a Survey among Academic Researchers in Amsterdam.” *PLoS ONE* 14:e0210599.  Haven, Tamarinde L., Joeri K. Tijdink, Brian C. Martinson, Lex M. Bouter, & Frans Oort. 2021. “Explaining Variance in Perceived Research Misbehaviors: Results from a Survey among Academic Researchers in Amsterdam.” *Research Integrity & Peer Review* 6:7. |
| 2017 | *survey of Spanish faculty* | international research collaboration | de Frutos‑Belizón, Jesús, Natalia García‑Carbonell, Marta Ruíz-Martínez, & Gonzalo Sánchez‑Gardey. 2023. “Disentangling International Research Collaboration in the Spanish Academic Context: Is There A Desirable Researcher Human Capital Profile?” *Research Policy* 52:104779.  de Frutos‑Belizón, Jesús, Natalia García‑Carbonell, Félix Guerrero‑Alba, & Gonzalo Sánchez‑Gardey. 2024. “An Empirical Analysis of Individual & Collective Determinants of International Research Collaboration.” *Scientometrics* 129:2749-2770. |
| 2017-2018 | *survey of NSERC grant recipients* | views about public engagement | Besley, John C., Todd P. Newman, Anthony Dudo, & Leigh Anne Tiffany. 2020. “Exploring Scholars’ Public Engagement Goals in Canada & the United States.” *Public Understanding of Science* 29:855-867. |
| 2017-2018 | Academic Profession in the Knowledge-Based Society (APIKS) Survey^4^ | general purpose survey | Postiglione, Gerard A., & Yangson Kim. 2020. “The Research & Teaching Nexus of Academics in the New Era: Findings from the Academic Profession in a Knowledge-Based Society (APIKS).” *Higher Education Forum* 17(3):21-24.  Aarrevaara, Timo, Martin Finkelstein, Glen A. Jones, & Jisun Jung, editors. 2021. *Universities in the Knowledge Society: The Nexus of National Systems of Innovation & Higher Education*. Dordrecht: Springer.  Huang, Futao, Timo Aarrevaara, & Ulrich Teicher, editors. 2022. *Teaching & Research in the Knowledge-Based Society*. Dordrecht: Springer.  Çalıkoğlu, Alper, Glen A. Jones, Yangson Kim, editors. 2023. *Internationalization & the Academic Profession: Comparative Perspectives*. Dordrecht: Springer. |
| 2018 | Ithaka S+R Faculty Survey^5^ | general purpose survey | Blankstein, Melissa, & Christine Wolff-Eisenberg. 2019. *Ithaka S+R US Faculty Survey 2018*. New York: Ithaka S+R. |
| 2018 | Survey on Research Integrity in Norway | questionable research practices | Kaiser, Matthias, Laura Drivdal, Johs Hjellbrekke, Helene Ingierd, & Ole Bjørn Rekdal. 2022. “Questionable Research Practices & Misconduct among Norwegian Researchers.” *Science & Engineering Ethics* 28:2. doi:10.1007/s11948-021-00351-4. |
| 2018 | *survey of scientists at AAU institutions* | views about public engagement | Copple, Jacob, Nichole Bennett, Anthony Dudo, Won-Ki Moon, Todd P. Newman, John Besley, Nicole Leavey, Laura Lindefeld, & Chris Volpe. 2020. “Contribution of Training to Scientists’ Public Engagement Intentions: A Test of Indirect Relationships Using Parallel Multiple Mediation.” *Science Communication* 42:508-537.  Besley, John C., Todd P. Newman, Anthony Dudo, & Leigh Anne Tiffany. 2020. “Exploring Scholars’ Public Engagement Goals in Canada & the United States.” *Public Understanding of Science* 29:855-867. |
| 2018 | *survey of land-grant university faculty* | public scholarship | Rose, Kathleen, Ezra M. Markowitz, & Dominique Brossard. 2020. “Scientists’ Incentives & Attitudes toward Public Communication.” *Proceedings of the National Academy of Sciences* 117:1274-1276.  Bao, Luye, Mikhaila N. Calice, Dominique Brossard, Becca Beets, Dietram A. Scheufele, & Kathleen M. Rose. 2023. “How Institutional Factors at US Land-Grant Universities Impact Scientists’ Public Scholarship.” *Public Understanding of Science* 32:124-142.  Bao, Luye, Mikhaila N. Calice, Dominique Brossard, Dietram A. Scheufele, & Ezra M. Markowitz. 2024. “Are Productive Scientists More Willing to Engage with the Public?” *Science Communication* 46:65-91. |
| 2018 | *survey of US federal scientists* | perceptions of scientific integrity | Carter, Jacob, Gretchen Goldman, & Charise Johnson. 2018. *Science under Trump: Voices of Scientists across 16 Federal Agencies*. Cambridge, MA: Union of Concerned Scientists.  Goldman, Gretchen T., Jacob M. Carter, Yun Wang, & Janice M. Larson. 2020. “Perceived Losses of Scientific Integrity under the Trump Administration: A Survey of Federal Scientists.” *PLoS ONE* 15:e0231929. |
| **Year(s)** | **Name**  (informal in italics) | **Topic(s)** | **Key Reference(s)** |
| 2018 | *survey of US & Canadian university scientists* | views about faculty evaluation | Niles, Meredith T., Lesley A. Schimanski, Erin C. McKiernan, Juan Pablo Alperin. 2020. “Why We Publish Where We Do: Faculty Publishing Values and Their Relationship to Review, Promotion, and Tenure Expectations.” *PLoS ONE* 15(3):e0228914. |
| 2018 | *survey of Swedish researchers* | views about decision to cancel Elsevier subscriptions | Olsson, Lisa, Camilla Lindelöw, Lovisa Österlund, and Frida Jakobsson. 2020, “Swedish Researchers’ Responses to the Cancellation of the Big Deal with Elsevier.” *Insights* 33(25):1-23. |
| 2018 | *survey on transparency in research* | transparency in research | Malički, Mario, IJsbrand Jan Aalbersberg, Lex Bouter, Adrian Mulligan, and Gerben ter Riet. 2023. “Transparency in Conducting and Reporting Research: A Survey of Authors, Reviewers, and Editors across Scholarly Disciplines.” *PLoS ONE* 18(3):e0270054. |
| 2018, 2020 | *survey of social science faculty & PhD students* | open science attitudes & practices | Christensen, Garret, Zenan Wang, Elizabeth Levy Paluck, Nicholas Swanson, David Birke, Edward Miguel, & Rebecca Littman. 2020. “Open Science Practices Are on the Rise: The State of the Social Science (3S) Survey.” Working Papers Series #WPS-106. Center for Effective Global Action. Berkeley: University of California.  Ferguson, Joel, Rebecca Littman, Garret Christensen, Elizabeth Levy Paluck, Nicholas Swanson, Zenan Wang, Edward Miguel, David Birke, & John-Henry Pezzuto. 2023. “Survey of Open Science Practices & Attitudes in the Social Sciences.” *Nature Communications* 14:5401. |
| 2019 | Finnish Research Integrity Barometer | research integrity | Salminen, Ari, & Lotta Pitkänen. 2020. *Finnish Research Integrity Barometer 2018*. Helsinki: Finnish National Board on Research Integrity. |
| 2019 | *concept of knowledge in 'Gettier' situations surveys* | concept of knowledge in "Gettier" situations | Starmans, Christina, & Ori Friedman. 2020. “Expert or Esoteric?: Philosophers Attribute Knowledge Differently Than All Other Academics.” *Cognitive Science* 44:e12850. |
| 2019 | Credibility of Preprints Survey | views about article preprints | Soderberg, Courtney K., Timothy M. Errington, & Brian A. Nosek. 2020. “Credibility of Preprints: An Interdisciplinary Survey of Researchers.” *Royal Society Open Science* 7:201520. |
| 2019-2020 | Higher Education Research Institute (HERI) Faculty Survey^3^ | general purpose survey | report for the 2019-2020 HERI faculty survey not available |
| 2019-2020 | *survey of climate change article authors* | views about public engagement | Cologna, Viktoria, Reto Knutti, Naomi Oreskes, & Michael Siegrist. 2021. “Majority of German Citizens, US Citizens, & Climate Scientists Support Policy Advocacy by Climate Researchers & Expect Greater Political Engagement.” *Environmental Research Letters* 16:024011. |
| 2019-2020 | *survey of early- & mid-career faculty* | faculty-to-faculty mentorship | Sarabipour, Sarvenaz, Natalie M. Niemi, Steven J. Burgess, Christopher T. Smith, Alexandre W. Bisson Filho, Ahmed Ibrahim, & Kelly Clark. 2023. “The Faculty-to-Faculty Mentorship Experience: A Survey on Challenges & Recommendations for Improvements.” *Proceedings of the Royal Society B* 290:20230983. |
| 2020 | *survey of academic researchers in The Netherlands* | questionable research practices | Gopalakrishna, Gowri, Gerben ter Riet, Gerko Vink, Ineke Stoop, Jelte M. Wicherts, & Lex M. Bouter. 2022. “Prevalence of Questionable Research Practices, Research Misconduct, & Their Potential Explanatory Factors: A Survey among Academic Researchers in The Netherlands.” *PLoS ONE* 17:e0263023. |
| 2020 | *survey of university teachers in Tuscany* | experiences during first COVID-19 lockdown | Parlangeli, Oronzo, Paola Palmitesta, Margherita Bracci, Enrica Marchigiani, Ileana Di Pomponio, & Stefano Guidi. 2022. “University Teachers during the First Lockdown due to SARS‑CoV‑2 in Italy: Stress, Issues, & Perceptions of Misconduct.” *Science & Engineering Ethics* 28:9. |
| 2020 | Responsible Research Barometer in Lithuania | research integrity | Ozolinčiūtė, Eglė, Gintarė Židonė, & Loreta Tauginienė. 2020. *Responsible Research Barometer 2020*. Vilnius: Office of the Ombudsperson for Academic Ethics & Procedures of the Republic of Lithuania. |
| 2021 | Ithaka S+R Faculty Survey5 | general purpose survey | Blankstein, Melissa. 2022. Ithaka S+R US Faculty Survey 2021 Report. New York: Ithaka S+R. |
| **Year(s)** | **Name**  (informal in italics) | **Topic(s)** | **Key Reference(s)** |
| 2021 | Quantifying & Understanding Stress & Retention in Academia Survey | stress & retention in academia | Spoon, Katie, Nicholas LaBerge, K. Hunter Wapman, Sam Zhang, Allison C. Morgan, Mirta Galesic, Bailey K. Fosdick, Daniel B. Larremore, & Aaron Clauset. 2023. “Gender & Retention Patterns among U.S. Faculty.” *Science Advances* 9:eadi2205. |
| 2021 | *survey of Chinese faculty* | relationship between teaching & research | Li, Tingsong, & Xi Yang. 2024. “Conflict or Facilitation?: Faculty Members’ Teaching Time & Their Research Performance.” *Higher Education* 87:1433-1451. |
| 2021 | *survey of theoretical virtues* | views about scientific practice & theoretical virtues | Schindler, Samuel. 2022. “Theoretical Virtues: Do Scientists Think What Philosophers Think They Ought to Think?” *Philosophy of Science* 89:542-564. |
| 2021-2022 | *survey of research integrity culture* | research integrity & local research culture | De Peuter, Steven, Kris Dierickx, Martin Meganck, Inge Lerouge, Wouter Vandevelde, & Gert Storms. 2024. “Mismatch in Perceptions of the Quality of Supervision & Research Data Management as an Area of Concern: Results from a University-Wide Survey of the Research Integrity Culture at a Belgian University.” *Accountability in Research* February:1-32. doi:10.1080/08989621.2024.2318245. |
| 2022 | *understanding how researchers assess research outputs survey* | how scholars evaluate others’ work | Hrynaszkiewicz, Iain, Beruria Novich, James Harney, & Veronique Kiermer. 2023. “A Survey of How Biology Researchers Assess Credibility when Serving on Grant & Hiring Committees.” 27th International Conference on Science, Technology, & Innovation Indicators (STI 2023). doi:10.55835/642ee2edfab37d565e6081a9. |
| 2022 | *survey of researchers in Sweden* | views about ethical research behavior | Lindkvist, Amanda M., Lina Koppel, & Gustav Tinghӧg. 2024. “Bounded Research Ethicality: Researchers Rate Themselves & Their Field as Better than Others at Following Good Research Practice.” *Scientific Reports* 14:3050. |
| 2022 | *survey of members of international professional societies* | views about environmental & natural resource social science | Ulrich-Schad, Jessica D., John Aloysius Zinda, Hua Qin, David Matarrita-Cascante, Jennifer E. Givens, & Chuntian Lu. 2024. “Unveiling Perspectives & Insights: A Survey of Environmental & Natural Resource Sociologists & Social Scientists.” *Society & Natural Resources* 37:428-441. |
| 2022 | *survey on unpublished data & unused samples* | experiences with unpublished data & unused samples | Bowers, Emma C., Jimena Stephenson, Melissa Furlong, & Kenneth S. Ramos. 2023. “Scope & Financial Impact of Unpublished Data & Unused Samples among US Academic & Government Researchers.” *iScience* 26:107166. |
| 2022 | *survey of Belgian scientists* | questionable research practices | Conix, Stijn, Steven De Peuter, Andreas De Block, & Krist Vaesen. 2023. “Questionable Research Practices in Competitive Grant Funding: A Survey.” *PLoS ONE* 18(11):e0293310. |
| 2022 | *survey of early career researchers* | impact of COVID-19 on early career researchers | Jamali, Hamid R., David Nicholas, David Sims, Anthony Watkinson2 Eti Herman, Cherifa Boukacem-Zeghmouri, Blanca Rodríguez-Bravo, Marzena Świgoń, Abdullah Abrizah, Jie Xu, Carol Tenopir, & Suzie Allard. 2023. “The Pandemic & Changes in Early Career Researchers’ Career Prospects, Research, & Publishing Practices.” *PLoS ONE* 18(2):e0281058. |
| 2022 | Survey of Federal Scientists | political interference & scientific integrity | Desikan, Anita, & Jacob Carter. 2023. *Getting Science Back on Track: Voices of Scientists across Six Federal Agencies*. Cambridge, MA: Union of Concerned Scientists. |
| 2022-2023 | Higher Education Research Institute (HERI) Faculty Survey^3^ | general purpose survey | report for the 2022-2023 HERI faculty survey not available |
| 2022-2023 | *survey of European researchers* | views about research ethics & role of institutional support | Berghaeuser, Hendrik, Max Prass, & Ralf Lindner. 2024. “Researchers’ Practice & Perception of Research Ethics & the Role of Institutional Support: Insights from a Pan-European Researcher Survey.” *Journal of Academic Ethics* doi: 10.1007/s10805-024-09590-z. |

| **Year(s)** | **Name**  (informal in italics) | **Topic(s)** | **Key Reference(s)** |
| --- | --- | --- | --- |
| 2023 | *survey of Thai researchers* | scientific integrity in scholarly publishing | Nguyen, Lan Thi, & Kulthida Tuamsuk. 2024. “Factors Influencing Researchers’ Scientific Integrity in Scholarly Publishing: A Path Analysis Approach.” *Research Ethics* doi: 10.1177/17470161241257445. |
| 2023 | *survey on research integrity in Estonia* | research ethics & research integrity | Simm, Kadri, Kadri Lees, Mari-Liisa Parder, & Anu Tammeleht. 2023. *Development & Piloting of the Methodology for the Estonian Research Ethics Survey: Final Report*. Tartu: University of Tartu.  Simm, Kadri, Mari-Liisa Parder, Anu Tammeleht, & Kadri Lees. 2024. “National Cross-Disciplinary Research Ethics & Integrity Study: Methodology & Results from Estonia.” *Research Ethics* 20:514-538. |
| 2023 | *survey of university professors in Ecuador* | tension, irritability, & fatigue among university professors | Cadena-Povea, Henry, Marco Hernández-Martínez, Gabriela Bastidas-Amador, & Josué Calderón-Muñoz. 2025. “Perceived Stress: Psychosocial-Sociodemographic Factors as Predictors of Tension, Irritability, & Fatigue among Ecuadorian University Professors.” *International Journal of Environmental Health & Public Health* 22:107. |

^1^ The Carnegie Survey of Faculty at US Colleges & Universities had 6 waves between 1969 and 1997.

^2^ The National Survey of Postsecondary Faculty had 4 waves between 1987 and 2004.

^3^ The Higher Education Research Institute (HERI) Faculty Survey had 11 waves between 1989 and 2023.

^4^ The 1992-1993 (Carnegie) International Survey of the Academic Profession spawned four subsequent international survey projects: the 2007-2008 Changing Academic Profession Survey, the 2010 EuroAC Survey, the 2012-2013 Academic Profession in Asia Survey, and the 2017-2018 Academic Profession in the Knowledge-Based Society (APIKS) Survey.

^5^ The Ithaka S+R Faculty Survey had 6 waves between 2006 and 2021.

^6^ The Pew Survey of AAAS Members was administered in 2009 and 2014.

^7^ The Knowledge Creation Survey was administered in Japan in 2009-2010 and in the USA in 2010-2011.

^8^ The Ithaka S+R, Jisc & RLUK Survey of UK Academics was administered in 2012 and 2015.

**Notes**: This nearly comprehensive list excludes those studies/surveys that (a) sampled from only one discipline, (b) sampled almost exclusively from clinical medicine or biomedical science, or (c) had a sample of <250 scientists. For each of the above studies, we recorded the following characteristics (where available):

- survey description: administration year(s), formal or informal survey name, topic(s) covered;
- sampling design: sampled country/countries, target population, sampling frame, contacted potential respondents, sample size, response rate, science families/disciplines included, respondent employment types;
- survey administration: mode(s), dates, number of contacts, types of contacts, median time to completion;
- disciplinary measurement:
  - measured dimensions: PhD, current department, professional society/association, primary targeted journals, peer perception, self-identification;
  - indicator type: closed-ended survey question, open-ended survey question; implied via sampling frame construction (not measured on survey);
  - most disaggregated field level: discipline, family, mix of disciplines and families.

**Extended Review of the 115 Surveys of Scientists, 1955-2023**

We aimed to better understand how researchers have measured scientists’ disciplinary affiliation on surveys over the years. In particular, we intended to find all surveys of scientists for which such measurement was at least modestly consequential (i.e., surveys aiming to differentiate respondents into one of multiple disciplines so scholars could perform comparative analyses of scientists across disciplines). We performed a nearly exhaustive search of English-language publications for all such surveys that met four selection criteria.

1. We included surveys that were administered to an at least moderately sized sample of respondents. We excluded surveys with relatively small samples (N<250), since these were unlikely to include sufficient numbers of respondents from individual disciplines to allow for robust comparative analyses.
2. We included surveys that primarily or exclusively sampled PhD scientists whether or not they were employed in academia. We excluded surveys that mostly or exclusively sampled graduate students (i.e., scientists-in-training).
3. We included surveys administered to respondents from at least two distinct scientific disciplines. We excluded all surveys administered to members of only one discipline.
4. We excluded surveys administered mostly or exclusively to respondents trained in clinical medicine or biomedical sciences. Such respondents—who likely are employed in medical schools or hospitals (and not science colleges and departments)—are outside of the focus of our own study.

As of July 2025, our search identified 115 distinct surveys administered since 1955. **Table A** above lists all of these surveys in chronological order by the date of their initial administration. We counted a survey as distinct when it aligned with one of the following descriptions.

- *a one-time survey*: This is a unique survey administered once to a single sample.
- *multi-national administration of a common survey*: An international leadership team, often with country-specific principal investigators, administered the same (or mostly the same) survey in multiple countries at roughly the same time—usually with similar sampling procedures across nations.
- *a single wave of a longitudinal survey*: A leadership team administered a survey to independent samples of respondents across multiple years (i.e., waves), and the surveys’ measurements instruments (as well as the team’s sampling procedures) tended to change over time.

For each of the 115 surveys above, we recorded the following characteristics (where available):

- survey description: administration year(s), formal or informal survey name, topic(s) covered;
- sampling design: sampled country/countries, target population, sampling frame, contacted potential respondents, sample size, response rate, science families/disciplines included, respondent employment types;
- survey administration: mode(s), dates, number of contacts, types of contacts, median time to completion;
- disciplinary affiliation measurement:
  - measured dimensions: PhD, current department, professional society/association, primary targeted journals, peer perception, self-identification;
  - indicator type: closed-ended survey question, open-ended survey question; implied via sampling frame construction (not measured on survey);
  - most disaggregated field level: discipline, family, mix of disciplines and families.

In what follows, we describe the distribution of surveys for most of these characteristics. We refer to **Figures A-L**, which appear after this narrative on subsequent pages of this Supporting Information file.

Survey Administration over Time

**Figure A** displays the annual and cumulative number of unique surveys administered since 1955. The frequency of such surveys remained fairly low until the late 1980s, when it slightly increased. While it took approximately 40 years to reach a total of 20 surveys (i.e., 1955-1995), that total doubled within 10 more years (i.e., 1996-2005). Since the mid-2000s, scholars have administered a rather large number of surveys to scientist samples, culminating in a total of 115 by the end of 2023.

Survey Administration across Space

**Figure B** presents a world map that is color-shaded by the total number of unique surveys administered in each country. The lighter to darker gradient represents lesser to greater numbers of surveys. Any country with no shading at all had no such survey administered. **Figure C** offers an alternative way for displaying variation in survey administration by spatial-cultural regions. After bracketing out those 10 samples where the location of respondents was unspecified or otherwise opaque, 90.5% of the remaining surveys (95 of 105) were administered in the “core Anglosphere,” which contains Australia, Canada, New Zealand, the United Kingdom, and the United States of America (e.g., Szczepański 2025; Vucetic 2011). About half (49.5%) of the surveys were administered in Western Europe, while 21.9% were administered in Eastern Europe. Roughly a quarter (25.7%) were administered in Eastern or Southeastern Asia, while much lower percentages of the surveys were administered in Latin America and Brazil (10.5%), Western or Central Asia (4.8%), and Africa (2.9%).

Substantive Focus of Surveys

The surveys in this collection covered an array of substantive topics. **Figure D** briefly characterizes the patterns in surveys’ main foci. Approximately one-third of these were “general purpose” surveys with questions on a wide range of issues within and/or beyond science/academia. Another one-third of the surveys focused primarily on respondents’ scientific views (e.g., on epistemology, virtues, goals) or their scientific practices (e.g., data collection, publishing, data sharing) (16.5%) or on their views about and/or experiences with questionable research practices, ethical violations, or research misconduct (16.5%). One-fifth of surveys focused mainly on scientists’ political views and/or behaviors (12.2%) or on their perceptions of and/or experiences with political interference into science by elected officials, politicians, and activists (7.8%). The remaining 15% of surveys primarily focused on scientists’ professional development, mentoring, and other human capital endeavors (7.0%), on scientists’ views about and/or experiences with public/media communication training or efforts (6.1%), or on other topics (1.7%).

Sampling Designs of Surveys

**Figure E** displays the types of sampling designs used by researchers when administering the 115 surveys we reviewed. We were unable to determine the precise sampling design used with 7.0% of these surveys. This includes cases in which resulting publications or other project documentation lacked sufficient and clear sampling information and the few surveys administered cross-nationally that used sampling designs that varied considerably across nations. In 17.4% of cases, researchers attempted to conduct a census of a specific target population, even if the latter was rather circumscribed (e.g., researchers at a single university or scientists in a certain city).

Researchers administering slightly over half of the surveys in this collection employed some kind of probability-based sampling design. In 32.2% of cases, researchers constructed a sampling frame that closely approximated their target population, often using some sort of stratification scheme to reduce the likelihood of over- or under-sampling scientists from certain known groups (e.g., type of institution, scientific discipline, academic rank). In another 21.7% of cases, researchers employed some probabilistic procedure to select their sample—but from a sampling frame that did not closely approximate their target population (e.g., a marketing company’s list of university faculty members; current members of a professional association). In a final 21.7% of cases, researchers employed a non-probability sampling design, using such procedures as convenience, purposive, or snowball sampling.

Survey Administration Modes

We were able to determine the administration mode(s) for all but two surveys in our collection. **Figure F** displays trends in the cumulative frequencies of different survey administration modes between 1955 and 2023. The first survey (in 1955) was administered via face-to-face interviews, but no subsequent surveys used this mode. Every survey from then until 1991 was administered only as a postal mail questionnaire, and this mode was used exclusively in approximately 31.3% of surveys. The years 1992 to 2015 was a period of transition for the administration of surveys in our collection, as mail questionnaires gave way to online questionnaires. Increasingly frequent during this time were mixed-mode surveys. The first mixed-mode administration occurred in 1992, and the last occurred in 2018—culminating in 9.6% of all surveys in our collection.^[1]^ The first online-only questionnaire was administered in 2003, after which this mode quickly became the dominant one, eventually accounting for 56.5% of all surveys by 2023.

Survey Response Rates

We were unable to find sufficient information on response rate and sample size characteristics for many of the surveys administered outside the USA. Thus, we focus here on the 63 US-based surveys for which response rates either were disclosed or could be calculated. **Figure G** displays the response rate for these 63 surveys administered in the USA over the time period. We distinguish between one-time surveys and those administered as individual waves from one of six multi-wave survey projects. In most, but not all cases, the latter endeavors tended to have greater resources and personnel available to perform survey design and administration than did the former.

We see three compelling patterns in **Figure G**. **First**, survey response rates have decreased considerably over time. This is apparent when examining the median response rates for surveys across three time periods:^[2]^

- 50.4% for the 25 surveys between 1955 and 2004,
- 20.4% for the 22 surveys between 2005 and 2013, and
- 10.9% for the 16 surveys between 2014 and 2023.

Indeed, only one survey in this last time period had a response rate greater than 20%, and that was a 2018 survey of US and Canadian university scientists with a response rate of 20.6% (Niles et al. 2020).

**Second**, the response rates for different waves of the same multi-wave projects are fairly similar (and collectively tend to be noticeably higher than those for most one-off surveys). For instance, the response rates for four waves of the National Survey of Postsecondary Faculty ranged between 75.7% and 91.6%. The response rates for three waves of the Carnegie Survey of Faculty at US Colleges and Universities ranged between 45.3% and 54.5%, and those for eight waves of the Higher Education Research Institute Faculty Survey ranged between 33.0% and 46.0%. Finally, the response rates for six waves of the Ithaka S+R Faculty Survey ranged between 4.7% and 9.4%.

**Third**, the range of response rates for the 29 one-off surveys in **Figure G** is quite considerable, from a low of 3.3% for a 2022 survey about unpublished data and unused samples (Bowers et al. 2023) to a high of 72.0% for the 1999 North American Academic Study Survey (Rothman, Lichter, and Nevitte 2005). The mean and median response rates of 29.7% and 25.3%, respectively, suggest some modest amount of positive skewness for this distribution of 29 surveys.

Survey Sample Sizes

**Figure H** displays the sample size for 69 surveys administered in the USA over the time period for which such sample size information was reported. Since the sample size distribution is positively skewed, we used a logarithmic scale for the vertical axis (sample size) to help with vertical spacing of the data points. As with Figure SI7, we distinguish between one-time surveys and those administered as individual waves from one of six multi-wave survey projects.

We see two interesting patterns in **Figure H**. **First**, as we found with response rates, the sample sizes for different waves of the same multi-wave projects are fairly similar. For instance, the sample sizes for various waves of the same multi-wave survey endeavor seem to cluster together on this logarithmic scale. There is a median sample size of:

- 34,979 for 12 waves of the Higher Education Research Institute Faculty Survey,
- 21,690 for four waves of the National Survey of Postsecondary Faculty,
- 7,553 for six waves of the Ithaka S+R Faculty Survey, and
- 5,151 for five waves of the Carnegie Survey of Faculty at US Colleges and Universities.

**Second**, as we found with response rates, the range of sample sizes for the 31 one-off surveys in **Figure H** is rather considerable, from a low of 280 for a 2011 survey of Midwestern faculty (Motycka 2011) to a high of 42,345 for the 1972-1973 Teaching Faculty in American Survey (Bayer 1973). The median sample size for these 31 one-off surveys was 1,269, and only eight of them had a sample size greater than 2,500.

Disciplinary Affiliation Dimensions Measured

We were unable to identify which, if any, dimensions of disciplinary affiliation that scholars may have measured in 18 different surveys over the time period. In such cases, we could locate neither the administered survey nor any publications where scholars reported the instrument they used to measure respondents’ disciplinary affiliation. As such, **Figure I** displays the prevalence of different disciplinary affiliation measures in the remaining 97 surveys in our collection. We coded these measures according to the six dimensions of disciplinary affiliation listed at the end of Section 2.1: discipline of the respondent’s highest degree, their current department, their main professional society, the primary journals they target, their perceptions of how peers view them, and their own self-identity. We first discuss the prevalence of each dimension across all 97 surveys, before focusing more closely on the patterns among 57 surveys that measure only one dimension, 30 surveys that measured exactly two dimensions, and 10 surveys that measured exactly three dimensions.

Approximately 59.8% of surveys (58 of 97) included a disciplinary self-identity indicator that typically asked respondents to select or report their primary academic discipline or area of expertise or, framed more concretely, their primary field of research and/or teaching.^[3]^ The second most common indicator, appearing in 43.3% of these surveys (42 of 97), asked respondents to select or report the discipline of their highest degree.^[4]^ The third most common indicator, appearing in 41.2% of these surveys (40 of 97), asked respondents to select or report the discipline of their current department.

Only 4.1% of these surveys (4 of 97) asked respondents to select or report the discipline of their primary professional association or scholarly society, and just 3.1% of the surveys (3 of 97) asked respondents to select or report the discipline of their commonly targeted journals. None of the 97 surveys asked respondents to select or report the discipline that they think most peers associate with them (e.g., when asking them to serve on editorial boards, review panels, advisory boards, etc.).

We now discuss the patterns of disciplinary affiliation dimensions included in surveys that measure just one dimension (57 of 97), exactly two dimensions (30 of 97), and exactly three dimensions (10 of 97). None of the 97 surveys included indicators for four or more dimensions. Among those 57 surveys that measured only a single dimension of disciplinary affiliation, the most common dimension was self-identity (64.9%; 37 of 57 surveys), followed by current department (19.3%; 11 of 57), highest degree (10.5%; 6 of 57), and professional society (5.3%; 3 of 57). No surveys that featured only a single dimension of disciplinary affiliation asked about targeted journals or peers’ perceptions.

**Figure J** displays the relative frequency of all pairs of disciplinary affiliation indicators featured in the 30 surveys that measure exactly two dimensions. Sixty percent of these surveys (18 of 30) featured indicators of the discipline of respondents’ highest attained degree *and* their current department. Another 23.3% of these surveys (7 of 30) included indicators of the discipline of the respondents’ highest degree *and* self-identity. Much smaller percentages of surveys included the following pairs of indicators:

- 10% (3 of 30) included indicators of the discipline of the respondents primary targeted journals *and* self-identity;
- 3.3% (1 of 30) included indicators of the discipline of the respondents’ highest degree *and* professional association; and
- 3.3% (1 of 30) included indicators of the discipline of the respondents; current department *and* self-identity.

A final 10.3% of surveys (10 of 97) measured exactly three dimensions of disciplinary affiliation. Each of these 10 surveys—which have a shared lineage or “family tree”^[5]^—included the same combination of disciplinary affiliation indicators: respondents’ highest degree, current department, *and* self-identity.

Types of Indicators Used to Measure Scientific Field

We were unable to identify the types of direct or indirect indicators, if any, that scholars use to measure disciplinary affiliation in 17 different surveys over the time period. In such cases, we could locate neither the administered survey nor any publications where scholars reported the survey instrument they used to directly measure respondents’ disciplinary affiliation or the sampling frame information they used to indirectly capture respondents’ disciplinary affiliation. As such, **Figure K** displays the types of indicators scholars used to measure respondents’ disciplinary affiliation in 98 surveys in our collection.

In 85.7% of cases (84 of 97), researchers included one or more questions/indicators on their survey to *directly* measure respondents’ disciplinary affiliation. Briefly, 78.6% of surveys (77 of 98) included only a closed-ended (i.e., multiple choice) question(s); 3.1% of surveys (3 of 98) included only an open-ended question(s), which then required coding after data collection; and 4.1% of surveys (4 of 98) included both a closed-ended and an open-ended question.

In the remaining 14.3% of cases (14 of 98), researchers captured respondents’ disciplinary affiliation *indirectly* during their sampling frame construction—without including any question measuring it on their survey. These cases are represented by the blue pie slice in **Figure K**, with the corresponding stacked column specifying the particular techniques used. In such instances, scholars identified criteria during their sampling frame construction that they then used to place potential respondents into different disciplines. As such, these criteria are proxies for disciplinary affiliation:

- 8.2% (8 of 98) used respondents’ departmental or college affiliation;
- 4.1% (4 of 98) used respondents’ membership in a professional association or scholarly society;
- 1.0% (1 of 98) used the Journal Citation Reports journal classification category of respondents’ publications; and
- 1.0% (1 of 98) used respondents’ classification category in a governmental employment registry.

Levels of Disaggregation in Indicators Used to Measure Scientific Field

For 15 surveys in our collection, researchers either did not include an indicator to measure respondents’ disciplinary affiliation or did not report the categories from which respondents chose. As such, **Figure L** displays the prevalence of the lowest level of scientific field disaggregation in the remaining 100 surveys in our collection. Panel A presents the percentages for all 100 surveys, and Panel B presents these percentages for 65 US-based surveys. In 42.0% of all surveys (42 of 100) and 52.3% of US surveys (34 of 65), respondents selected from a list that only included disciplines (or were asked to report their discipline). In 31.0% of all surveys (31 of 100) and 16.9% of US surveys (11 of 65), respondents selected from a list that only included families (i.e., aggregations of groups of similar disciplines) (or were asked to report their family). Finally, in 27.0% of all surveys (27 of 100) and 30.8% of US surveys (20 of 65), respondents selected from a list that included a mix of disciplines and families.

**References**

Bayer, Alan E. 1973. *Teaching Faculty in America, 1972-1973*. American Council on Education (ACE) Research Report: Volume 8, Number 2. Washington, DC: American Council on Education.

Bowers, Emma C., Jimena Stephenson, Melissa Furlong, and Kenneth S. Ramos. 2023. “Scope and Financial Impact of Unpublished Data and Unused Samples among US Academic and Government Researchers.” *iScience* 26:107166.

Motycka, Eric D. 2011. *Development of an Instrument to Measure Faculty Adherence to the Norms of Science*. Doctoral Dissertation. Department of Educational Leadership. Terre Haute, IN: Indiana State University.

Niles, Meredith T., Lesley A. Schimanski, Erin C. McKiernan, Juan Pablo Alperin. 2020. “Why We Publish Where We Do: Faculty Publishing Values and Their Relationship to Review, Promotion, and Tenure Expectations.” *PLoS ONE* 15(3):e0228914.

Rothman, Stanley S., Robert Lichter, & Neil Nevitte. 2005. “Politics & Professional Advancement among College Faculty.” *The Forum* 3(1):article 2.

Szczepański, Jarosław. 2025. “The Anglosphere as Non-Contiguous Region: Remarks on CANZUK.” *Australian Journal of International Affairs* doi:10.1080/10357718.2025.2505070.

Vucetic, Srdjan. 2011. *The Anglosphere: A Genealogy of a Racialized Identity in International Relations*. Stanford, CA: Stanford University Press.

**Figure A**: Annual and Cumulative Number of Unique Surveys Administered to at Least Moderately Sized Samples of Scientists, 1955-2023

|  | Annual # of Surveys (left axis) | Cumulative # of Surveys (right axis) |
| --- | --- | --- |
| 1955 | 1 | 1 |
| 1956 | 0 | 1 |
| 1957 | 0 | 1 |
| 1958 | 0 | 1 |
| 1959 | 1 | 2 |
| 1960 | 0 | 2 |
| 1961 | 0 | 2 |
| 1962 | 1 | 3 |
| 1963 | 0 | 3 |
| 1964 | 1 | 4 |
| 1965 | 0 | 4 |
| 1966 | 0 | 4 |
| 1967 | 0 | 4 |
| 1968 | 0 | 4 |
| 1969 | 1 | 5 |
| 1970 | 0 | 5 |
| 1971 | 0 | 5 |
| 1972 | 1 | 6 |
| 1973 | 0 | 6 |
| 1974 | 0 | 6 |
| 1975 | 1 | 7 |
| 1976 | 0 | 7 |
| 1977 | 1 | 8 |
| 1978 | 0 | 8 |
| 1979 | 0 | 8 |
| 1980 | 0 | 8 |
| 1981 | 0 | 8 |
| 1982 | 0 | 8 |
| 1983 | 0 | 8 |
| 1984 | 1 | 9 |
| 1985 | 1 | 10 |
| 1986 | 0 | 10 |
| 1987 | 2 | 12 |
| 1988 | 0 | 12 |
| 1989 | 2 | 14 |
| 1990 | 0 | 14 |
| 1991 | 2 | 16 |
| 1992 | 4 | 20 |
| 1993 | 0 | 20 |
| 1994 | 0 | 20 |
| 1995 | 1 | 21 |
| 1996 | 0 | 21 |
| 1997 | 1 | 22 |
| 1998 | 2 | 24 |
| 1999 | 1 | 25 |
| 2000 | 2 | 27 |
| 2001 | 1 | 28 |
| 2002 | 1 | 29 |
| 2003 | 3 | 32 |
| 2004 | 2 | 34 |
| 2005 | 4 | 38 |
| 2006 | 4 | 42 |
| 2007 | 3 | 45 |
| 2008 | 0 | 45 |
| 2009 | 4 | 49 |
| 2010 | 5 | 54 |
| 2011 | 2 | 56 |
| 2012 | 5 | 61 |
| 2013 | 4 | 65 |
| 2014 | 1 | 66 |
| 2015 | 5 | 71 |
| 2016 | 4 | 75 |
| 2017 | 5 | 80 |
| 2018 | 9 | 89 |
| 2019 | 6 | 95 |
| 2020 | 3 | 98 |
| 2021 | 5 | 103 |
| 2022 | 9 | 112 |
| 2023 | 3 | 115 |

**Note**: Each survey is classified by its first year of administration (i.e., for those surveys administered across more than one calendar year.

**Figure B**: Number of Unique Surveys Administered in 56 Countries

**Note**: In addition to the nations displayed above, three surveys were administered to non-probability-based samples in unspecified countries, and seven others were administered to non-probability samples that spanned multiple countries.

**Figure C**: Number of Unique Surveys Administered in Seven Geographic or Cultural Regions

**Note**: The “core anglosphere” includes Australia, Canada, New Zealand, the United Kingdom, and the United States of America.

**Figure D**: Main Foci of 115 Unique Surveys

|  |  |
| --- | --- |
| General Purpose (N=37) | 37 |
| Regular Scientific Practices (N=19) | 19 |
| Misconduct, Questionable Research Practices, Ethical Violations (N=19) | 19 |
| Political Views/Behaviors (N=14) | 14 |
| Political Interference in Science (N=9) | 9 |
| Professional Development or Human Capital (N=8) | 8 |
| Communication/Engagement Training/Efforts (N=7) | 7 |
| Other (N=2) | 2 |

**Figure E**: General Type of Sampling Design for 115 Unique Surveys

|  | attempted census of target population (N=20) | probability-based design w/ sampling frame approximating target population (N=37) | probability-based design w/ sampling frame not approximating target population (N=25) | non-probability-based design (N=25) | sampling design could not be specified accurately (N=8) |
| --- | --- | --- | --- | --- | --- |
| 2 | 17.3913% | 32.1739% | 21.7391% | 21.7391% | 6.95652% |
| 3 | 0% | 0% | 0% | 0% | 0% |

**Note**: The “attempted census” category includes all of those studies that attempted a census of a specific target population, even if the latter is rather circumscribed. The first “probability-based design” categories typically includes studies that have used some sort of stratification scheme to reduce the likelihood of over- or under-sampling scientists in certain categories. The final category also includes five surveys administered cross-nationally where the sampling design varied considerably across nations.

**Figure F**: Cumulative Number of 113 Unique Surveys by Administration Mode, 1955-2023

|  | Face-to-Face Interview Only (N=1) | Mail Questionnaire Only (N=36) | Mixed-Mode Survey (N=11) | Online Questionnaire Only (N=65) |
| --- | --- | --- | --- | --- |
| 1955 | 1 | 0 | 0 | 0 |
| 1956 | 1 | 0 | 0 | 0 |
| 1957 | 1 | 0 | 0 | 0 |
| 1958 | 1 | 0 | 0 | 0 |
| 1959 | 1 | 1 | 0 | 0 |
| 1960 | 1 | 1 | 0 | 0 |
| 1961 | 1 | 1 | 0 | 0 |
| 1962 | 1 | 2 | 0 | 0 |
| 1963 | 1 | 2 | 0 | 0 |
| 1964 | 1 | 3 | 0 | 0 |
| 1965 | 1 | 3 | 0 | 0 |
| 1966 | 1 | 3 | 0 | 0 |
| 1967 | 1 | 3 | 0 | 0 |
| 1968 | 1 | 3 | 0 | 0 |
| 1969 | 1 | 4 | 0 | 0 |
| 1970 | 1 | 4 | 0 | 0 |
| 1971 | 1 | 4 | 0 | 0 |
| 1972 | 1 | 5 | 0 | 0 |
| 1973 | 1 | 5 | 0 | 0 |
| 1974 | 1 | 5 | 0 | 0 |
| 1975 | 1 | 6 | 0 | 0 |
| 1976 | 1 | 6 | 0 | 0 |
| 1977 | 1 | 7 | 0 | 0 |
| 1978 | 1 | 7 | 0 | 0 |
| 1979 | 1 | 7 | 0 | 0 |
| 1980 | 1 | 7 | 0 | 0 |
| 1981 | 1 | 7 | 0 | 0 |
| 1982 | 1 | 7 | 0 | 0 |
| 1983 | 1 | 7 | 0 | 0 |
| 1984 | 1 | 8 | 0 | 0 |
| 1985 | 1 | 9 | 0 | 0 |
| 1986 | 1 | 9 | 0 | 0 |
| 1987 | 1 | 11 | 0 | 0 |
| 1988 | 1 | 11 | 0 | 0 |
| 1989 | 1 | 13 | 0 | 0 |
| 1990 | 1 | 13 | 0 | 0 |
| 1991 | 1 | 15 | 0 | 0 |
| 1992 | 1 | 18 | 1 | 0 |
| 1993 | 1 | 18 | 1 | 0 |
| 1994 | 1 | 18 | 1 | 0 |
| 1995 | 1 | 19 | 1 | 0 |
| 1996 | 1 | 19 | 1 | 0 |
| 1997 | 1 | 20 | 1 | 0 |
| 1998 | 1 | 21 | 2 | 0 |
| 1999 | 1 | 21 | 2 | 0 |
| 2000 | 1 | 23 | 2 | 0 |
| 2001 | 1 | 24 | 2 | 0 |
| 2002 | 1 | 25 | 2 | 0 |
| 2003 | 1 | 26 | 3 | 1 |
| 2004 | 1 | 28 | 3 | 1 |
| 2005 | 1 | 29 | 3 | 4 |
| 2006 | 1 | 32 | 3 | 5 |
| 2007 | 1 | 33 | 4 | 6 |
| 2008 | 1 | 33 | 4 | 6 |
| 2009 | 1 | 34 | 5 | 8 |
| 2010 | 1 | 35 | 6 | 11 |
| 2011 | 1 | 35 | 6 | 13 |
| 2012 | 1 | 36 | 6 | 17 |
| 2013 | 1 | 36 | 8 | 19 |
| 2014 | 1 | 36 | 8 | 20 |
| 2015 | 1 | 36 | 8 | 25 |
| 2016 | 1 | 36 | 8 | 28 |
| 2017 | 1 | 36 | 9 | 32 |
| 2018 | 1 | 36 | 10 | 40 |
| 2019 | 1 | 36 | 10 | 46 |
| 2020 | 1 | 36 | 10 | 49 |
| 2021 | 1 | 36 | 10 | 54 |
| 2022 | 1 | 36 | 10 | 63 |
| 2023 | 1 | 36 | 10 | 66 |

**Note**: This figure does not include two surveys for which the primary administration mode was unknown.

**Figure G**: Response Rate for 63 Unique Surveys in the USA, 1955-2023

|  | Carnegie Survey |
| --- | --- |
| 1969 | 8.45% |
| 1975 | 45.3% |
| 1997 | 51.6% |
| 1989 | 54.5% |

|  | HERI Survey |
| --- | --- |
| 2007 | 33% |
| 2013 | 36.9% |
| 1989 | 38% |
| 2004 | 38% |
| 2010 | 39.7% |
| 2001 | 41% |
| 1995 | 42% |
| 1998 | 46% |
| 1992 | 61% |

|  | Ithaka S+R Survey |
| --- | --- |
| 2012 | 4.7% |
| 2021 | 5.2% |
| 2015 | 6.3% |
| 2018 | 7.3% |
| 2009 | 8.6% |
| 2006 | 9.4% |

|  | NSOPF Survey |
| --- | --- |
| 1987 | 75.7% |
| 2003 | 76% |
| 1992 | 86.6% |
| 1998 | 91.6% |

|  | Pew Survey |
| --- | --- |
| 2014 | 18.8% |
| 2009 | 25.3% |

|  | UCS Survey |
| --- | --- |
| 2022 | 3.9% |
| 2018 | 6.9% |
| 2011 | 14.2% |
| 2006 | 16.8% |
| 2015 | 18.6% |
| 2006 | 19% |
| 2010 | 21.6% |
| 2007 | 29.3% |
| 2005 | 29.4% |

|  | All Other Surveys |
| --- | --- |
| 2022 | 3.3% |
| 2019 | 7.2% |
| 2013 | 7.8% |
| 2012 | 8.5% |
| 2010 | 10.5% |
| 2016 | 10.6% |
| 2012 | 10.8% |
| 2018 | 11.2% |
| 2021 | 14.1% |
| 2018 | 14.1% |
| 2017 | 15% |
| 2019 | 18% |
| 2005 | 19.23% |
| 2018 | 20.6% |
| 2011 | 25.3% |
| 2010 | 26.3% |
| 2003 | 30.59% |
| 1991 | 32.2% |
| 2009 | 34.2% |
| 1972 | 39% |
| 2000 | 43.31% |
| 2002 | 47.2% |
| 2006 | 49.7% |
| 1977 | 50.4% |
| 1959 | 54.9% |
| 1991 | 59% |
| 1985 | 62.55% |
| 2003 | 63.3% |
| 1999 | 72% |

**Note**: This scatterplot displays data only from 63 of 74 available US surveys for which a response rate was reported.

**Figure H**: Sample Size for 69 Unique Surveys in the USA, 1955-2023

|  | Carnegie Survey |
| --- | --- |
| 1969 | 8478 |
| 1975 | 3536 |
| 1997 | 5151 |
| 1989 | 5450 |
| 1984 | 5057 |

|  | HERI Survey |
| --- | --- |
| 2007 | 34479 |
| 2013 | 24934 |
| 1989 | 35478 |
| 2004 | 65124 |
| 2010 | 37933 |
| 2001 | 55521 |
| 1995 | 33986 |
| 1998 | 55081 |
| 1992 | 43940 |
| 2016 | 33798 |
| 2019 | 24754 |
| 2022 | 13368 |

|  | Ithaka S+R Survey |
| --- | --- |
| 2012 | 7491 |
| 2021 | 7615 |
| 2015 | 9203 |
| 2018 | 10919 |
| 2009 | 3025 |
| 2006 | 4152 |
| 2012 | 3498 |
| 2015 | 6679 |

|  | NSOPF Survey |
| --- | --- |
| 1987 | 8382 |
| 2003 | 26110 |
| 1992 | 25780 |
| 1998 | 17600 |

|  | Pew Survey |
| --- | --- |
| 2014 | 3748 |
| 2009 | 2533 |

|  | UCS Survey |
| --- | --- |
| 2022 | 1828 |
| 2018 | 4211 |
| 2011 | 997 |
| 2006 | 997 |
| 2015 | 6999 |
| 2006 | 380 |
| 2010 | 1710 |
| 2007 | 1586 |
| 2005 | 414 |

|  | All Other Surveys |
| --- | --- |
| 2022 | 301 |
| 2019 | 1107 |
| 2013 | 390 |
| 2012 | 425 |
| 2010 | 13116 |
| 2016 | 613 |
| 2012 | 464 |
| 2018 | 508 |
| 2021 | 10071 |
| 2018 | 10706 |
| 2017 | 807 |
| 2019 | 1581 |
| 2005 | 1269 |
| 2018 | 338 |
| 2011 | 280 |
| 2010 | 2329 |
| 2003 | 1678 |
| 1991 | 478 |
| 2009 | 4131 |
| 1972 | 42345 |
| 2000 | 450 |
| 2002 | 3247 |
| 2006 | 1471 |
| 1977 | 4383 |
| 1959 | 2391 |
| 1991 | 1180 |
| 1985 | 997 |
| 2003 | 912 |
| 1999 | 1643 |
| 1955 | 2451 |
| 2015 | 4703 |
| 2022 | 485 |
| 2018 | 3659 |
| 2022 | 5420 |
| 2016 | 8364 |
| 2013 | 630 |
| 2020 | 6813 |
| 2017 | 1073 |
| 2021 | 917 |
| 2017 | 1141 |
| 2018 | 7291 |
| 2020 | 581 |
| 2021 | 1866 |
| 2009 | 2081 |
| 2000 | 3318 |
| 2022 | 11050 |
| 2005 | 1512 |
| 2004 | 915 |
| 2013 | 876 |
| 1987 | 5217 |
| 2005 | 1485 |
| 2023 | 316 |
| 1962 | 3006 |
| 2023 | 354 |
| 2023 | 364 |
| 2020 | 384 |
| 2019 | 457 |
| 2021 | 495 |
| 2022 | 590 |
| 2015 | 604 |
| 2022 | 704 |
| 2022 | 800 |
| 2017 | 954 |
| 2019 | 1246 |
| 1964 | 1385 |
| 2016 | 1798 |
| 2019 | 3759 |
| 2018 | 4221 |

**Note**: This scatterplot displays data only from 69 of 74 available US surveys for which a sample size was reported. The vertical axis uses a logarithmic scale.

**Figure I**: Frequency of Disciplinary Dimensions Measured in 97 Unique Surveys

|  | 57 Surveys Including Only 1 Dimension | 30 Surveys Including 2 Dimensions | 10 Surveys Including 3 Dimensions |
| --- | --- | --- | --- |
| PhD | 6 | 26 | 10 |
| Current Department | 11 | 19 | 10 |
| Professional Society | 3 | 1 | 0 |
| Primary Targeted Journals | 0 | 3 | 0 |
| Perceived Peer Perception | 0 | 0 | 0 |
| Self-Identity | 37 | 11 | 10 |

**Note**: This figure does not include 18 surveys that did not include an indicator to measure disciplinary affiliation.

**Figure J**: Frequency of All Pairs of Disciplinary Dimensions Measured in 30 Unique Surveys

**Note**: Thirty surveys over the time period included indicators of two dimensions of respondents’ disciplinarity. This figure displays all of the combinations of these pairs featured in these 30 surveys.

**Figure K**: Types of Indicators Used to Measure Scientific Field in 98 Unique Surveys

|  |  |
| --- | --- |
| closed-ended question(s) only | 78.5714% |
| open-ended question(s) only | 3.06122% |
| closed- & open-ended questions | 4.08163% |
| by respondent's departmental or college affiliation | 8.16327% |
| by respondent's professional society membership | 4.08163% |
| by JCR journal classification | 1.02041% |
| by governmental employment registry classification | 1.02041% |

**Note**: Approximately 14.3% of surveys did not include any such indicator. Rather, the researchers “measured” respondents’ scientific field indirectly during their sampling frame construction. The dark blue pie slice represents these cases, with the stacked column in the right breakout identifying techniques used.

**Figure L**: Percentages of Scientific Field Disaggregation Level Measured in Unique Surveys

**Panel A**: 100 Total Surveys	**Panel B**: 65 US Surveys

|  |  |
| --- | --- |
| Disciplines Only | 42% |
| Families Only | 31% |
| Mix of Disciplines and Families | 27% |

|  |  |
| --- | --- |
| Disciplines Only | 52.3077% |
| Families Only | 16.9231% |
| Mix of Disciplines and Families | 30.7692% |

**Notes**: This figure does not include 15 surveys that either did not include any indicator to measure respondent discipline or did but did not report the categories from which respondents chose.

1. The combinations in these mixed-mode surveys are: mail questionnaire and online questionnaire (6 surveys); telephone interview, mail questionnaire, and online questionnaire (2 surveys); telephone interview and mail questionnaire (1 survey); and telephone interview and online questionnaire (1 survey).
2. The mean response rates for these three time periods are similar: 52.4%, 21.8%, and 11.3%, respectively.
3. The questions stems for these self-identity indicators ranged from using more abstract to more concrete prompts. The former include examples such as asking respondents to identify or select their: “primary academic field,” “primary field or scientific discipline,” “area of expertise,” “primary area of expertise,” “subject area,” or “subject discipline(s) [in which] you work in/publish.” The latter include examples such as asking respondents to identify or select their: “present primary field of research,” “principal area of research,” “field of research,” “present principal teaching field,” “principal field or discipline of teaching,” or “academic discipline or field of [their] current teaching.”
4. Because many of these surveys also sampled scientists without terminal or PhD degrees, they tended to ask more generally about the respondent’s “highest degree,” “highest attained education,” “highest graduate degree,” or “highest postgraduate degree.”
5. These 10 surveys are all associated with the Carnegie Foundation for the Advancement of Teaching, essentially occupying two related branches of the same “family tree.” One branch started with the 1969 Carnegie Survey of Faculty at US Colleges and Universities, which then had subsequent waves in 1975, 1984, 1989, 1992, and 1997. The 1972-1972 Teaching Faculty in America survey replicated the 1969 Carnegie Survey, and the 1977 Survey of the American Professoriate replicated the 1975 Carnegie Survey. The second branch started with the 1992-1993 Carnegie Survey of the Academic Profession (also known as the 1992-1993 International Survey of the Academic Profession). While this initial survey did not include all three indicators, two subsequent surveys—the 2007-2008 Changing Academic Profession Survey and the 2012-2013 Academic Profession in Asia Survey—that build upon this international project do include the same three disciplinary affiliation indicators.
