# Supplementary material for: Validating a novel measure of scientists’ disciplinary affiliation that captures its complexity and plasticity
Source: PLoS One. 2026 Aug 26;21(8):e0346268. doi: 10.1371/journal.pone.0346268 (PMC13514013; doi:10.1371/journal.pone.0346268)
Supplement: Supplementary file 3 [file pone.0346268.s003.docx]

**S3 File. National Sampling Design and Correspondence with Potential Participants**

for

*Validating a novel measure of scientists’ disciplinary affiliation that captures its complexity and plasticity*

Table of Contents	Page

**Table A**	Key Details of our National Sampling Design	2

**Table B**	Multi-Stage Sampling Units	3

**Table C**	Stratification Scheme for Selecting Stage 1 Institutions	4

**Table D**	Selected Stage 1 Institutions (with Stratification Percentages)	5

**Table E**	Key Characteristics of All Stage 1 Institutions by Inclusion in Sampling Frame	6

**Table F**	Selection Guidelines for Identifying Stage 2 Departments in Each Stage 1 Institution	7

**Table G**	Number of Stage 2 Departments and Stage 3 Faculty in Each Stage 1 Institution	9

**Table H**	Automated Web Scraping and Human Labor Processes for Generating the Full Listing of

Stage 3 Faculty	11

**Table I**	List of Correspondences with our Contacted Sample	13

**Figure A**	Survey Respondent Steps to Completion	14

**Table A**: Key Details of our National Sampling Design

| **Sampling Design** | multi-stage sampling design^1^ with proportionate stratified random sampling^2^ in Stage 1 (and Stage 3) |
| --- | --- |
| **Target Population** | currently employed tenure-system faculty in three broad science families (life sciences, physical and earth sciences, and social and behavioral sciences) at major US research universities |
| **Sampling Frame** | tenure-system faculty with a PhD in one of 20 science disciplines (from across the life sciences, physical and earth sciences, and social and behavioral sciences) employed at 83 Carnegie R1 universities during the 2023-2024 academic year |
| **Contacted Potential Respondents** | 23,462 |
| **AAPOR Response Rate 1** | 17.53% |
| **Sample Size** | 4,113 completed surveys by individual scientists |

^1^ Multi-stage sampling is optimal (and cost-effective) when we have a geographically dispersed (e.g., national) target population for which a comprehensive list does not exist.

^2^ Proportionate stratified random sampling (in which we divide units at a stage into similar/known groups and take a simple random sample within each group) at a stage ensures we do not under- or over-sample on certain unit characteristics that might otherwise happen with simple random sampling.

**Table B**: Multi-Stage Sampling Units

| **Sampling Stage** | **Sampling Unit** | **Sampling Technique** | **Strata** | **Selected Units** |
| --- | --- | --- | --- | --- |
| **Stage 1** | US university with some doctoral programs and much research activity | proportionate stratified random sampling | *geopolitical region* (9 Census divisions) and *institutional control* (public or private) | 83 Carnegie R1 institutions |
| **Stage 2** | science-focused academic departments (and schools, programs, institutes, and centers) | exhaustive selection of all candidate departments (and schools, programs, institutes, and centers) |  | 1,509 academic units |
| **Stage 3** | tenure-system faculty member | exhaustive selection of all tenure-system faculty with a PhD in one of our 20 selected scientific disciplines  (*de facto* proportionate stratified sampling) | *university* (83) and *PhD discipline* (20) | 23,462 scientists |

**Table C**: Stratification Scheme for Selecting Stage 1 Institutions

| **Census Division** | **Institutional Control** | **# among 146 Carnegie R1 Institutions** | **% among 146 Carnegie R1 Institutions** | **# in 83 Stage 1 Institutions** |
| --- | --- | --- | --- | --- |
| New England | Public | 4 | 2.74% | 2 |
| New England | Private | 10 | 6.85% | 6 |
| Mid-Atlantic | Public | 10 | 6.85% | 6 |
| Mid-Atlantic | Private | 10 | 6.85% | 6 |
| East North Central | Public | 13 | 8.90% | 7 |
| East North Central | Private | 4 | 2.74% | 2 |
| West North Central | Public | 8 | 5.48% | 5 |
| West North Central | Private | 1 | 0.68% | 1 |
| South Atlantic | Public | 22 | 15.07% | 12 |
| South Atlantic | Private | 5 | 3.42% | 3 |
| East South Central | Public | 11 | 7.53% | 6 |
| East South Central | Private | 1 | 0.68% | 1 |
| West South Central | Public | 14 | 9.59% | 8 |
| West South Central | Private | 3 | 2.05% | 2 |
| Mountain | Public | 13 | 8.90% | 6 |
| Mountain | Private | 1 | 0.68% | 1 |
| Pacific | Public | 13 | 8.90% | 7 |
| Pacific | Private | 3 | 2.05% | 2 |

**Table D**: Selected Stage 1 Institutions (with Stratification Percentages)

|  | **Public Institutions** (N=59) | | **Private Institutions** (N=24) | |
| --- | --- | --- | --- | --- |
| **Census Division** | **Stratification %** | **Selected Institutions** | **Stratification %** | **Selected Institutions** |
| **New England** (N=8) | 2.74% | University of New Hampshire; University of Connecticut | 6.85% | Brandeis University; Northeastern University; Boston University; Massachusetts Institute of Technology; Brown University; Yale University |
| **Mid-Atlantic** (N=12) | 6.85% | University of Buffalo; University of Albany, SUNY; New Jersey Institute of Technology; University of Pittsburgh; Rutgers University-New Brunswick; CUNY Graduate School and University Center | 6.85% | Rensselaer Polytechnic Institute; Columbia University; Cornell University; Syracuse University; New York University; Princeton University |
| **East North Central** (N=9) | 8.90% | Purdue University; Wayne State University; University of Cincinnati; University of Illinois, Chicago; Ohio State University; Ohio University-Main Campus; Indiana University | 2.74% | Case Western Reserve University; University of Notre Dame |
| **West North Central** (N=6) | 5.48% | University of Nebraska-Lincoln; Iowa State University; University of Kansas; University of Minnesota-Twin Cities; Kansas State University | 0.68% | Washington University in St. Louis |
| **South Atlantic** (N=15) | 15.07% | Georgia State University; Clemson University; University of Delaware; University of Central Florida; West Virginia University; Georgia Institute of Technology; University of South Florida; George Washington University; Florida International University; University of Maryland-Baltimore County; University of South Carolina-Columbia; Virginia Commonwealth University | 3.42% | University of Miami; Johns Hopkins University; Duke University |
| **East South Central** (N=7) | 7.53% | University of Mississippi; University of Tennessee-Knoxville; University of Alabama in Huntsville; University of Louisville; University of Memphis; University of Kentucky | 0.68% | Vanderbilt University |
| **West South Central** (N=10) | 9.59% | Texas Tech University; University of Texas at Austin; University of Arkansas; University of Texas at San Antonio; Louisiana State University; Oklahoma State University; University of Louisiana at Lafayette; University of North Texas | 2.05% | Rice University; Tulane University of Louisiana |
| **Mountain** (N=7) | 8.90% | Montana State University; Colorado State University; University of Colorado Denver/Anschutz Medical Campus; University of Arizona; University of Nevada-Las Vegas; Utah State University | 0.68% | University of Denver |
| **Pacific** (N=9) | 8.90% | Washington State University; University of California-Santa Cruz; University of California-Los Angeles; University of California-Irvine; University of Oregon; University of California-Riverside; University of California-Santa Barbara | 2.05% | California Institute of Technology; Stanford University |

**Table E**: Key Characteristics of All Stage 1 Institutions by Inclusion in Sampling Frame

| **Criterion** | **Indicator** | **Data Source** | **83 Institutions in Sampling Frame** | **63 Institutions Not in Sampling Frame** | **P-Value for Difference-in-Means/Proportions** |
| --- | --- | --- | --- | --- | --- |
| institutional classification | % AAU institutions | [American Association of Universities](https://www.aau.edu/who-we-are/our-members/aau-member-universities-year-entry) | 41.0% | 46.0% | 0.544 |
| institutional control | % public institutions | [American Association of Universities](https://www.aau.edu/who-we-are/our-members/aau-member-universities-year-entry) | 71.1% | 77.8% | 0.365 |
| institutional mission | % land-grant institution (under 1862 or 1890 Morrill Acts) | [US Department of Agriculture](https://www.nifa.usda.gov/about-nifa/how-we-work/partnerships/land-grant-colleges-universities) | 28.9% | 30.2% | 0.871 |
| undergraduate student body size | mean (and median) full-time on-campus UG enrollment (fall 2022) | [Integrated Postsecondary Education Data System](https://nces.ed.gov/ipeds/) | 17,220  (16,729) | 19,754  (18,479) | 0.147 |
| graduate student body size | mean (and median) full-time on-campus G enrollment (fall 2022) | [Integrated Postsecondary Education Data System](https://nces.ed.gov/ipeds/) | 5,579  (4,759) | 6,411  (5,713) | 0.232 |
| tenure-system faculty size | mean (and median) full-time tenure-system faculty & academic staff (fall 2022) | [Integrated Postsecondary Education Data System](https://nces.ed.gov/ipeds/) | 1,057  (917) | 1,176  (1,013) | 0.233 |
| non-tenure-system faculty size | mean (and median) full-time non-tenure-system faculty & academic staff (fall 2022) | [Integrated Postsecondary Education Data System](https://nces.ed.gov/ipeds/) | 888  (620) | 1,052  (732) | 0.261 |
| residential profile | % with at least 50% of full-time degree-seeking UG students living on campus | [Carnegie Classification of Institutions of Higher Education](https://carnegieclassifications.acenet.edu/carnegie-classification/classification-methodology/graduate-instructional-program-classification/) | 77.1% | 79.4% | 0.746 |
| enrollment profile | % with G students at least 25% of total enrollment | [Carnegie Classification of Institutions of Higher Education](https://carnegieclassifications.acenet.edu/carnegie-classification/classification-methodology/graduate-instructional-program-classification/) | 43.4% | 38.1% | 0.524 |
| undergraduate program profile | % with more than 40% of all bachelor’s degrees in arts and sciences | [Carnegie Classification of Institutions of Higher Education](https://carnegieclassifications.acenet.edu/carnegie-classification/classification-methodology/graduate-instructional-program-classification/) | 67.5% | 69.8% | 0.762 |
| presence of STEM professional school | % with on-campus medical or veterinary school | [Carnegie Classification of Institutions of Higher Education](https://carnegieclassifications.acenet.edu/carnegie-classification/classification-methodology/graduate-instructional-program-classification/) | 60.2% | 63.5% | 0.692 |
| general science federal grants secured | mean (and median) # of new NSF grants (annual average, FY19-FY23) | [National Science Foundation](https://dellweb.bfa.nsf.gov/starth.asp) | 84.4  (73.2) | 104.5  (77.4) | 0.067 |
| general science federal grant $ secured | mean (and median) $ in new NSF grants (annual average, FY19-FY23) | [National Science Foundation](https://dellweb.bfa.nsf.gov/starth.asp) | $31,779,198  ($24,076,000) | $40,866,641  ($29,429,000) | 0.065 |

**Table F**: Selection Guidelines for Identifying Stage 2 Departments in Each Stage 1 Institution

| **Step** | **Principle** | | |
| --- | --- | --- | --- |
| 1. Map the organizational structure of hierarchically embedded academic units. | Most institutions have just two hierarchical levels:  highest: College/Division/School &  lowest: Department/School.  Some institutions have three hierarchical levels:  highest: College/School  middle: School/Division  lowest: Department/School  We should realize that different institutions use different names for their different levels. | | |
| 2. Identify all science-related top-level Colleges, Divisions, and Schools that house most science departments in each institution, and create an exhaustive list of such departments. | Nearly all of the tenure-system faculty in our 20 scientific disciplines are appointed in science departments that are located in top-level academic units with the following names: | | |
|  | College/Division/School of:   - Agricultural Sciences and Natural Resources - Agriculture - Agriculture and Applied Sciences - Agriculture and Life Sciences - Agriculture and Natural Resources - Agriculture, Food, and Environment - Agriculture, Forestry, and Life Sciences - Agriculture, Human, & Natural Resource Sciences - Agriculture, Natural Resources, and Design - Arts and Sciences - Arts, Humanities, and Social Sciences - Arts, Sciences, and Education - Behavioral, Social, and Health Sciences - Biological Sciences - Earth, Ocean, and Environment - Environmental and Biological Sciences - Food, Agricultural, and Environmental Sciences - Food, Agricultural, and Natural Resource Sciences - Geosciences - Humanities and Sciences | | College/Division/School of:   - Humanities and Social Sciences - Humanities, Arts, and Social Sciences - Letters and Science - Liberal Arts - Liberal Arts and Sciences - Liberal Arts and Social Sciences - Life Sciences and Agriculture - Marine Science - Marine, Atmospheric, and Earth Science - Natural and Agricultural Sciences - Natural and Mathematical Sciences - Natural Resources - Natural Science(s) - Science(s) - Science(s) and Liberal Arts - Social Science(s) - Social Sciences and Humanities - Sustainability - the Coast and Environment - the Environment |
| 3. Check all other top-level academic units for any additional science-related departments, especially such top-level academic units that focus on:   - human or veterinary medicine; - business; - engineering; and - public policy, affairs, or administration. | A. Science-related departments may be located in top-level Colleges, Divisions, or Schools with the following names:   - The College - College of General Studies - College of World Studies - [Name] College   B. Colleges/Divisions/Schools of human or veterinary medicine often house some “basic science” departments as well as typically more numerous “clinical science” departments. We should include all such basic science departments (which tend to be in the biological and life sciences) that at least somewhat align with our 20 selected scientific disciplines.  C. Some Colleges/Divisions/Schools of business or management include a department of economics. This may be in addition to, or instead of, a department of economics in a College/Division/School of social science.  D. Some Colleges/Divisions/Schools of engineering house science-related departments with faculty from the physical and environmental sciences. Examples of such departments are:   - Department of Civil and Environmental Engineering and Earth Sciences - Earth and Atmospheric Sciences - Environmental Engineering and Earth Sciences - School of Applied and Engineering Physics   E. Some institutions contain Colleges/Divisions/Schools of public policy, affairs, or administration, which sometimes may house departments of political science, government, sociology, and geography. These top-level academic units often have names such as the following. | | |
|  | College/Division/School of:   - Citizenship and Public Affairs - International Affairs - International and Public Affairs - Policy Studies - Public Affairs - Public Affairs and Policy | College/Division/School of:   - Public and Environmental Affairs - Public and International Affairs - Public Policy - Public Policy and Administration - Public Policy and Public Affairs - Public Service | |
| 4. Check all top-level academic units for any non-departmental lower-level academic units (i.e., programs, centers, and institutes) that may house tenure-system faculty in one of our 20 selected disciplines. | Tenure-system faculty—especially those in the social and behavioral sciences—in some institutions are appointed in other lower-level academic units such as Programs, Centers, and Institutes. We include all such lower-level academic units in each of the four following categories:   - Racial/Ethnic or Regional Studies   - African Studies; Africana Studies; African and African American Studies; African American and Diaspora Studies; Black Studies; etc.   - American Studies; American and New England Studies; etc.   - American Indian Studies; American Indian and Indigenous Studies; Indigenous Studies; Native American Studies; etc.   - Asian Studies; Asian American Studies; Global Asian Studies; East Asian Studies;   - Ethnic Studies; Critical Race and Ethnic Studies; etc.   - European Studies; etc.   - Caribbean and Latin American Studies; Central American Studies; Chicano and Latino Studies; Hispanic Studies; Latin American Studies; Latino and Caribbean Studies; Mexican American Studies; etc.   - Middle East and North Africa Studies; etc. - Feminist Studies; Gender Studies; Women’s Studies; Women’s and Gender Studies; Women’s, Gender, and Sexuality Studies; etc. - Science Studies; Science and Technology Studies; Science, Technology, and Society; etc. - Urban Studies; Urban and Public Affairs; Urban and Regional Planning; etc. | | |

The Graduate Research Assistant performing the subsequent web scraping of institutional directories discovered that some programs, centers, and institutes either had no tenure-system science faculty at all or had no tenure-system science faculty who were not also appointed in an academic department on our initial list. As such, we ultimately removed a few dozen such units from our final listing of Stage 2 departments—bringing the final total to 1,509.

**Table G**: Number of Stage 2 Departments and Stage 3 Faculty in Each Stage 1 Institution

| **Census Division** | **Institutional Control** | **Stage 1 Institution** | **# of Stage 2 Departments** | **# of Stage 3 Faculty** |
| --- | --- | --- | --- | --- |
| New England | public | University of New Hampshire-Main Campus | 17 | 144 |
|  |  | University of Connecticut | 20 | 434 |
|  | private | Brandeis University | 11 | 144 |
|  |  | Boston University | 23 | 338 |
|  |  | Massachusetts Institute of Technology | 10 | 307 |
|  |  | Brown University | 15 | 291 |
|  |  | Yale University | 21 | 419 |
|  |  | Northeastern University | 21 | 216 |
| Mid-Atlantic | public | University at Buffalo | 21 | 239 |
|  |  | University of Pittsburgh-Pittsburgh Campus | 20 | 352 |
|  |  | Rutgers University-New Brunswick | 27 | 426 |
|  |  | State University of New York at Albany | 14 | 190 |
|  |  | New Jersey Institute of Technology | 4 | 45 |
|  |  | City University of New York Graduate School & University Center | 19 | 624 |
|  | private | Columbia University | 36 | 491 |
|  |  | Cornell University | 37 | 635 |
|  |  | New York University | 15 | 326 |
|  |  | Princeton University | 22 | 348 |
|  |  | Rensselaer Polytechnic Institute | 7 | 43 |
|  |  | Syracuse University | 14 | 188 |
| East North Central | public | Purdue University-Main Campus | 19 | 455 |
|  |  | Ohio State University-Main Campus | 35 | 669 |
|  |  | Indiana University-Bloomington | 23 | 388 |
|  |  | Wayne State University | 15 | 170 |
|  |  | University of Cincinnati-Main Campus | 14 | 178 |
|  |  | University of Illinois, Chicago | 14 | 214 |
|  |  | Ohio University-Main Campus | 13 | 173 |
|  | private | Case Western Reserve University | 17 | 191 |
|  |  | University of Notre Dame | 9 | 227 |
| West North Central | public | University of Kansas | 15 | 207 |
|  |  | University of Minnesota-Twin Cities | 28 | 534 |
|  |  | University of Nebraska-Lincoln | 21 | 317 |
|  |  | Iowa State University | 17 | 288 |
|  |  | Kansas State University | 19 | 278 |
|  | private | Washington University in St Louis | 25 | 337 |
| South Atlantic | public | Georgia Institute of Technology-Main Campus | 9 | 221 |
|  |  | Georgia State University | 15 | 190 |
|  |  | Clemson University | 14 | 234 |
|  |  | University of Delaware | 16 | 298 |
|  |  | University of Central Florida | 9 | 204 |
|  |  | West Virginia University | 17 | 220 |
|  |  | University of South Florida | 12 | 282 |
|  |  | George Washington University | 18 | 226 |
|  |  | Florida International University | 9 | 191 |
|  |  | University of Maryland-Baltimore County | 12 | 147 |
|  |  | University of South Carolina-Columbia | 17 | 241 |
|  |  | Virginia Commonwealth University | 18 | 202 |
|  | private | Johns Hopkins University | 28 | 300 |
|  |  | Duke University | 20 | 406 |
|  |  | University of Miami | 20 | 210 |
| East South Central | public | University of Mississippi | 9 | 126 |
|  |  | University of Tennessee-Knoxville | 20 | 367 |
|  |  | University of Alabama in Huntsville | 10 | 66 |
|  |  | University of Louisville | 16 | 185 |
|  |  | University of Memphis | 13 | 124 |
|  |  | University of Kentucky | 31 | 418 |
|  | private | Vanderbilt University | 17 | 276 |
| West South Central | public | University of Texas at Austin | 26 | 593 |
|  |  | Texas Tech University | 14 | 249 |
|  |  | University of Arkansas | 16 | 242 |
|  |  | University of Texas at San Antonio | 12 | 184 |
|  |  | Louisiana State University & Agricultural & Mechanical College | 26 | 351 |
|  |  | Oklahoma State University-Main Campus | 22 | 276 |
|  |  | University of Louisiana at Lafayette | 9 | 74 |
|  |  | University of North Texas | 9 | 142 |
|  | private | Rice University | 12 | 194 |
|  |  | Tulane University of Louisiana | 22 | 205 |
| Mountain | public | University of Arizona | 33 | 581 |
|  |  | Montana State University | 15 | 183 |
|  |  | Colorado State University-Fort Collins | 24 | 413 |
|  |  | University of Colorado Denver/Anschutz Medical Campus | 15 | 168 |
|  |  | University of Nevada-Las Vegas | 10 | 184 |
|  |  | Utah State University | 17 | 228 |
|  | private | University of Denver | 13 | 75 |
| Pacific | public | University of California-Santa Cruz | 17 | 288 |
|  |  | University of California-Los Angeles | 30 | 521 |
|  |  | University of California-Irvine | 20 | 419 |
|  |  | University of Oregon | 30 | 233 |
|  |  | University of California-Santa Barbara | 18 | 323 |
|  |  | Washington State University | 22 | 377 |
|  |  | University of California-Riverside | 20 | 344 |
|  | private | California Institute of Technology | 22 | 177 |
|  |  | Stanford University | 27 | 478 |
|  |  |  | **1,509** | **23,462** |

**Table H**: Automated Web Scraping and Human Labor Processes for Generating the Full Listing of Stage 3 Faculty

| **Domain** | **Step** | **Action** |
| --- | --- | --- |
| Automated Web Scraping | 1 | Graduate Research Assistant #1 (GRA1) created a Python-based framework for managing a suite of web scraping tools for use on university websites. |
|  | 2 | GRA1 assembled a suite of web scraping tools to extract from departmental directory pages and individual faculty profile/bio pages the following information for each tenure-system faculty member in each Stage 2 department:   - first name; middle name/initial; last name; - tenure-system rank; - university email address; - PhD year; PhD institution; PhD discipline; - named/endowed professorship title (as applicable); - administrative position title (as applicable); and - university mailing address: street address, city, state, ZIP code. |
|  | 3 | GRA1 applied this suite of web scraping tools to a few Stage 1 institutions and then adapted and revised this suite as necessary for use with subsequent Stage 1 institutions—resolving new problems as they emerged. |
|  | 4 | GRA1 produced a separate Excel file for each university containing the extracted information for all tenure-system faculty in all Stage 2 departments for review. |
| Data File Management | 5 | GRA2 prepared each Stage 1 institution’s Excel file for subsequent manual data recording, cleaning, and quality control checks by:   - adding additional columns to facilitate review; - searching for and characterizing missing information, inconsistencies, and other potential issues; and - assigning the prepped file for delivery to one of our nine Undergraduate Research Assistants (URAs). |
| Manual Data Recording and Cleaning | 6 | GRA2 supervised our team of nine URAs while they performed the following data recording and cleaning tasks on each Stage 1 institution’s Excel file:   - confirmed the accuracy of extracted information and corrected any inaccuracies (e.g., imported unrecognized characters); - confirmed the placement/location of extracted information and corrected any mistakes (e.g., move information that was mistakenly extracted into the wrong column); and - supplied any missing information that was not extracted (e.g., email addresses, PhD year, PhD discipline, and PhD institution). |
| Data File Management, Consolidation, and Manual Quality Control Checks | 7 | Co-Principal Investigator #1 (Co-PI1) reviewed each completed Stage 1 institution’s Excel file and identified and corrected any clear/obvious inaccuracies:   - imported unrecognized characters; - spaces within email addresses; - “%20,” “Lab,” and “Web” after some email addresses; - Chinese characters; - additional space or tab at beginning of entry/cell; and - an * or a ^ at the end of some last names.   Co-PI1 also revised the entries in the first/middle/last name, named position, and administrative position columns as follows:   - moved the beginning of a compound last name (da, de, St., Van, van der, von, etc.) from the “Middle Name” cell to the beginning of the “Last Name” cell; - made sure the content is in the correct column; and - checked for any indication that a faculty member is emeritus or non-tenure-system. |
|  | 8 | Co-PI1 then merged each Stage 1 institution’s revised Excel file into a pooled sampling frame file and managed successive revisions of this file. |
|  | 9 | Co-PI1 removed duplicate entries from the pooled sampling frame file by sorting all cases by email address and using conditional formatting to identify duplicate email addresses. He deleted a scientist’s “duplicate” case/row that represented their secondary or courtesy appointment:   - this usually was a center, institute, or program outside of their one academic department; - this sometimes was another department further from their PhD discipline than the department in their other entry; and - this rarely was a repeated entry from the same department. |
|  | 10 | Co-PI1 removed additional entries from the pooled sampling frame file when they fell into at least one of the following three categories:   - non-tenure-system faculty:   - Visiting Assistant Professor/Associate Professor/Professor,   - Adjunct Assistant Professor/Associate Professor/Professor,   - Assistant Professor/Associate Professor/Professor of Practice or of the Practice,   - Research Assistant Professor/Associate Professor/Professor,   - Clinical Assistant Professor/Associate Professor/Professor,   - Service Assistant Professor/Associate Professor/Professor,   - Honorary Assistant Professor/Associate Professor/Professor,   - Lecturer/Senior Lecturer,   - Instructor,   - Research Scientist,   - Assistant Professor/Associate Professor/Professor in Residence; - emeriti/retired faculty; - faculty without an earned PhD; and - faculty with an earned PhD in a discipline outside of our focus (e.g., engineering, math/stats, arts and humanities, clinical medicine, etc.). |

**Table I**: List of Correspondences with our Contacted Sample

| **Planned Correspondence** | **Mailing Date** | **Approximate Contact Date** |
| --- | --- | --- |
| pre-letter postcard | Monday September 9 | Thursday September 12 –  Monday September 16 |
| cover letter with survey  link and token gift | Friday September 13th | Tuesday September 17 –  Thursday September 19 |
| first reminder email |  | Tuesday September 24 |
| follow-up postcard | Thursday September 26 | Tuesday October 1 –  Thursday October 3 |
| second reminder email |  | Tuesday October 8 |
| follow-up letter with survey  link and token gift | Wednesday October 9 | Monday October 14 –  Wednesday October 16 |
| third reminder email |  | Monday October 21 |
| fourth reminder email |  | Monday October 28 |

**Figure A**: Survey Respondent Steps to Completion

|  |  |  |  |  |  |  | & Who Completed  the Survey | | → | **4,113** |  |  |  |
| --- | --- | --- | --- | --- | --- | --- | --- | --- | --- | --- | --- | --- | --- |
|  |  |  |  |  |  | & Who Started  the Survey | | → | **5,142** | **1029** | ← | # who *didn’t*  *complete the survey* | |
|  |  |  |  |  | & Who Agreed  to Participate | | → | **5,701** | **559** | ← | # who *didn’t*  *start the survey* | |  |
|  |  |  |  | & Who Opened  the Survey | | → | **5,772** | **71** | ← | # who *didn’t agree*  *to participate* | |  |  |
|  |  |  | & Who Opened at  Least One Email | | → | **14,087** | **8,315** | ← | # who *never*  *opened the survey* | |  |  |  |
|  |  | & Who Didn’t Opt Our or Unsubscribe | | → | **22,956** | **8,869** | ← | # who *didn’t*  *open any email* | |  |  |  |  |
|  | & Contacted  at Least Once | | → | **23,002** | **46** | ← | # *unsubscribed or opted out*  after at least one email | | |  |  |  |  |
| # We Attempted to Contact | | → | **23,462** | **460** | ← | # of *email bouncebacks*  we never contacted | | |  |  |  |  |  |
