# Supplementary material for: Validating a novel measure of scientists’ disciplinary affiliation that captures its complexity and plasticity
Source: PLoS One. 2026 Aug 26;21(8):e0346268. doi: 10.1371/journal.pone.0346268 (PMC13514013; doi:10.1371/journal.pone.0346268)
Supplement: Supplementary file 4 [file pone.0346268.s004.docx]

**S4 File. Additional Results of Data Analyses**

for

*Validating a novel measure of scientists’ disciplinary affiliation that captures its complexity and plasticity*

Table of Contents	Page

**Supporting**

**Text A**	Confirmatory Factor Analysis Model Results for Our *CDR Experience Scale*	2

**Supporting**

**Text B**	Additional Non-Parametric and Parametric Tests of Association	3

**Table A**	Results from One-Way ANOVA of Years since PhD on Five Dimensions of Disciplinarity	4

**Table B**	Results from One-Way ANOVA of Approximate Number of Cross-Disciplinary Research

Projects on Five Dimensions of Disciplinarity	5

**Table C**	Results from One-Way ANOVA of PhD Science Family on Five Dimensions of Disciplinarity	6

**Figure A**	Radar Figures for Scientists’ Five-Dimension Disciplinarity by PhD Science Discipline	7

**Supporting**

**Text C**	Extended Discussion of the Substantive Focus of Respondent’s Primary Department	8

**Supporting**

**Text D**	Additional Information on Scientists Who Have Changed Their Discipline	9

**Figure B**	Percentages of Respondents in 20 PhD Disciplines Who Changed Their Scientific Discipline

(with 95% Confidence Interval)	10

**Table D**	Bivariate Correlations between Five Disciplinarity Dimensions and Nominal Discipline

Change (N=4,113)	11

**Supporting Text A**: Confirmatory Factor Analysis Model Results for Our *CDR Experience Scale*

The three indicators of CDR Experience have the following standardized factor loading (λ) and squared loading (λ^2^) values:

- number of projects outside discipline but within family: λ=0.66 (λ^2^=0.44),
- number of projects outside family but within science: λ=0.81 (λ^2^=0.66), and
- number of projects outside science: λ=0.57 (λ^2^=0.32).

Such loadings suggest acceptable to strong internal consistency. Further, the range of the λ^2^ values signals moderate to strong relationships between the underlying construct (i.e., latent variable) and each of the indicators. Briefly, the former explains between 32% and 66% of the variation in these three indicators—signifying a good fit between the latent variable and our three observed indicators we are using to measure it.

**Supporting Text B**: Additional Non-Parametric and Parametric Tests of Association

To inspect the robustness of this relationship, we ran additional non-parametric and parametric tests of association, and all confirm the general pattern we found with Spearman’s Rank Correlation.

- (non-parametric) Kendall’s Tau-b (τ*_b_*)
  - Disciplinarity Scale is positively associated with Years since PhD (τ*_b_*=0.13, p<0.001)
  - Disciplinarity Scale is positively associated with CDR Experience Scale (τ*_b_*=0.21, p<0.001)
- (parametric) Pearson’s correlation coefficient (*r*)
  - Disciplinarity Scale is positively associated with Years since PhD (*r*=0.20, p<0.001)
  - Disciplinarity Scale is positively associated with CDR Experience Scale (*r*=0.24, p<0.001)
- (parametric) one-way ANOVA (eta-squared or η²)
  - Disciplinarity Scale varies by Years since PhD: F(5, 4107)=35.05, p<0.001, η²=0.04; F(linear)=150.26, p<0.001
  - Disciplinarity Scale varies by CDR Experience Scale: F(4, 4108)=64.20, p<0.001, η²=0.06; F(linear)=207.29, p<0.001

The similarity of results across these different tests suggests these are robust relationships.

**Table A**: Results from One-Way ANOVA of Years since PhD on Five Dimensions of Disciplinarity

| **Year of PhD** | **# of Cases** | **substantive focus of primary department** | **professional association in which I am most active** | **substantive focus of journals in which I publish most often** | **discipline most peer scientists perceive me as representing** | **discipline I identify with most now** |
| --- | --- | --- | --- | --- | --- | --- |
| **before 1980** | 276 | 2.34 | 2.13 | 2.44 | 2.24 | 2.29 |
| **1980-1989** | 644 | 2.21 | 1.92 | 2.10 | 2.05 | 2.01 |
| **1990-1999** | 887 | 2.14 | 1.85 | 2.06 | 2.01 | 1.93 |
| **2000-2009** | 993 | 1.99 | 1.69 | 1.89 | 1.82 | 1.78 |
| **2010-2019** | 1085 | 1.99 | 1.58 | 1.75 | 1.72 | 1.69 |
| **2020-2024** | 228 | 1.68 | 1.33 | 1.56 | 1.49 | 1.40 |
| **F** (5, 4107) | | **12.01** | **26.65** | **32.02** | **24.93** | **30.08** |
| **p-value** | | **<.001** | **<.001** | **<.001** | **<.001** | **<.001** |
| **effect size** (η²) | | **.01** | **.03** | **.04** | **.03** | **.04** |

**Table B**: Results from One-Way ANOVA of Approximate Number of Cross-Disciplinary Research Projects on Five Dimensions of Disciplinarity

| **Approximate Number of CDR Projects** | **# of Cases** | **substantive focus of primary department** | **professional association in which I am most active** | **substantive focus of journals in which I publish most often** | **discipline most peer scientists perceive me as representing** | **discipline I identify with most now** |
| --- | --- | --- | --- | --- | --- | --- |
| **11+projects** | 897 | 2.31 | 2.06 | 2.37 | 2.23 | 2.16 |
| **7-10 projects** | 602 | 2.11 | 1.82 | 2.10 | 2.00 | 1.95 |
| **4-6 projects** | 963 | 2.09 | 1.72 | 1.95 | 1.87 | 1.85 |
| **1-3 projects** | 1250 | 1.91 | 1.58 | 1.71 | 1.70 | 1.65 |
| **0 projects** | 401 | 1.85 | 1.44 | 1.46 | 1.52 | 1.51 |
| **F** (4, 4108) | | **18.29** | **38.57** | **82.11** | **48.50** | **46.39** |
| **p-value** | | **<.001** | **<.001** | **<.001** | **<.001** | **<.001** |
| **effect size** (η²) | | **.02** | **.04** | **.07** | **.05** | **.04** |

**Table C**: Results from One-Way ANOVA of PhD Science Family on Five Dimensions of Disciplinarity

| **PhD**  **Science Family** | **# of Cases** | **substantive focus of primary department** | **professional association in which I am most active** | **substantive focus of journals in which I publish most often** | **discipline most peer scientists perceive me as representing** | **discipline I identify with most now** |
| --- | --- | --- | --- | --- | --- | --- |
| **biological and life sciences** | 1483 | 2.43 | 1.99 | 2.10 | 2.13 | 2.09 |
| **physical and earth sciences** | 1079 | 2.15 | 1.72 | 1.92 | 1.96 | 1.93 |
| **social and behavioral sciences** | 1551 | 1.65 | 1.52 | 1.80 | 1.59 | 1.54 |
| **F** (2, 4110) | | **178.44** | **78.78** | **31.21** | **107.93** | **119.30** |
| **p-value** | | **<.001** | **<.001** | **<.001** | **<.001** | **<.001** |
| **effect size** (η²) | | **.08** | **.04** | **.02** | **.05** | **.06** |

**Figure A**: Radar Figures for Scientists’ Five-Dimension Disciplinarity by PhD Science Discipline

**A. Legend	B. Biochemistry	C. Botany & Plant Science	D. Cell & Developmental Biology	E. Ecology & Evolutionary Biology	F. Env & Natural Resources Sciences	G. Genetics & Genomics**

primary department

|  | Genetics and Genomics |
| --- | --- |
| 1 | 2.5 |
| 2 | 2.27778 |
| 3 | 2.28571 |
| 4 | 2.2381 |
| 5 | 2.24603 |

|  | Environmental and Natural Resources Sciences |
| --- | --- |
| 1 | 2.3299 |
| 2 | 1.83505 |
| 3 | 2.07216 |
| 4 | 2.06186 |
| 5 | 1.83505 |

|  | Ecology and Evolutionary Biology |
| --- | --- |
| 1 | 2.48123 |
| 2 | 1.82423 |
| 3 | 1.97611 |
| 4 | 1.95904 |
| 5 | 1.94539 |

|  | Cell and Developmental Biology |
| --- | --- |
| substantive focus of primary department | 1.65 |
| professional association in which I am most active | 1.52 |
| substantive focus of journals in which I publish most often | 1.8 |
| discipline most peer scientists perceive me as representing | 1.59 |
| discipline I identify with most now | 1.54 |

|  | Botany and Plant Science |
| --- | --- |
| substantive focus of primary department | 2.125 |
| professional association in which I am most active | 1.84239 |
| substantive focus of journals in which I publish most often | 1.89674 |
| discipline most peer scientists perceive me as representing | 1.95652 |
| discipline I identify with most now | 1.89674 |

|  | Cell and Developmental Biology |
| --- | --- |
| substantive focus of primary department | 2.81159 |
| professional association in which I am most active | 2.5942 |
| substantive focus of journals in which I publish most often | 2.52899 |
| discipline most peer scientists perceive me as representing | 2.7029 |
| discipline I identify with most now | 2.61594 |

|  | Biochemistry |
| --- | --- |
| substantive focus of primary department | 2.58036 |
| professional association in which I am most active | 2.28348 |
| substantive focus of journals in which I publish most often | 2.35714 |
| discipline most peer scientists perceive me as representing | 2.41071 |
| discipline I identify with most now | 2.40179 |

professional association

self-identity

PhD

targeted journals

peer perceptions

**H. Microbiology	I. Neuroscience & Neurobiology	J. Zoology & Animal Sciences	K. Astronomy & Astrophysics	L. Chemistry	M. Geological & Earth Sciences	N. Ocean, Marine, & Atmos Sci**

|  | Ocean/Marine Sciences and Atmospheric Sciences |
| --- | --- |
| 1 | 2.41584 |
| 2 | 1.56436 |
| 3 | 1.79208 |
| 4 | 1.83168 |
| 5 | 1.83168 |

|  | Geological and Earth Sciences |
| --- | --- |
| 1 | 2.43925 |
| 2 | 1.63084 |
| 3 | 1.91121 |
| 4 | 1.95327 |
| 5 | 1.92056 |

|  | Chemistry |
| --- | --- |
| 1 | 2.04027 |
| 2 | 1.97483 |
| 3 | 2.21812 |
| 4 | 2.25168 |
| 5 | 2.14765 |

|  | Astronomy and Astrophysics |
| --- | --- |
| 1 | 2.03333 |
| 2 | 1.31905 |
| 3 | 1.2381 |
| 4 | 1.34286 |
| 5 | 1.4 |

|  | Zoology and Animal Sciences |
| --- | --- |
| 1 | 1.85906 |
| 2 | 1.55034 |
| 3 | 1.78523 |
| 4 | 1.80537 |
| 5 | 1.73826 |

|  | Neuroscience and Neurobiology |
| --- | --- |
| 1 | 2.41611 |
| 2 | 1.84564 |
| 3 | 1.98658 |
| 4 | 2.05369 |
| 5 | 2.03356 |

|  | Microbiology |
| --- | --- |
| 1 | 2.81301 |
| 2 | 1.89431 |
| 3 | 2.12195 |
| 4 | 2.11382 |
| 5 | 2.05691 |

**O. Physics	P. Anthropology	Q. Economics	R. Geography	S. Political Science	T. Psychology	U. Sociology**

|  | Sociology |
| --- | --- |
| 1 | 1.6195 |
| 2 | 1.62264 |
| 3 | 1.90881 |
| 4 | 1.59748 |
| 5 | 1.50629 |

|  | Psychology |
| --- | --- |
| 1 | 1.85127 |
| 2 | 1.65823 |
| 3 | 1.89082 |
| 4 | 1.73734 |
| 5 | 1.72152 |

|  | Political Science |
| --- | --- |
| 1 | 1.48193 |
| 2 | 1.43524 |
| 3 | 1.67771 |
| 4 | 1.53163 |
| 5 | 1.49699 |

|  | Geography |
| --- | --- |
| 1 | 2.21765 |
| 2 | 1.72941 |
| 3 | 2.36471 |
| 4 | 1.96471 |
| 5 | 1.78824 |

|  | Economics |
| --- | --- |
| 1 | 1.45687 |
| 2 | 1.31949 |
| 3 | 1.50479 |
| 4 | 1.33866 |
| 5 | 1.32588 |

|  | Anthropology |
| --- | --- |
| 1 | 1.72193 |
| 2 | 1.50802 |
| 3 | 1.92513 |
| 4 | 1.6631 |
| 5 | 1.61497 |

|  | Physics |
| --- | --- |
| 1 | 2.01524 |
| 2 | 1.71468 |
| 3 | 1.92521 |
| 4 | 1.92521 |
| 5 | 1.93352 |

**Supporting Text C**: Extended Discussion of the Substantive Focus of Respondent’s Primary Department

Of the five disciplinarity indicators, scientists’ agency seems the most constrained on the substantive focus of their primary department. They may exercise great autonomy in deciding which and how many professional associations to join and peer-reviewed journals to target. Further, the myriad of choices they make throughout their career likely influence how peers perceive them and how they view themselves, and such peer perceptions and self-identity may be rather fluid. Yet, scientists have far less control over the substantive focus of their primary academic department. This is due to a few factors. First, the hierarchical structures of major research universities are fairly similar due to organizational isomorphism. That is, most major research universities have largely the same array of discipline-based departments embedded in similarly structured colleges or schools. Second, there simply are not that many tenure-system science faculty positions in US research universities, so changing primary departments within universities is quite uncommon. Third, the US academic sector largely structures its labor market pools along disciplinary boundaries. Because of these factors, we are not surprised that the indicator for the substantive focus of respondents’ primary department has the weakest factor loading of the five.

**Supporting Text D**: Additional Information on Scientists Who Have Changed Their Discipline

The percentage of scientists who have changed their scientific discipline varies by Years since PhD, PhD Science Family, and CDR Experience.

- Years since PhD
  - % of scientists who have changed their science discipline
    - earned PhD after 2019: 7.0%
    - earned PhD between 2010 and 2019: 14.1%
    - earned PhD between 2000 and 2009: 17.0%
    - earned PhD between 1990 and 1999: 18.3%
    - earned PhD between 1980 and 1989: 19.3%
    - earned PhD before 1980: 21.4%
  - measures of association
    - Spearman’s Rank Coefficient: ρ=0.08, p<0.001
    - Kendall’s tau-b: τ*_b_*=0.07, p<0.001
    - Pearson’s correlation coefficient: *r*=0.08, p<0.001
    - one-way ANOVA: F=5.98, p<0.001, η²=0.01
- PhD Science Family
  - % of scientists who have changed their science discipline
    - biological and life sciences family: 29.5%
    - physical and earth sciences family: 14.7%
    - social and behavioral sciences family: 5.5%
  - measure of association
    - one-way ANOVA (F=172.64, p<0.001, η²=0.08
- CDR Experience
  - % of scientists who have changed their science discipline
    - 0 CDR projects: 5.24%
    - 1-3 CDR projects: 13.4%
    - 4-6 CDR projects: 16.7%
    - 7-10 CDR projects: 18.9%
    - 11+ CDR projects: 24.4%
  - measure of association
    - Spearman’s Rank Coefficient: ρ=0.14, p<0.001
    - Kendall’s tau-b: τ*_b_*=0.13, p<0.001
    - Pearson’s correlation coefficient: *r*=0.14, p<0.001
    - one-way ANOVA: F=22.54, p<0.001, η²=0.02

Beyond PhD science family, we also examined the percentages of scientists who have changed their discipline across the 20 science disciplines in our study. Fig B on the next page displays these percentages.

**Figure B**: Percentages of Respondents in 20 PhD Disciplines Who Changed Their Scientific Discipline (with 95% Confidence Interval)

|  | discipline |
| --- | --- |
| biochemistry | 45.5357% |
| cell and developmental biology | 43.4783% |
| genetics and genomics | 42.0635% |
| ecology and evolutionary biology | 27.6451% |
| microbiology | 21.9512% |
| zoology and animal sciences | 21.4765% |
| botany and plant science | 21.1957% |
| neuroscience and neurobiology | 18.7919% |
| environmental and natural resources sciences | 16.4948% |
| ocean/marine sciences and atmospheric sciences | 21.7822% |
| chemistry | 19.7987% |
| physics | 14.4044% |
| geological and earth sciences | 9.81308% |
| astronomy and astrophysics | 4.7619% |
| geography | 15.2941% |
| psychology | 8.5443% |
| anthropology | 7.48663% |
| economics | 3.51438% |
| political science | 3.31325% |
| sociology | 3.14465% |

**Table D**: Bivariate Correlations between Five Disciplinarity Dimensions and Nominal Discipline Change (N=4,113)

|  | respondent’s current discipline is different from their PhD discipline | substantive focus of my primary academic department | professional association in which I am most active | substantive focus of the journals in which I publish most often | discipline most peer scientists perceive me as representing |
| --- | --- | --- | --- | --- | --- |
| substantive focus of my primary academic department | .300 |  |  |  |  |
| professional association in which I am most active | .415 | .442 |  |  |  |
| substantive focus of the journals in which I publish most often | .397 | .414 | .665 |  |  |
| discipline most peer scientists perceive me as representing | .438 | .473 | .705 | .725 |  |
| discipline I identify with most now | .470 | .465 | .712 | .727 | .807 |

**Notes**: All correlations have a p-value of <0.001.
